# Fitting the Incidence Data from the City of Campinas, Brazil, Based on Dengue Transmission Modellings Considering Time-Dependent Entomological Parameters

**DOI:** 10.1371/journal.pone.0152186

**Published:** 2016-03-24

**Authors:** Hyun Mo Yang, José Luiz Boldrini, Artur César Fassoni, Luiz Fernando Souza Freitas, Miller Ceron Gomez, Karla Katerine Barboza de Lima, Valmir Roberto Andrade, André Ricardo Ribas Freitas

**Affiliations:** 1 Instituto de Matemática e Computação Científica (IMECC), Universidade Estadual de Campinas (UNICAMP), Campinas, SP, Brazil; 2 Superintendencia de Controle de Endemias (SUCEN), Campinas, SP, Brazil; 3 Coordenadoria de Vigilância Sanitária (COVISA), Campinas, SP, Brazil; 4 Instituto de Matemática e Computação (IMC), Universidade Federal de Itajubá (UNIFEI), Itajubá, MG, Brazil; Institut Pasteur, FRANCE

## Abstract

Four time-dependent dengue transmission models are considered in order to fit the incidence data from the City of Campinas, Brazil, recorded from October 1^*st*^ 1995 to September 30^*th*^ 2012. The entomological parameters are allowed to depend on temperature and precipitation, while the carrying capacity and the hatching of eggs depend only on precipitation. The whole period of incidence of dengue is split into four periods, due to the fact that the model is formulated considering the circulation of only one serotype. Dengue transmission parameters from human to mosquito and mosquito to human are fitted for each one of the periods. The time varying partial and overall effective reproduction numbers are obtained to explain the incidence of dengue provided by the models.

## Introduction

Dengue virus, a *flavivirus* transmitted by arthropod of the genus *Aedes*, is prevalent in different parts of the world. As a result of being pathogenic for humans and capable of transmission in heavily populated areas, dengue virus (arbovirus) can cause widespread and serious epidemics, which constitute one of the major public health problems in many tropical and subtropical regions of the world where *Aedes aegypti* and other appropriate mosquito vectors are present [1]. Additionally, the seasonality in these regions influences on the size of mosquito population, and also on the extrinsic incubation period due to the fact that mosquitoes belong to cold-blooded (poikilothermic) animals.

With respect to the seasonality of abiotic conditions, entomological parameters such as transition and mortality rates in aquatic phase, mortality rate of adult mosquito and oviposition rate vary with temperature [2][3][4]. On the other hand, there are scarce knowledge about the measurement of the effects of rainfall in mosquito population. Vezzani *et al*. [5] found the highest adult *A. aegypti* density with accumulated rainfalls above 150 *mm*. Micieli and Campos [6] observed a close relationship between the highest peak of *A. aegypti* population and high rainfall, and a decreasing in the population size for the months with less rainfall. Baruah and Dutta [7] observed a very high percentage of *A. aegypti* population with an annual average rainfall of 2758 *mm*, and the highest density was found in the post monsoon season. Moreover, they found that rainfall, when it is too heavy and continuous, may also be a regulating factor for *A. aegypti* proliferation (significant reduction of adult and washing away of immature stages), however heavy rainfall prepares the habitat for *Aedes* proliferation when the temperature is suitably high for mosquito proliferation. On the other hand, other authors [8][9] indicated that *Aedes* abundance would be mainly regulated by temperature rather than precipitation, however the same authors said that rainfall may be the only determining factor for *Aedes* proliferation where temperature is always above the marginal level.

Our aim in this work is the evaluation of the seasonally varying temperature and precipitation in dengue epidemics by a mathematical model. In the dengue transmission modelling, the mosquito population, which depends in a complex way on the abiotic factors, is coupled with the human population, which is assumed to be independent of abiotic variations, but allowed to vary in the size. The demographic data of the City of Campinas were retrieved from IBGE [10]. The model is then taken into account to estimate the incidence of dengue recorded from the City of Campinas, Brazil [11]. The daily recorded maximum and minimum temperatures and precipitation in the City of Campinas [12] are used to encompass the effects of temperature and precipitation on the incidence of dengue. These seasonally varying temperature and precipitation are captured by the time dependent model parameters related to the mosquito population.

With respect to the model, we assume that dengue virus is spread out by an appropriate encounter between mosquito and human populations: infected mosquitoes encounter and bite susceptible humans, or susceptible mosquitoes encounter and bite infectious humans, and the incidence rate captures the appearance of new cases of infectious individuals. In this paper, four different assumptions on this incidence rate are considered, which, for models with time varying parameters, lead to different predicted scenarios of dengue epidemics. Hence, a secondary goal of this work is the comparison of the adjusted curves of incidence yielded by four different assumptions considered in the formulation of the model.

The paper is structured as follows. In Materials and Methods, four non-autonomous models for dengue transmission are presented. This section also presents data (temperature, rainfall and dengue incidence) recorded from the City of Campinas, which are used by time dependent models in order to fit coefficients of transmission. Section Results presents the fitting of the models using incidence data from the City of Campinas. Finally, discussion and conclusion are given in section Discussion.

## Materials and Methods

Dengue virus circulates due to the interaction between human and mosquito populations in urban areas. Abiotic conditions affects not only the size of mosquito population, it also influences the circulation of virus inside the mosquito body—extrinsic incubation period, for example. A unique serotype of dengue virus is being considered in the modelling.

### Variables and parameters of mosquito population

The life cycle of *Aedes aegypti* encompasses an aquatic phase (eggs, larva and pupa) followed by winged (adult) form [4]. The number of eggs, which do not constitute a state variable (see [13] for a model including eggs compartment), is determined by the oviposition rate *φ*(*M*) = *ϕ*_*m*_*M*, where *ϕ*_*m*_ is the per-capita oviposition rate and *M*, the number of female mosquitoes at time *t*. Defining *L* as the number of larvae (female) at time *t*, the effective larvae production rate is given by *qf*[1−*L*/(*DC*)]*ϕ*_*m*_*M*, where we assume that a fraction *q* of eggs will hatch to the larva stage and a fraction *f* of these eggs become female mosquitoes, and *DC* is the total (carrying) capacity of the breeding sites. The constant parameter *D* represents the magnitude of the breeding sites, while *C* carries on the time varying abiotic conditions and demography of humans. The number of larvae decreases according to the change of larvae to pupae and death, described, respectively, by the changing *σ*_*l*_ and the mortality *μ*_*l*_ rates. The number of pupae at time *t*, designated by *P*, increases with the change of larvae to pupae (*σ*_*l*_) and decreases according to the transformation of pupae to adult mosquitoes and death, described, respectively, by the emerging *σ*_*p*_ and the mortality *μ*_*p*_ rates. Finally, the number of female mosquitoes increases according to the emerging of pupae (*σ*_*p*_) and decreases according to the mortality rate *μ*_*f*_.

Table 1 presents summary of parameters related to mosquito population and representative values. The value of *D* was chosen to be of the order of the magnitude of human population in the City of Campinas.

**Table 1:** Summary of mosquito population parameters and respective values.

Symbol	Meaning	Unit	Value
*f*	fraction of eggs originating female mosquitoes	-	0.5
*q*	fraction of eggs hatching to larva stage	-	0.5
*D*	magnitude of breeding sites	[*M*]	10^6^
*C*	varying breeding sites (carrying capacity)	-	0.8
*ϕ*_*m*_	intrinsic oviposition rate per female mosquito	*day*^−1^	4.0479
*σ*_*l*_	per-capita transition rate from larva to pupa	*day*^−1^	0.1184
*σ*_*p*_	per-capita transition rate from pupa to mosquito	*day*^−1^	0.3706
*μ*_*l*_	per-capita mortality rate of larva	*day*^−1^	0.0628
*μ*_*p*_	per-capita mortality rate of pupa	*day*^−1^	0.0573
*μ*_*f*_	per-capita mortality rate of mosquito	*day*^−1^	0.0373

Entomological parameters are calculated for 22°*C*. Dimension for mosquitoes, designated [*M*], refers to the number of mosquitoes. Eggs, larvae and pupae have same dimension as that for adult mosquitoes, [*M*].

The entomological parameters *ϕ*_*m*_, *σ*_*l*_, *σ*_*p*_, *μ*_*l*_, *μ*_*p*_ and *μ*_*f*_ clearly depend on temperature [3]. But, besides the temperature, the rainfall influences on the entomological parameters *μ*_*l*_ and *μ*_*p*_ (*μ*_*f*_ is practically insensitive [14]). The rainfall affects also on the carrying capacity *C* and the hatching fraction *q*. Let us describe the dependency of the model parameters on temperature and rainfall.

From a series of daily recorded maximum (*T*_max_) and minimum (*T*_min_) temperatures [12], we interpolated any temperature *T* at time *t* as

$$\begin{array}{c} T\left(t\right)=T\;\min\left(j\right)+[T\;\max(j)-T\;\min(j)]\sin\left(\pi t^{\prime }\right), \end{array}$$ (1)

where the index *j*, which is the integer part of time *t*, refers to the *j*-*th* calendar day, and *t*′ is the fractional part of time *t*, that is, 0 ≤ *t*′ < 1. Notably, *t*′ = 0 and *t*′ = 1 correspond to midnight, whereas *t*′ = 0.5 corresponds to midday.

To incorporate the rainfall in the model, we define *W*(*j*) as the amount of rainfall in *j*-*th* calendar day. The amount of rain precipitation influences on the mortality rates *μ*_*l*_ and *μ*_*p*_ in the aquatic phase and, also, on the carrying capacity (*C*) and the capacity of eggs being hatched (*q*).

During heavy rain periods, larvae and pupae can be flushed, thereby cleansing breeding sites of aquatic forms [15]. We refer to this situation as physically induced mortality, and we do not consider this type of mortality among adult mosquitoes [16].

From a series of daily recorded precipitation [12], we define the simplest physically induced mortality rates $\mu_{l}^{c}$ and $\mu_{p}^{c}$ as

$$\begin{array}{c} \left\{ \begin{array}{ccc} \mu_{l}^{c} & = & \mu_{l}\{1+g_{l}[W(j)-V_{c}]\theta [W(j)-V_{c}]\}\\ \mu_{p}^{c} & = & \mu_{p}\{1+g_{p}[W(j)-V_{c}]\theta [W(j)-V_{c}]\}, \end{array}\right. \end{array}$$

where *μ*_*l*_ and *μ*_*p*_ are temperature-dependent mortality rates, and *g*_*l*_ and *g*_*p*_ measure how the precipitation affects the additional physical mortality rates. Additionally, *V*_*c*_ is the critical rain volume sufficiently high to originate the overflow of breeding sites; *θ*(*x*) is the Heaviside function, that is, *θ*(*x*) = 1, if *x* ≥ 0 and is otherwise *θ*(*x*) = 0.

The fraction of eggs that are hatching (*q*) and the carrying capacity (*C*) are assumed to depend only on precipitation. We assume that the rain that has fallen in the past few days also influences both parameters.

For the fraction of eggs that hatch into larvae *q*, we follow similar argumentation given for the carrying capacity (see below). Because the relative humidity is almost 100% when there is abundant rain, we assume that

$$\begin{array}{c} q=\frac{q_{1}[W(j)+W_{M}]}{q_{0}+q_{1}[W(j)+W_{M}]}, \end{array}$$

where *q*_0_ is the capacity of eggs hatching with rainfall, *q*_1_ is the the critical amount of rain to promote hatching (the lower *q*_0_, the higher the influence of humidity on hatching). Notably, *q* = 1 when *W* → ∞. This type of Hill function was chosen to describe the saturating behavior [17].

The term *W*_*M*_ represents the rainfall of past days defined by

$$\begin{array}{c} W_{M}=\sum_{o=1}^{k}\frac{W(j-o)}{{\left\{w_{1}[T_{\max}(j-o)+T_{\min}(j-o)]\right\}}^{o}}, \end{array}$$

where *W*(*j* − *o*) is the rainfall in *j*-*o*-*th* days before, *w*_1_ is the residual effect of past rainfall. Observe that in the proposed expression for *W*_*M*_ the effect of past rain depends on the temperature since evaporation increases at higher temperatures; the parameter *w*_1_ regulates the past contributions in the sense that the higher *w*_1_, the lower the contribution of past rain due to evaporation. If *w*_1_ = 1/2, then the evaporation is a function of the daily mean temperature. Heavy and continuous rainfall may be a regulating factor for *A. aegypti* proliferation, as immature forms might have been washed away and thus significantly reduced the adult production. However, heavy rainfall prepares the habitat for *A. aegypti* proliferation in the post rainy season [7].

During the dry seasons, the number of breeding sites is dramatically reduced. We assume that the amount of rainfall always increases the breeding sites, even in the case of heavy rainfall (cleansing of breeding sites). Hence, for carrying capacity, we describe the influence of rainfall on its number as

$$\begin{array}{c} C=C_{2}+\frac{C_{0}[W(j)+W_{M}]}{C_{1}+W(j)+W_{M}}, \end{array}$$

where *C*_2_ is the rain independent variation in the breeding sites but can depend on time due to demographic changes in human population (not considered here), *C*_0_ is the capacity of rain in producing new breeding sites due to rain, and *C*_1_ is the the critical amount of rain in the formation of breeding sites (the lower *C*_1_, the higher the influence of rain on increasing breeding sites). Saturating behavior is again observed: *C* = *C*_2_ + *C*_0_ when *W* → ∞.

We now want to consider control efforts, such as insecticide application (chemical control) and the remotion of breeding sites (mechanical control). But, breeding sites can be removed in two ways: (1) remotion of breeding sites targeting houses surrounding case notification, and (2) preventive remotion to avoid dengue transmission. The total size of inhabitations in the City of Campinas [18] is approximately *n*_*T*_ = 350,000. We assume that the number of houses surveyed for breeding sites, and for further remotion, is proportional to decreasing in the carrying capacity, represented by

$$\begin{array}{c} C=\{C_{2}+\frac{C_{0}[W(j)+W_{M}]}{C_{1}+W(j)+W_{M}}\}(1-\alpha_{b}\frac{n_{b}}{n_{T}}-\alpha_{a}\frac{n_{a}}{n_{T}}), \end{array}$$

where *n*_*b*_ and *n*_*a*_ are the numbers of houses surveyed surrounding dengue cases, with *α*_*b*_ and *α*_*a*_ corresponding to the efficacy of mechanical control. Because the preventive visit is a random event (subscript *a*), whereas the dengue-targeting house visitation is case-search event (subscript *b*), we assumed that *α*_*b*_ > *α*_*a*_.

Spraying insecticide aims to kill adult mosquitoes, which is represented by

$$\begin{array}{c} \left\{ \begin{array}{ccc} \mu_{f}^{s} & = & \mu_{f}(1+\alpha_{s}\frac{n_{i}}{n_{T}})\\ \mu_{f}^{d} & = & \mu_{f}(1+\alpha_{d}\frac{n_{i}}{n_{T}}), \end{array}\right. \end{array}$$

where *n*_*i*_ is the number of houses where insecticide was sprayed surrounding dengue cases, and *μ*_*f*_ is the temperature-dependent mortality rate. Susceptible mosquitoes are distributed in all region, while the infectious mosquitoes are located in the neighborhood of infectious individuals. Hence, we assume that susceptible and infectious mosquitoes are under different additional mortalities. Defining *α*_*s*_ as the insecticide induced mortality among susceptible mosquitoes, and *α*_*d*_, the induced mortality among infectious mosquitoes, then *α*_*d*_ > *α*_*s*_.

Summarizing, the expressions for the carrying capacity, the hatching fraction and the entomological parameters are

$$\begin{array}{c} \left\{ \begin{array}{ccc} C & = & \left\{C_{2}+\frac{C_{0}[W(j)+W_{M}]}{C_{1}+W(j)+W_{M}}\}(1-\alpha_{b}\frac{n_{b}}{n_{T}}-\alpha_{a}\frac{n_{a}}{n_{T}}\right)\\ q & = & \frac{q_{1}[W(j)+W_{M}]}{q_{0}+q_{1}[W(j)+W_{M}]}\\ W_{M} & = & \sum_{o=1}^{k}\frac{W(j-o)}{{\left\{w_{1}[T_{\max}(j-o)+T_{\min}(j-o)]\right\}}^{o}}\\ \mu_{l}^{c} & = & \mu_{l}\{1+g_{l}[W(j)-V_{c}]\theta [W(j)-V_{c}]\}\\ \mu_{p}^{c} & = & \mu_{p}\{1+g_{p}[W(j)-V_{c}]\theta [W(j)-V_{c}]\}\\ \mu_{f}^{s} & = & \mu_{f}(1+\alpha_{s}\frac{n_{i}}{n_{T}})\\ \mu_{f}^{d} & = & \mu_{f}(1+\alpha_{d}\frac{n_{i}}{n_{T}}). \end{array}\right. \end{array}$$ (2)

Two extreme weather conditions are not considered in this modelling: monsoon season and long period of absence of rainfall. During monsoon season significant reduction of adult and washing away of immature stages are observed, which significantly reduces the breeding sites. In tropical and subtropical regions like the City of Campinas, heavy rains occur during few days in comparison with monsoon season. In our assumption, breeding sites are always increased with rain (which occurs during few hours or days), but immature stages are under physically induced mortality. During severe and prolonged drought period, people tend to storage water at home, sometimes in not well covered containers, and small bodies of water tend to dry and form multiple breeding sites. We are not considering this situation neither, which indeed occurred in the City of Campinas in the summer of 2015.

Table 2 presents a summary of parameters and representative values related to precipitation, and Table 3 presents a summary of parameters and representative values related to vector control. With respect to the vector control parameters in Table 3, the number of houses visited by public health agents was small. For this reason, they visit neighborhood of reported cases of dengue. Hence the effects on susceptible mosquitoes (distributed in a very large area) is assumed to be very small in comparison with that on infectious mosquitoes, which are located in the neighborhood of dengue cases. Hence, we attributed to the efficacy parameters plausible values based on the previous reasonings.

**Table 2:** Summary of mosquito population parameters depending on precipitation and respective representative values.

Symbol	Meaning	Unit	Value
*w*_1_	residual effect of past rain	1/°*C*	0.5
*C*_2_	rain independent variation in breeding sites	-	0.1
*C*_0_	production of breeding sites by rain	-	5
*C*_1_	critical amount of rain to produce breeding sites	*mm*	30
*q*_0_	capacity of eggs hatching with rain	-	0.2
*q*_1_	critical amount of rain to hatching	1/*mm*	0.02
*g*_*l*_	additional physical mortality among larvae	1/*mm*	0.0001
*g*_*p*_	additional physical mortality among pupae	1/*mm*	0.0001
*V*_*c*_	critical rain volume to overflow	*mm*	30.0

**Table 3:** Summary of mosquito population controlling parameters and respective representative values.

Symbol	Meaning	Unit	Value
*α*_*a*_	efficacy of preventive removing of breeding sites	-	20
*α*_*b*_	efficacy of targeted removing of breeding sites	-	100
*α*_*s*_	efficacy of insecticide application on susceptibles	-	2
*α*_*d*_	efficacy of insecticide application on infectious	-	1000

### Hypotheses on the dengue transmission

With respect to dengue transmission, the human population is divided into four compartments according to the natural history of the disease: *S*, *E*, *I* and *R*, which are the numbers at time *t* of, respectively, susceptible, exposed, infectious and recovered humans, with *S* + *E* + *I* + *R* = *N*, where *N* is the size of the human population. The fractions of human subpopulations are defined by the size of compartment divided by *N*, or *s* = *S*/*N*, *e* = *E*/*N*, *i* = *I*/*N* and *r* = *R*/*N*. The female mosquito population is divided into three compartments: *M*_1_, *M*_2_ and *M*_3_, which are the numbers at time *t* of, respectively, susceptible, exposed and infectious mosquitoes. The size of mosquito population is given by *M* = *M*_1_ + *M*_2_ + *M*_3_. By dividing *M*_1_, *M*_2_ and *M*_3_ by the magnitude *D*, for instance *m*_1_ = *M*_1_/*D*, we consider densities in order to have the same order of magnitude of fractions of sub-populations of humans. However, the fractions are defined by dividing by *M*; for instance, the fraction of susceptible mosquitoes is *M*_1_/*M* = *m*_1_/*m*. Hence, we stress the fact that *m*_1_ is the density of susceptible mosquitoes, while *m*_1_/*m* is the fraction, with *m* = *m*_1_ + *m*_2_ + *m*_3_.

Dengue transmission is sustained by the flows among human and mosquito compartments according to the dengue epidemics cycle. Susceptible humans are infected during the blood meal by infectious mosquitoes, with the force of infection (or per-capita incidence rate) being designated by *B*_*h*_. The exposed persons are, then, transferred to an infectious class by rate *γ*_*h*_, where 1/*γ*_*h*_ is the intrinsic incubation period. These infectious persons progress to recovered (immune) class at rate *σ*_*h*_. Neither loss of immunity nor induced mortality due to the disease are considered (a unique serotype infection). With respect to the vector, the susceptible mosquitoes are infected at a force of infection *B*_*m*_. These exposed mosquitoes are transferred to infectious class at a rate *γ*_*m*_, where 1/*γ*_*m*_ is the extrinsic incubation period, and remain infective until death.

The intrinsic incubation rate *γ*_*h*_ does not depend on ambient temperature, while the extrinsic incubation rate *γ*_*m*_ does. Following Lindsay and Birley [19], the extrinsic incubation rate depends on temperature according to

$$\begin{array}{c} \gamma_{m}^{-1}=\frac{T_{s}}{T-T_{m}}, \end{array}$$ (3)

where *T* is the ambient temperature (in degrees Celsius °*C*), *T*_*s*_ is the thermic sum (in °*C* × *day*), and *T*_*m*_ is the threshold of temperature below which dengue virus cannot multiply, hence *T* > *T*_*m*_.

The forces of infection (or per-capita incidence rates) *B*_*h*_ and *B*_*m*_ depend on the frequency of bites on humans by mosquitoes (assumed to be dependent on the per-capita oviposition rate *ϕ*_*m*_) and on the number (or fraction) of infective populations (designated by ϒ^*h*^ for humans and ϒ^*m*^ for mosquitoes). Hence, the forces of infection *B*_*h*_ and *B*_*m*_ can be written as *B*_*h*_ = *β*_*h*_*ϕ*_*m*_ϒ^*m*^ and *B*_*m*_ = *β*_*m*_*ϕ*_*m*_ϒ^*h*^, where *β*_*h*_ and *β*_*m*_ are the transmission coefficients (dimensionless with respect to time). Then, the transmission of dengue infections among susceptible individuals are given by

$$\begin{array}{c} \left\{ \begin{array}{ccc} \frac{d}{dt}M_{1} & = & \sigma_{p}P-(B_{m}+\mu_{f}^{s})M_{1}\\ \frac{d}{dt}S & = & \phi_{h}N-(B_{h}+\mu_{h})S, \end{array}\right. \end{array}$$

where *t* is in *days*. The demographic parameters *ϕ*_*h*_ and *μ*_*h*_ are, respectively, the natality and mortality rates. Different dependencies of the force of infection with infective populations ϒ^*h*^ and ϒ^*m*^ are studied.

First, the frequency dependent (FD) model is considered. The rate at which one mosquito bites one human can be assumed being directly proportional to the average biting rate of mosquitoes (intrinsic behavior of mosquitoes), and inversely proportional to the number of humans (one specific person has the chance of being bitten by one mosquito is reduced in a large population). In this situation, ϒ^*m*^ = *M*_3_/*N* and ϒ^*h*^ = *I*/*N*, and the forces of infection *B*_*h*_ and *B*_*m*_ are given by

$$\begin{array}{c} (\text{FD}):\{\begin{array}{ccc} B_{h} & = & \beta_{h}\phi_{m}\frac{M_{3}}{N}=\beta_{h}\phi_{m}\frac{D}{N}m_{3}\\ B_{m} & = & \beta_{m}\phi_{m}\frac{I}{N}=\beta_{m}\phi_{m}i, \end{array} \end{array}$$

and the dimensions of coefficients are: [*β*_*h*_] = [*M*]^−1^[*N*] and *β*_*m*_ is dimensionless with respect to populations. Notice that [*M*] and [*N*] are the numbers of mosquito and human populations. The magnitudes of mosquitoes and humans are similar, then *β*_*h*_ and *β*_*m*_ are the total transmission coefficients.

Second, pseudo mass action law (PMAL) model is considered. In this modelling the infection propagates due to the contact between the amount of human and mosquito populations, in which case ϒ^*m*^ = *M*_3_ and ϒ^*h*^ = *I*. The forces of infection *B*_*h*_ and *B*_*m*_ are given by

$$\begin{array}{c} (\text{PMAL}):\{\begin{array}{ccc} B_{h} & = & \beta_{h}\phi_{m}M_{3}=\beta_{h}\phi_{m}Dm_{3}\\ B_{m} & = & \beta_{m}\phi_{m}I=\beta_{m}\phi_{m}Ni, \end{array} \end{array}$$

and the dimensions of coefficients are: [*β*_*h*_] = [*M*]^−1^ and [*β*_*m*_] = [*N*]^−1^. Hence, *β*_*h*_ and *β*_*m*_ are the per-capita transmission coefficients.

Third, the true mass action law (TMAL) model is considered. In this case, the per-capita incidence rates depend on the fractions of infective populations, given by ϒ^*m*^ = *M*_3_/*M* and ϒ^*h*^ = *I*/*N*, and *B*_*h*_ and *B*_*m*_ are

$$\begin{array}{c} (\text{TMAL}):\{\begin{array}{ccc} B_{h} & = & \beta_{h}\phi_{m}\frac{M_{3}}{M}=\beta_{h}\phi_{m}\frac{m_{3}}{m}\\ B_{m} & = & \beta_{m}\phi_{m}\frac{I}{N}=\beta_{m}\phi_{m}i, \end{array} \end{array}$$

and the coefficients *β*_*h*_ and *β*_*m*_ are dimensionless with respect to populations, and they are total transmission coefficients.

Finally, a special incidence rate (SIR), or special mass action law, model is considered. In this modelling, the propagation of dengue is proportional to both fractions of susceptible and infectious populations. In this case, ϒ^*m*^ = *M*_3_/(*MN*),ϒ^*h*^ = *I*/(*MN*), and the per-capita incidence rates *B*_*h*_ and *B*_*m*_ are

$$\begin{array}{c} (\text{SIR}):\{\begin{array}{ccc} B_{h} & = & \beta_{h}\phi_{m}\frac{M_{3}}{MN}=\beta_{h}\phi_{m}\frac{1}{N}\frac{m_{3}}{m}\\ B_{m} & = & \beta_{m}\phi_{m}\frac{I}{MN}=\beta_{m}\phi_{m}\frac{1}{Dm}i, \end{array} \end{array}$$

and the incidence rates are proportional to fractions, or *m*_1_*i*/*m* and *sm*_3_/*m*. For this reason, the dimensions of coefficients are: [*β*_*h*_] = [*N*] and [*β*_*m*_] = [*M*]. The magnitudes of mosquitoes and humans are similar, then *β*_*h*_ and *β*_*m*_ are bi-total (product of the sizes of two populations) transmission coefficients.

Table 4 presents a summary of parameters and representative values related to dengue transmission and human population. The value of *γ*_*m*_ was obtained using Eq (3).

**Table 4:** Summary of dengue transmission and human population parameters, and respective representative values.

Symbol	Meaning	Unit	Value
*β*_*m*_	transmission rate from human to female mosquito	*	0.3
*β*_*h*_	transmission rate from female mosquito to human	*	0.06
*γ*_*s*_	thermic sum	°*C* × *day*^−1^	120
*T*_*m*_	threshold of temperature	°*C*	14
*γ*_*m*_	per-capita extrinsic incubation rate	*day*^−1^	0.05
*σ*_*h*_	per-capita recovery rate of human	*day*^−1^	0.1428
*γ*_*h*_	per-capita intrinsic incubation rate	*day*^−1^	0.1428
*ϕ*_*h*_	per-capita natality rate of human	*day*^−1^	7.310 × 10^−5^
*μ*_*h*_	per-capita mortality rate of human	*day*^−1^	3.805 × 10^−5^

The value of extrinsic incubation rate *γ*_*m*_ was calculated considering 22°*C*.

*The dimensions of *β*_*m*_ and *β*_*h*_ vary according to the definition of the incidence rate, and both are estimated.

### Dynamics of dengue transmission

We assume that the size of human population at time *t*, designated by *N*, obeys Malthus law. Hence, its size varies according to the difference between natality and mortality rates designated by *ϕ*_*h*_ and *μ*_*h*_, respectively, or

$$\begin{array}{c} \frac{d}{dt}N=(\phi_{h}-\mu_{h})N. \end{array}$$ (4)

Taking into account this equation, the equations for the size of sub-populations are related to the corresponding fractions. For instance, for the fraction of susceptibles *s*, we have

$$\begin{array}{c} \frac{d}{dt}s=\frac{d}{dt}\frac{S}{N}=\frac{1}{N}\frac{d}{dt}S-\frac{S}{N}\frac{1}{N}\frac{d}{dt}N, \end{array}$$

and using Eq (4), it is linked to the number of susceptibles *S* through

$$\begin{array}{c} \frac{d}{dt}s=\frac{1}{N}\frac{d}{dt}S-(\phi_{h}-\mu_{h})s, \end{array}$$

where equation for *S* was previously defined. The same procedure must be done for equations related to fractions *e* and *i*. But, for mosquito population, the numbers *L*, *P*, *M*_1_, *M*_2_ and *M*_3_ are divided by the magnitude *D*, for instance, the density *l* = *L*/*D*. Fractions and densities are dimensionless variables with respect to populations.

Based on the foregoing descriptions of the model parameters and variables, dengue transmission is described by two systems of differential equations. First system considers sub-populations where forces of infection do not appear, that is,

$$\begin{array}{c} (\text{Common}):\{\begin{array}{ccc} \frac{d}{dt}l & = & qf\phi_{m}m(1-\frac{l}{C})-(\sigma_{l}+\mu_{l}^{c})l\\ \frac{d}{dt}p & = & \sigma_{l}l-(\sigma_{p}+\mu_{p}^{c})p\\ \frac{d}{dt}m_{3} & = & \gamma_{m}m_{2}-\mu_{f}^{d}m_{3}\\ \frac{d}{dt}i & = & \gamma_{h}e-(\sigma_{h}+\phi_{h})i, \end{array} \end{array}$$ (5)

where the decoupled fraction of immune humans is given by *r* = 1 − *s* − *e* − *i*. The second system describes the susceptible and exposed populations according to the hypothesis related to the disease transmission: (1) for frequency dependent model (FD),

$$\begin{array}{c} (\text{FD}):\{\begin{array}{ccc} \frac{d}{dt}m_{1} & = & \sigma_{p}p-(\beta_{m}\phi_{m}i+\mu_{f}^{s})m_{1}\\ \frac{d}{dt}m_{2} & = & \beta_{m}\phi_{m}im_{1}-(\gamma_{m}+\mu_{f}^{d})m_{2}\\ \frac{d}{dt}s & = & \phi_{h}-(\beta_{h}\phi_{m}\frac{D}{N}m_{3}+\phi_{h})s\\ \frac{d}{dt}e & = & \beta_{h}\phi_{m}\frac{D}{N}m_{3}s-(\gamma_{h}+\phi_{h})e, \end{array} \end{array}$$ (6)

(2) for pseudo mass action law model (PMAL),

$$\begin{array}{c} (\text{PMAL}):\{\begin{array}{ccc} \frac{d}{dt}m_{1} & = & \sigma_{p}p-(\beta_{m}\phi_{m}Ni+\mu_{f}^{s})m_{1}\\ \frac{d}{dt}m_{2} & = & \beta_{m}\phi_{m}Nim_{1}-(\gamma_{m}+\mu_{f}^{d})m_{2}\\ \frac{d}{dt}s & = & \phi_{h}-(\beta_{h}\phi_{m}Dm_{3}+\phi_{h})s\\ \frac{d}{dt}e & = & \beta_{h}\phi_{m}Dm_{3}s-(\gamma_{h}+\phi_{h})e, \end{array} \end{array}$$ (7)

(3) for true mass action law model (TMAL),

$$\begin{array}{c} (\text{TMAL}):\{\begin{array}{ccc} \frac{d}{dt}m_{1} & = & \sigma_{p}p-(\beta_{m}\phi_{m}i+\mu_{f}^{s})m_{1}\\ \frac{d}{dt}m_{2} & = & \beta_{m}\phi_{m}im_{1}-(\gamma_{m}+\mu_{f}^{d})m_{2}\\ \frac{d}{dt}s & = & \phi_{h}-(\beta_{h}\phi_{m}\frac{m_{3}}{m}+\phi_{h})s\\ \frac{d}{dt}e & = & \beta_{h}\phi_{m}\frac{m_{3}}{m}s-(\gamma_{h}+\phi_{h})e, \end{array} \end{array}$$ (8)

and (4) for special incidence rate model (SIR),

$$\begin{array}{c} (\text{SIR}):\{\begin{array}{ccc} \frac{d}{dt}m_{1} & = & \sigma_{p}p-(\beta_{m}\phi_{m}\frac{1}{Dm}i+\mu_{f}^{s})m_{1}\\ \frac{d}{dt}m_{2} & = & \beta_{m}\phi_{m}\frac{1}{Dm}im_{1}-(\gamma_{m}+\mu_{f}^{d})m_{2}\\ \frac{d}{dt}s & = & \phi_{h}-(\beta_{h}\phi_{m}\frac{1}{N}\frac{m_{3}}{m}+\phi_{h})s\\ \frac{d}{dt}e & = & \beta_{h}\phi_{m}\frac{1}{N}\frac{m_{3}}{m}s-(\gamma_{h}+\phi_{h})e. \end{array} \end{array}$$ (9)

In all models, *β*_*h*_ and *β*_*m*_ are assumed to be constant. The entomological parameters *ϕ*_*m*_, *σ*_*l*_, *σ*_*p*_, *μ*_*l*_, *μ*_*p*_ and *μ*_*f*_ depend on temperature *T*, which is a function of time *t* according to Eq (1). The parameters $\mu_{l}^{c}$, $\mu_{p}^{c}$, $\mu_{f}^{s}$ and $\mu_{f}^{d}$ are the entomological parameters *μ*_*l*_, *μ*_*p*_ and *μ*_*f*_, respectively, encompassing rainfall and controlling efforts, whose expressions is given in Eq (2). The parameters *C* and *q* are dependent on precipitation. Therefore, all these parameters are function of time, and the dynamical systems are non-autonomous.

Summarizing, the first model (Model FD) considers the force of infection determined by biting probability, leading to a system formed by Eqs (5) and (6); the second model (Model PMAL) considers the pseudo mass action law, which gives the system formed by Eqs (5) and (7); the third model (Model TMAL) considers true mass action law, given the Eqs (5) and (8); the fourth model (Model SIR) considers the special incidence rate, which leads to Eqs (5) and (9). In all models, the dynamics of the human population is described by Eq (4).

### Effective reproduction numbers

In non-autonomous modelling, there are not well established epidemiological parameters to evaluate the risk of epidemics, such as the basic reproduction number. However, to a non-autonomous system, we can associate an autonomous by assuming constant parameters given by the mean values of the respective time-dependent parameters. The behaviors of the solutions of this associated autonomous system are then approximations of the dynamical behaviors of the solutions of the original system, at least for small time intervals; their long time behavior are also interesting to know since they inform what would be the dynamical tendencies if the parameters do not change in time. Therefore, by analyzing the special case where all model parameters are considered constant in time, we can borrow from autonomous modeling concepts, such as the basic reproduction number, and have at least some related information for the non-autonomous case.

In the particular case of constant model parameters and constant human and mosquito populations, the steady state of the autonomous model can be obtained. In S1 Text, from the steady states analysis of the autonomous dynamical system, the basic reproduction number *R*_0_, equation (S.10), is defined as the product of partial reproduction numbers $R_{0}^{m}$ and $R_{0}^{h}$, which, depending on the model, are given by equations (S.6), (S.7), (S.8) and (S.9). The values shown in Tables 1 to 4 can be taken as the representative values for the model parameters, which could be the mean values over one year.

From *R*_0_ and its partial reproduction numbers $R_{0}^{m}$ and $R_{0}^{h}$, let us defined the time varying effective reproduction number *R*_*ef*_ in terms of the partial effective reproduction numbers in humans ($R_{ef}^{h}$) and mosquitoes ($R_{ef}^{m}$) as

$$\begin{array}{c} R_{ef}=R_{ef}^{h}\times R_{ef}^{m}. \end{array}$$ (10)

(In the definitions for $R_{ef}^{h}$ and $R_{ef}^{m}$ below, consider that the model parameters and size of populations *N* and *m* are constant in time, but susceptible populations *s* and *m*_1_ are varying in time.) Suppose that at *t* = 0 one infectious case (does not matter if human or mosquito) is introduced in a completely susceptible populations of humans and mosquitoes (then, controls are absent). For *t* ≤ 0, before and just at the time of the beginning of epidemics, *R*_*ef*_ = *R*_0_ because *s* = 1 and *m*_1_ = *m* (then, $Dm_{1}^{*}=M^{*}$, corresponding to the trivial equilibrium). Notwithstanding, when *t* → ∞, the epidemics reaches a steady state, which occurs due to *R*_*ef*_ = 1, and equation (S.5) can be obtained, assuming that controls are applied.

In an autonomous modelling, the dynamical trajectory can be determined when one infectious human is introduced in a community free of dengue. The equilibrium point before the introduction of infectious case is given by *P*^0^, with equilibrium values of mosquito population being given by equation (S.1). Depending on the value of the basic reproduction number, the dengue disease fades out after a small epidemics (*R*_0_ < 1), or attains an endemic level (*R*_0_ > 1).

Now, in non-autonomous models developed here, the majority of model parameters (exceptions are *f*, *β*_*m*_, *β*_*h*_, and human related parameters *ϕ*_*h*_, *μ*_*h*_, *σ*_*h*_ and *γ*_*h*_) and the size of human and mosquito populations vary with time. Following the idea of the partial reproduction numbers obtained from the autonomous modelling, let us define the time varying effective reproduction number *R*_*ef*_ in terms of the partial effective reproduction numbers in humans ($R_{ef}^{h}$) and mosquitoes ($R_{ef}^{m}$) for our non-autonomous models.

For model FD, the partial effective reproduction numbers at any time *t* are defined by, using *M* = *Dm*,

$$\begin{array}{c} (\text{FD}):\{\begin{array}{ccc} R_{ef}^{h} & = & \frac{\beta_{h}\phi_{m}}{\mu_{f}^{d}}\frac{\gamma_{h}}{\gamma_{h}+\mu_{h}}s\\ R_{ef}^{m} & = & \frac{\beta_{m}\phi_{m}}{\sigma_{h}+\mu_{h}}\frac{\gamma_{m}}{\gamma_{m}+\mu_{f}^{d}}\frac{D}{N}m_{1}. \end{array} \end{array}$$ (11)

For model PMAL, the partial effective reproduction numbers at time *t* are

$$\begin{array}{c} (\text{PMAL}):\{\begin{array}{ccc} R_{ef}^{h} & = & \frac{\beta_{h}\phi_{m}}{\mu_{f}^{d}}\frac{\gamma_{h}}{\gamma_{h}+\mu_{h}}Ns\\ R_{ef}^{m} & = & \frac{\beta_{m}\phi_{m}}{\sigma_{h}+\mu_{h}}\frac{\gamma_{m}}{\gamma_{m}+\mu_{f}^{d}}Dm_{1}. \end{array} \end{array}$$ (12)

For model TMAL, the partial effective reproduction numbers at time *t* are

$$\begin{array}{c} (\text{TMAL}):\{\begin{array}{ccc} R_{ef}^{h} & = & \frac{\beta_{h}\phi_{m}}{\mu_{f}^{d}}\frac{\gamma_{h}}{\gamma_{h}+\mu_{h}}s\\ R_{ef}^{m} & = & \frac{\beta_{m}\phi_{m}}{\sigma_{h}+\mu_{h}}\frac{\gamma_{m}}{\gamma_{m}+\mu_{f}^{d}}\frac{m_{1}}{m}. \end{array} \end{array}$$ (13)

For model SIR, the partial effective reproduction numbers at time *t* are

$$\begin{array}{c} (\text{SIR}):\{\begin{array}{ccc} R_{ef}^{h} & = & \frac{\beta_{h}\phi_{m}}{\mu_{f}^{d}}\frac{\gamma_{h}}{\gamma_{h}+\mu_{h}}\frac{1}{Dm}s\\ R_{ef}^{m} & = & \frac{\beta_{m}\phi_{m}}{\sigma_{h}+\mu_{h}}\frac{\gamma_{m}}{\gamma_{m}+\mu_{f}^{d}}\frac{1}{N}\frac{m_{1}}{m}. \end{array} \end{array}$$ (14)

The partial effective reproduction numbers are evaluated at each time *t*.

The non-autonomous systems of equations depend on the temperature and rainfall, which assume different values in each time *t*. For this reason, the time dependent model parameters are presented below, which will be used in the numerical simulations of the four models named FD, TMAL, PMAL and SIR.

### Data collected from the City of Campinas

**Fig 1:**
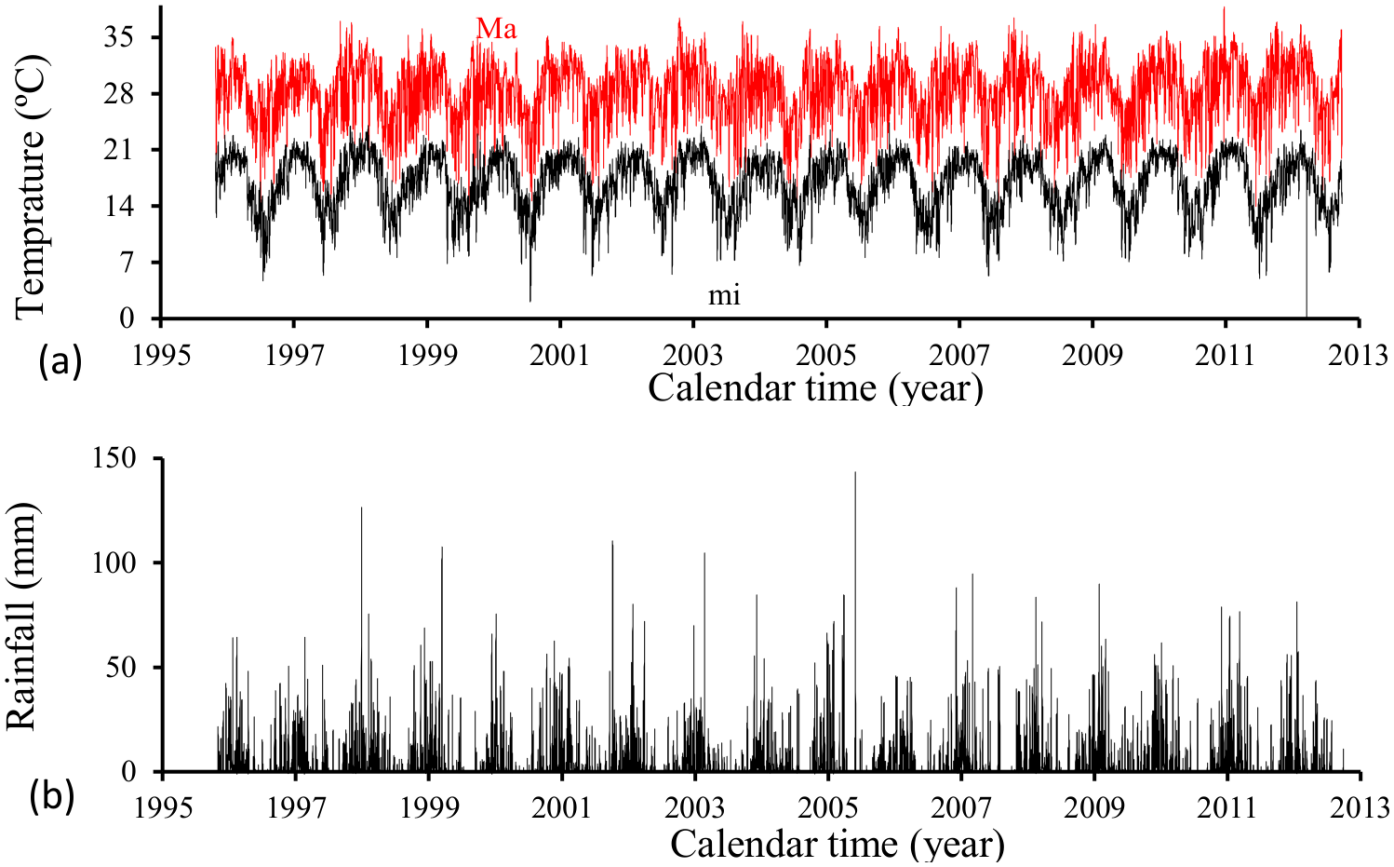
Daily maximum and minimum temperatures (a) and precipitation (b), from October 1^*st*^, 1995 to September 30^*th*^, 2012 in the City of Campinas, São Paulo State, Brazil.

The daily maximum and minimum temperatures and precipitation [12] from October 1^*st*^, 1995 to September 30^*th*^, 2012 in the City of Campinas, São Paulo State, Brazil, are used to numerically simulate the previously described dynamics systems. Fig 1 shows those data, with calendar year varying from 1995 to 2013 in the time axis: Fig 1(a) shows the maximum (*Ma*) and minimum (*mi*) temperatures, and Fig 1(b), the precipitation.

From Fig 1(a), the maximum temperatures oscillate between 16 and 37°*C*, while the minimum, between 4 and 24°*C*. A mean upper amplitude of the precipitation situates around 50 *mm*, and in each year some peaks of rainfall were observed, with the highest peak situating near 145 *mm* (see Fig 1(b)).

From a series of daily recorded maximum (*T*_max_) and minimum (*T*_min_) temperatures [12], shown in Fig 1(a), we interpolate any temperature *T* at time *t* according to Eq (1).

**Fig 2:**
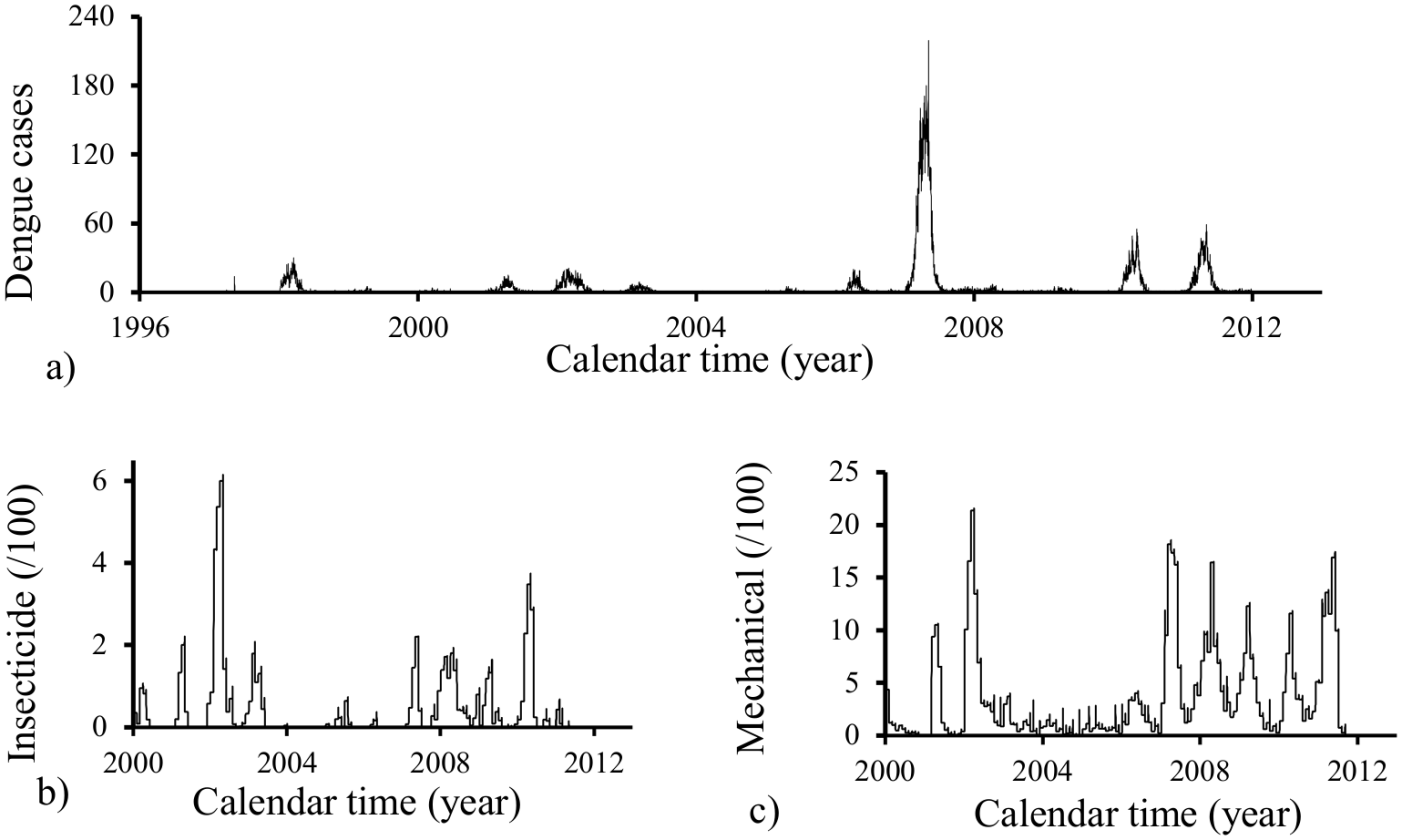
Monthly recorded cases of dengue in the City of Campinas from October 1^*st*^, 1995 to September 30^*th*^, 2012 (a), and the controlling of mosquitoes by spraying insecticide and/or removing of the breeding sites since 2010 (b,c).

Fig 2 shows the monthly recorded cases of dengue in the City of Campinas [11] from October 1^*st*^, 1995 to September 30^*th*^, 2012 (a), and the controlling of mosquitoes by spraying insecticide and/or removing of the breeding sites [20] since 2010 (b,c). The monthly data shown in Fig 2(a) are the cumulative dengue cases during each month [11].

By observing Fig 2(a), the period of time (October) 1995–2012 (September) can be roughly clustered in four periods: (October) 1995–2000 (September), (October) 2000–2004 (September), (October) 2004–2008 (September) and (October) 2008–2012 (September). The worst epidemics occurred in the third period. The possible circulating serotypes can be determined in these four periods based on serological screen in a small fraction of total cases. In the first period of time, serotype 1 predominated (96.4%), and serotype 2 was 3.6%. In the second period of time, serotype 1 predominated (72.2%), and serotype 3 was 27.8%. In the third period of time, only serotype 3 circulated (100%). In the last period, serotype 1 predominated (76.7%), followed by serotype 2 (16.7%) and by serotypes 3 and 4 (3.3% each). No serotype were found in years 2000, 2004 and 2008, which was another reason to grouping in the previous four periods. Fig 2(b) shows the number of houses visited to spray insecticides, and Fig 2(c), remotion of breeding sites. In the second period, the decreased cases of dengue could be explained by intense controlling efforts, while the third period presented highest epidemics due to the controlling efforts have been applied after the beginning of epidemics. Another factor to explain this elevated cases of dengue is the massive infection by serotype 3, which was already circulating in the second period.

Population in the City of Campinas varied from 1991 (847,595) to 2010 (1,080,999) [10]. This varying population was taken into account estimating the values of *ϕ*_*h*_ and *μ*_*h*_ using Eq (4). The estimated values were given in Table 4. The total size of inhabitations in City of Campinas is approximately *n*_*T*_ = 350,000 [18].

### Time dependent entomological parameters

Based on the temperature and rainfall shown in Fig 1, time dependent parameters related to mosquito population are presented. The temperature dependent entomological parameters *ϕ*_*m*_, *μ*_*l*_, *μ*_*p*_, *μ*_*f*_, *σ*_*l*_ and *σ*_*p*_ of *A. aegypti* are obtained using fittings given in Table 5 [2][3][4]. An *n*-*th* degree polynomial *P*_*n*_(*T*) = *b*_0_ + *b*_1_*T* + ⋯ + *b*_*n*_*T*^*n*^ was used to fit experimental data.

**Table 5:** Estimated entomological parameters of female mosquito and aquatic phase.

Coef.	*ϕ*_*m*_	*μ*_*l*_	*μ*_*p*_	*μ*_*f*_	*σ*_*l*_	*σ*_*p*_
*b*_0_	-5.3999	2.31532	4.25 × 10^−1^	8.69 × 10^−1^	-1.84270	21.9021
*b*_1_	1.800160	−4.19 × 10^−1^	−3.25 × 10^−2^	−1.59 × 10^−1^	8.29 × 10^−1^	-10.31110
*b*_2_	−2.12 × 10^−1^	2.73 × 10^−2^	7.06 × 10^−4^	1.12 × 10^−2^	−1.46 × 10^−1^	2.050830
*b*_3_	1.02 × 10^−2^	−7.53 × 10^−4^	4.39 × 10^−7^	−3.41 × 10^−4^	1.31 × 10^−2^	−2.24 × 10^−1^
*b*_4_	−1.51 × 10^−4^	7.50 × 10^−6^	-	3.81 × 10^−6^	−6.46 × 10^−4^	1.47 × 10^−2^
*b*_5_	-	-	-	-	1.79 × 10^−5^	−5.89 × 10^−4^
*b*_6_	-	-	-	-	−2.62 × 10^−7^	1.41 × 10^−5^
*b*_7_	-	-	-	-	1.5 × 10^−9^	−1.9 × 10^−7^
*b*_8_	-	-	-	-	-	1.0 × 10^−9^

These parameters were estimated using an *n*-*th* degree polynomial, with unit of coefficients *b*_*i*_ being *days*^−1^ × (°*C*)^−*i*^. Standard deviations are not shown.

**Fig 3:**
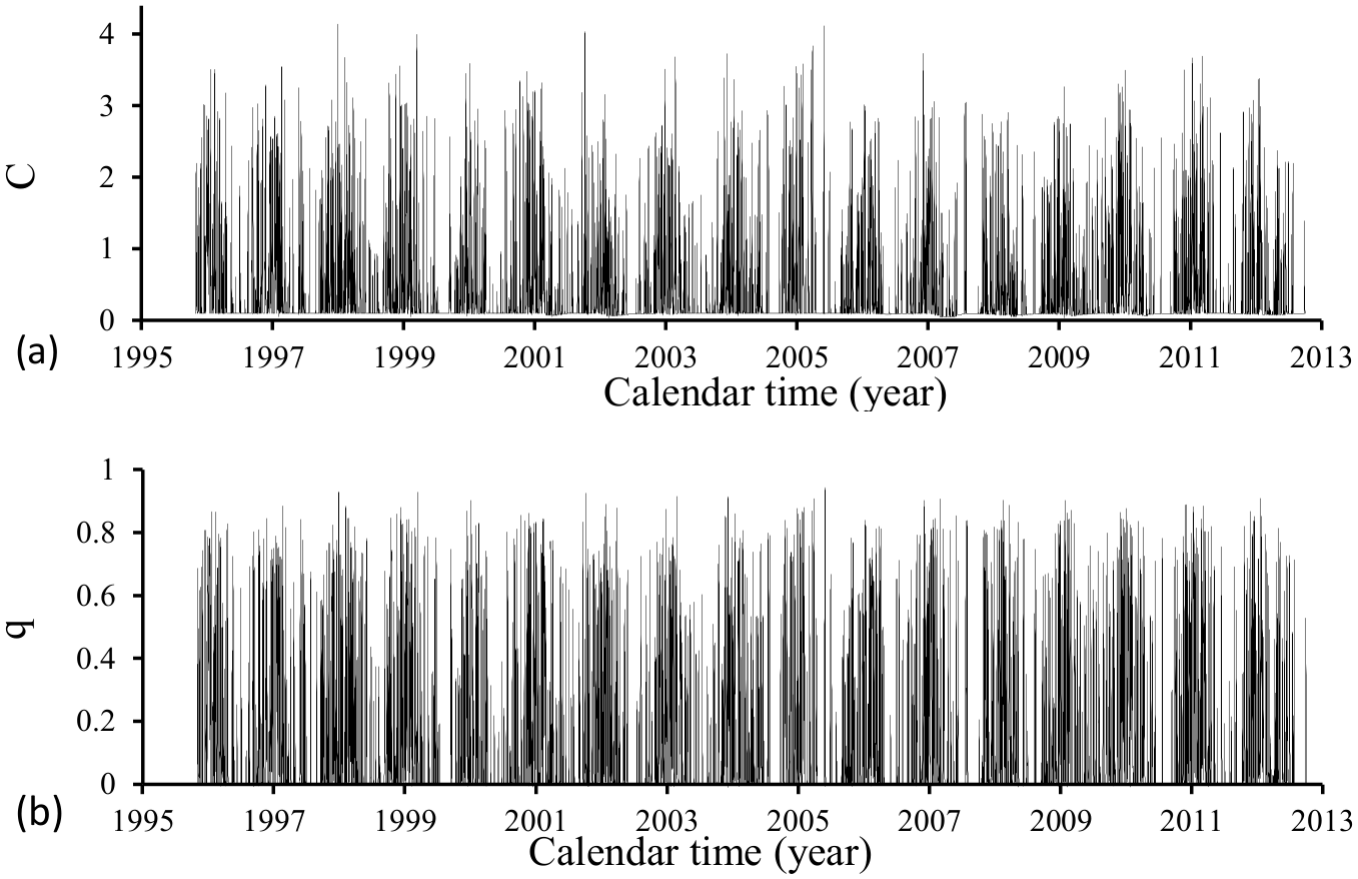
Time varying carrying capacity *C* (a) and the fraction of hatching eggs *q* (b) during the period of time (October) 1995–2012 (September).

Fig 3 shows the time varying carrying capacity *C* (a) and the fraction of hatching eggs *q* (b) during the period of time (October) 1995–2012 (September). These parameters are allowed to dependent only on rainfall.

The carrying capacity *C* and the fraction of eggs hatching *q* shown in Fig 3 were obtained using Eq (2) and the volume of rainfall shown in Fig 1(b). The values of the parameters dependent on the precipitation were given in Table 2.

**Fig 4:**
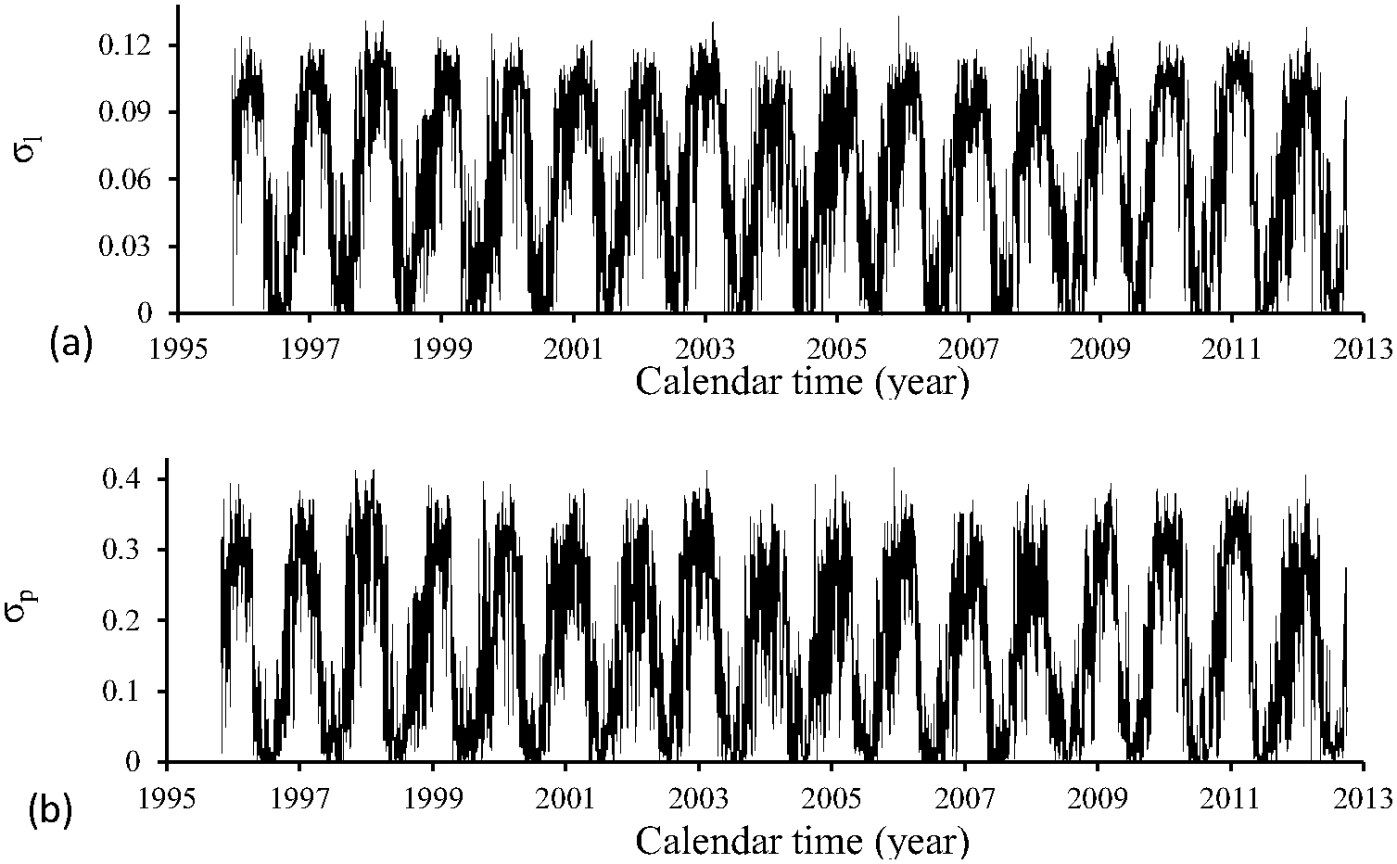
Transition rates *σ*_*l*_ (a) and *σ*_*p*_ (b) during the period of time (October) 1995–2012 (September).

Fig 4 shows the transition rates *σ*_*l*_ (a) and *σ*_*p*_ (b) during the period of time (October) 1995–2012 (September). These parameters are allowed to depend only on temperature.

The transition rates *σ*_*l*_ and *σ*_*p*_ were obtained using polynomials fitted given in Table 5, and the temperature at each time *t* was calculated using Eq (1), with maximum and minimum daily temperatures being shown in Fig 1(a).

**Fig 5:**
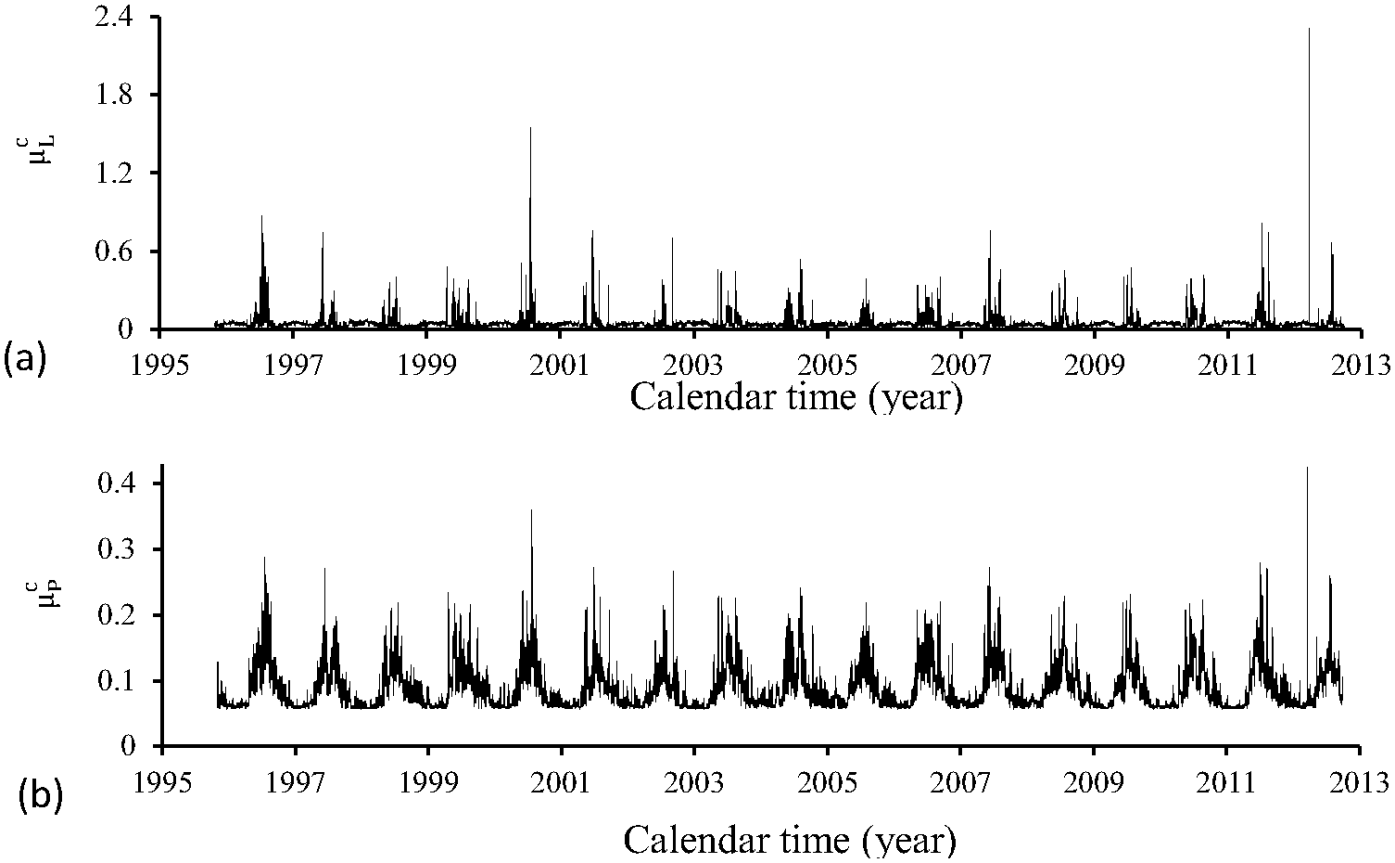
Overall mortality rates of aquatic forms $\mu_{l}^{c}$ (a) and $\mu_{p}^{c}$ (b) during the period of time (October) 1995–2012 (September).

Fig 5 shows the overall mortality rates of aquatic forms $\mu_{l}^{c}$ (a) and $\mu_{p}^{c}$ (b) during the period of time (October) 1995–2012 (September). These parameters are allowed to depend on temperature and rainfall.

The temperature dependent mortality rates of aquatic forms *μ*_*l*_ and *μ*_*p*_ were obtained using polynomials fitted given in Table 5, and the temperature at each time *t* was calculated using Eq (1), with maximum and minimum daily temperatures being shown in Fig 1(a). The contribution of the rainfall was calculated by Eq (2) and the volume of rainfall is shown in Fig 1(b). The values of the parameters dependent on the precipitation were given in Table 2.

**Fig 6:**
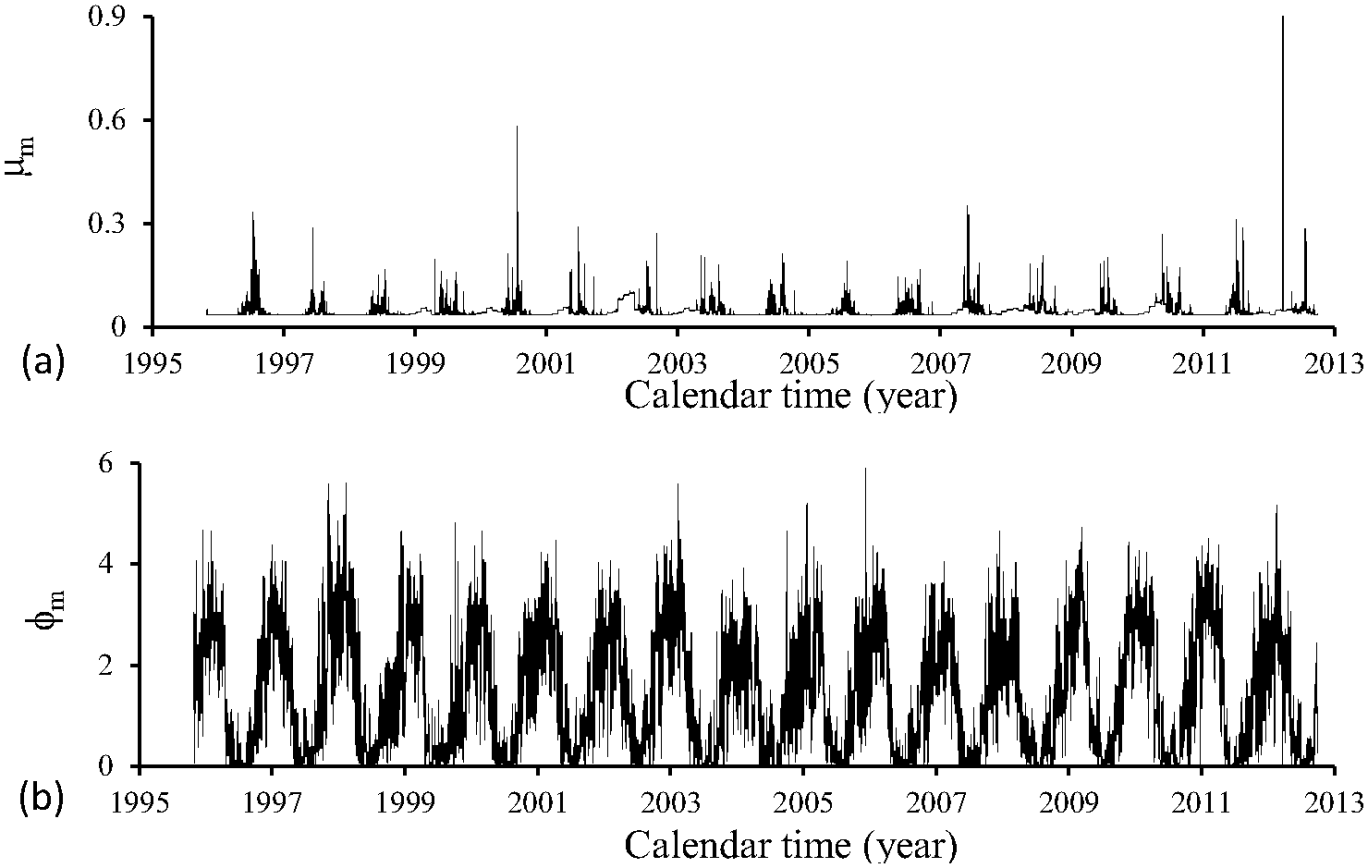
Mortality rate of adult mosquitoes *μ*_*m*_ (a) and the oviposition rate *ϕ*_*m*_ (b) during the period of time (October) 1995–2012 (September).

Fig 6 shows the mortality rate of adult mosquitoes *μ*_*f*_ (a) and the oviposition rate *ϕ*_*m*_ (b) during the period of time (October) 1995–2012 (September). These parameters are allowed to depend only on temperature.

The temperature dependent mortality rate of adult mosquitoes *μ*_*f*_ and the oviposition rate *ϕ*_*m*_ were obtained using polynomials fitted given in Table 5, and the temperature at each time *t* was calculated using Eq (1), with maximum and minimum daily temperatures being shown in Fig 1(a).

Figs 3 to 6 show clearly the model parameters depending on temperature and also on precipitation. In unfavorable seasons, there is a dramatic decrease in these parameters.

### Estimation method

In the foregoing subsection, the model parameters related to mosquito (time dependent) and human (time independent) populations were obtained. Time-dependent model parameters were shown graphically. The simulations done in Yang *et al.* [14] only gave us certain intervals where probably the values of *β*_*m*_ and *β*_*h*_ are located. In the next section, the transmission coefficients *β*_*m*_ and *β*_*h*_ are fitted for the dengue incidence recorded in the City of Campinas. To obtain the estimated values of *β*_*m*_ and *β*_*h*_, it is necessary to appeal to a suitable numerical algorithm for parameter identification.

In order to estimate *β*_*m*_ and *β*_*h*_, it is natural to start by considering a weighted discrete *L*^2^-error; thus, a possible functional *Er* to be minimized could be:

$$\begin{array}{c} Er\left(X\right)={\left\{\sum_{j=1}^{k}{\left[|i\left(t_{j}\right)-i_{obs}\left(t_{j}\right)|p\left(\lambda ,t_{j}\right)\right]}^{2}\right\}}^{1/2}, \end{array}$$ (15)

where

1. *X* = (*β*_*m*_, *β*_*h*_, *prop*) contains the three parameters to be fitted;
2. *t*_*j*_, *j* = 1, ⋯, *k*, where *k* is the size of sample, is the recorded number of cases of dengue at time *t*_*j*_ in the City of Campinas (remember, *t* is in *days*);
3. *i*(*t*_*j*_) is the value of the fraction of infected individuals obtained by the numerical simulation of the model using the values of *β*_*m*_ and *β*_*h*_ given in the *X* where the error *Er*(*X*) is being evaluated.
4. *i*_*obs*_(*t*_*j*_) is the fraction of the expected infected individuals, which is computed as
  $$\begin{array}{c} i_{obs}\left(t_{j}\right)=\frac{prop\times cases(t_{j})}{N(t_{j})}, \end{array}$$
  where *cases*(*t*_*j*_), for *t*_1_, ⋯, *t*_*N*_, are the recorded cases of dengue in the City of Campinas, and *N*(*t*_*j*_) is the size of the human population. The additional parameter *prop* encompasses all cases of dengue which were not detected (asymptomatic individuals) [21]; the value of this parameter must also be found during the fitting process.
5. *p*(*λ*, *t*_*j*_) is the weighting factor that depends on a pre-established parameter *λ* ≥ 1; such weighting factor could be, for instance:
  $$\begin{array}{c} p\left(\lambda ,t_{j}\right)=1+\left(\lambda -1\right)\frac{i_{obs}\left(t_{j}\right)}{\max_{j}|i_{obs}\left(t_{j}\right)|}. \end{array}$$ (16)
  In this case, we observe that by choosing large value of *λ*, one can favor the days with more infected people in the process of fitting the data; *λ* = 1 attributes equal weights to the given data;

The transmission coefficients *β*_*m*_ and *β*_*h*_, and the term *prop* are then estimated using confirmed dengue cases. In the procedure of the estimation method, we need to provide the intervals where these parameters are located as

$$\begin{array}{c} \left\{ \begin{array}{c} \;\text{Interval}\;\text{where}\;\beta_{m}\;\text{is}\;\text{probably}\;\text{located:}\;\left[a,b\right]\\ \;\text{Interval}\;\text{where}\;\beta_{h}\;\text{is}\;\text{probably}\;\text{located:}\;\left[c,d\right]\\ \;\text{Interval}\;\text{where}\;prop\;\text{is}\;\text{probably}\;\text{located:}\;[A,B]. \end{array}\right. \end{array}$$ (17)

In S1 Text, the estimation method is described in detail.

With respect to observed data, the recorded cases of dengue in the City of Campinas are cumulative number of dengue cases *I*_*obs*_(*t*_*j*_) in each month. Hence, *i*_*obs*_(*t*_*j*_) = *I*_*obs*_(*t*_*j*_)/30, where *I*_*obs*_(*t*_*j*_) is given in Fig 2(a), and *t*_*j*_ is the mean calendar time of each month.

The recorded cases of dengue in the City of Campinas during 1995–2012 were separated in four periods of time, named: Period 1 (1995–2000), Period 2 (2000–2004), Period 3 (2004–2008) and Period 4 (2008–2012), and each period begins at month October and ends at September. The first period encompasses five years, while the others, four years, and the numbers of recorded cases of dengue in the City of Campinas for the four periods are: *k*_1_ = 60 and *k*_2_ = *k*_3_ = *k*_4_ = 48. for each period we estimate the parameters *β*_*m*_, *β*_*h*_ and *prop*.

Due to the characteristics of our data, in the actual fitting procedure, we used a weighting factor distinct of the one described by Eq (16). In fact, it is observed a pattern in the incidence cases in each year (see Fig 2(a)): in October initiates the increasing phase, which is followed by decreasing up to September. Hence, let us define the annual epidemiological cycle comprised between October 1^*st*^ and September 30^*th*^ of the next year. In each annual epidemiological cycle, the peak of incidence can vary broadly. Then, instead of a maximum incidence in the whole period of estimation, maximum in each annual cycle max_*j*_|*i*_*obs*_(*t*_*j*_)|_*g*_, for *g* = 1, ⋯, *u*, where *u* is the number of annual cycles in this period, is determined. In this case, a possible weighting factor could be

$$\begin{array}{c} \begin{array}{ccc} p_{g}\left(\lambda ,t_{j}\right)=1+\left(\lambda -1\right)\frac{i_{obs}\left(t_{j}\right)}{\max_{{}_{j}}{|i_{obs}\left(t_{j}\right)|}_{g}}, & for & g=1,\cdots ,u, \end{array} \end{array}$$

and *j* = 1, ⋯, *j*_*g*_, where *j*_*g*_ is the number of recorded dengue cases in the annual cycle *g*, with $\sum_{g=1}^{u}j_{g}=k_{l}$, where *k*_*l*_ is the number of recorded dengue cases in the *l*-*th* period.

Additional modifications to the weighting factor *p*_*g*_(*λ*, *t*_*j*_) are done as follows. In an annual epidemiological cycle, all observed points contribute with the same weight, then *λ* = 1 and *p*_*g*_(*λ*, *t*_*j*_) = 1. However, it is possible to enhance the importance of the maximum dengue case in each annual cycles for the fitting process. Let us define ${\hat{t}}^{g}$, for *g* = 1, ⋯, *u*, as the calendar time when occurred the maximum recorded dengue cases in the *g*-*th* annual cycle. But this maximum may occur at different time than that yielded by the model, which is denoted by *t*^*g*^, for *g* = 1, ⋯, *u*. In the error function, besides the equal contribution of all samples, we include the difference between the peaks of theoretical and observed number of cases ($|i\left(t^{g}\right)-i_{obs}\left({\hat{t}}^{g}\right)|$) and, also, the time when both peaks occurred ($|{\bar{t}}^{g}-t^{g}|$). In the minimization process, we impose that the difference between the peaks *i*(*t*^*g*^) and $i_{obs}\left({\hat{t}}^{g}\right)$ must be very close, then weight *λ*_1_ > 1 is associated to it. On the other hand, the times when the peaks occur must be also very close, then we introduce the penalty factor *λ*_2_. In this case, the error function Eq (15) becomes

$$\begin{array}{c} \begin{array}{ccc} Er(X) & = & {\left\{\sum_{g=1}^{u}\sum_{j=1}^{j_{g}}{|i\left(t_{j}\right)-i_{obs}\left(t_{j}\right)|}^{2}+\sum_{g=1}^{u}{\left[|i\left(t^{g}\right)-i_{obs}\left({\hat{t}}^{g}\right)|\frac{i_{obs}\left({\hat{t}}^{g}\right)}{\sum_{g=1}^{u}i_{obs}\left({\hat{t}}^{g}\right)}\lambda_{1}\right]}^{2}\right\}}^{1/2}\\ & & +{\left\{\sum_{g=1}^{u}{\left[|{\hat{t}}^{g}-t^{g}|\lambda_{2}\right]}^{2}\right\}}^{1/2}, \end{array} \end{array}$$ (18)

where the second term is the penalty when the times between observed and theoretical peaks are distant.

During the estimation procedure, the dynamical system of equations are simulated by providing initial conditions. The goal of an arbitrary initial condition is to eliminate the transient effects on the dynamics of mosquito population. For this reason the initial conditions at *t* = *t*_0_ for all models are the same, given by

$$\begin{array}{c} (l=0.5,p=0.2,m_{1}=1.0,m_{2}=0,m_{3}=0,s=N_{0},e=0,i=0), \end{array}$$ (19)

where *t*_0_ corresponds to January 1^*st*^, 1991, and *N*_0_ = 847,595.

Dengue infection is assumed to be introduced in October 1^*st*^ of years 1995, 2000, 2004 and 2008, respectively named periods 1, 2, 3 and 4. The introduction of infectious individuals is described by, for instance for FD model,

$$\begin{array}{c} (\text{FD}):\{\begin{array}{ccc} \frac{d}{dt}s & = & \phi_{h}-(\beta_{h}\phi_{m}\frac{D}{N}m_{3}+\phi_{h})s-i_{d}\delta (t-t_{d})\\ \frac{d}{dt}i & = & \gamma_{h}e-(\sigma_{h}+\phi_{h})i+i_{d}\delta (t-t_{d}), \end{array} \end{array}$$

where *δ*(*t* − *t*_*d*_) is the Dirac delta function, and *i*_*d*_ = *I*_*d*_/*N*_*d*_, with *N*_*d*_ being the size of human population at time *t*_*d*_. In other words, a number *I*_*d*_ is transferred from susceptible population to infectious population at exactly *t* = *t*_*d*_. This instantaneous transference is captured by the following boundary conditions at *t* = *t*_*d*_ given by

$$\begin{array}{c} (l=l_{d},p=p_{d},m_{1}=m_{d},m_{2}=0,m_{3}=0,s=1-i_{d},e=0,i=i_{d}), \end{array}$$ (20)

where *l*_*d*_ = *l*(*t*_*d*_), *p*_*d*_ = *p*(*t*_*d*_) and *m*_1*d*_ = *m*_1_(*t*_*d*_). The boundary conditions given by Eq (20), however, are taken at different times. The boundary conditions correspond to four distinct and uncorrelated epidemics (this strong assumption can be weakened by introducing more than one serotypes in a future modeling).

For each period, we estimate the parameters *β*_*m*_, *β*_*h*_ and *prop* considering the four models. We show, using the estimated parameters *β*_*m*_ and *β*_*h*_, the following figures: (1) fraction of cases of dengue multiplied by the term *prop*, that is, *prop* × *cases*(*t*_*j*_), and model based fraction *i*; (2) the proportion of infectious mosquitoes *m*_3_/*m*; (3) the partial effective reproduction numbers $R_{ef}^{h}$ and $R_{ef}^{m}$; and (4) the effective reproduction number *R*_*ef*_. The effective reproduction number *R*_*ef*_ is given by Eq (10), with the partial effective reproduction numbers $R_{ef}^{h}$ and $R_{ef}^{m}$ being given by Eqs (11), (12), (13) and (14), respectively for models FD, TMAL, PMAL and SIR.

The mean value for the effective reproduction number *R*_*ef*_ over the period of time [*t*_1_, *t*_2_], denoted by ${\bar{R}}_{ef}$, is calculated by

$$\begin{array}{c} {\bar{R}}_{ef}=\frac{1}{t_{2}-t_{1}}\int_{t_{1}}^{t_{2}}R_{ef}dt, \end{array}$$ (21)

where *t*_1_ and *t*_2_ are the calendar year when one of the periods of estimation begins and ends, respectively. Similarly, the mean values for the partial effective reproduction numbers $R_{ef}^{h}$ and $R_{ef}^{m}$, denoted respectively by ${\bar{R}}_{ef}^{h}$ and ${\bar{R}}_{ef}^{m}$, can be calculate.

### Data and data sources

We present a brief description of data and data source.

- Number of inhabitants in the City of Campinas is found at Instituto Brasileiro de Geografia e Estatística (IBGE): http://www.censo2010.ibge.gov.br/sinopse/index.php?dados=21&uf=35, publicly available.
- Number of houses in the City of Campinas is found at Instituto Brasileiro de Geografia e Estatística (IBGE): http://www.censo2010.ibge.gov.br/sinopse/index.php?uf=35&dados=212, publicly available.
- Daily recorded temperature (maximum and minimum) and precipitation in the City of Campinas are found at Sistema de Monitoramento Agrometeorológico (Agritempo): http://www.agritempo.gov.br/agritempo/jsp/PesquisaClima/index.jsp?siglaUF=SP, publicly available.
- Monthly recorded cases of dengue in the City of Campinas is found at Centro de Vigilância Epidemiológica “Prof. Alexandre Vranjac”(CVE): http://www.cve.saude.sp.gov.br/htm/zoo/dengue_dados.html, publicly available. Daily cases of dengue can be extracted from restricted access site at Sistema de Informação de Agravos de Notificação (http://sinan.saude.gov.br/sinan/login/login.jsf), due to fact that the public availability would compromise patient confidentiality. But, explanation related to permission to access the site can be found at http://sinan.saude.gov.br/sinan/ajuda/ajuda_sinan.pdf?v=1453308019162.
- Number of houses visited aiming the control of breeding sites is found at Superintendencia de Controlo de Endemias (SUCEN): http://200.144.1.23/cdengue, publicly available.

## Results

We present the fittings of the incidence of dengue observed in the City of Campinas applying the four models. The tables that summarize the fittings provide the estimated *β*_*m*_, *β*_*h*_ and *prop*, with the corresponding value of error *Er*, using Eq (18) for the period denoted by [*t*_1_, *t*_2_], and the weighting factors *λ*_1_ and *λ*_2_; the value of the fraction of recovered individuals obtained at the end of the period of estimation *r*_*f*_ = *r*(*t*_2_); and the mean values for the partial and overall effective reproduction numbers ${\bar{R}}_{ef}$, ${\bar{R}}_{ef}^{h}$ and ${\bar{R}}_{ef}^{m}$, using Eq (21).

The four models are fitted against dengue incidence data recorded during four periods in the City of Campinas: Period 1 (1995–2000), Period 2 (2000–2004), Period 3 (2004–2008) and Period 4 (2008–2012); each period begins at month October and ends at September. Hence, *t*_1_ is, for Periods 1, 2, 3 and 4, October 1^*st*^ of respectively years 1995, 2000, 2004 and 2008. And *t*_2_ is September 30 of years 2000, 2004, 2008 and 2012.

### Frequency dependent model—FD

The frequency dependent model, given by Eqs (4), (5) and (6), is considered to fit data from the City of Campinas. The transmission terms are such that only *m*_3_ (density) is multiplied by *D*/*N*. In Table 6 we provide the estimated *β*_*m*_, *β*_*h*_ and *prop*, and other parameters calculated using these estimated values. The value for *λ*_2_ was chosen to the penalty factor be proportional to the sum of the difference between observed and calculated fraction of infectious individuals.

**Table 6:** Model FD: Summary of fitted parameters and epidemiological values for four periods, with *r*_*f*_ = *r*(*t*_2_) and *I*_*d*_ = *i*(*t*_1_)*N*(*t*_1_).

Param.	Period 1	Period 2	Period 3	Period 4
*β*_*m*_	7.597 × 10^−3^	7.104 × 10^−4^	5.42 × 10^−3^	3.919 × 10^−3^
*β*_*h*_	3.105 × 10^−2^	3.9893 × 10^−1^	7.92 × 10^−2^	8.79 × 10^−2^
*prop*	700	150	100	120
*Er*	1.502 × 10^−2^	4.468 × 10^−3^	4.13 × 10^−3^	3.255 × 10^−3^
*λ*_1_	2	2	2	2
*λ*_2_	1 × 10^−5^	1 × 10^−5^	1 × 10^−5^	1 × 10^−5^
*r*_*f*_	2.251 × 10^−1^	8.8 × 10^−2^	1.368 × 10^−1^	1.186 × 10^−1^
${\bar{R}}_{ef}^{m}$	3.299 × 10^−1^	2.8 × 10^−2^	1.66 × 10^−1^	1.407 × 10^−1^
${\bar{R}}_{ef}^{h}$	4.24	60.122	11.291	13.183
${\bar{R}}_{ef}$	2.155	2.440	2.895	2.714
*I*_*d*_	75	25	1	25

The figures corresponding to the four periods are presented separately. For the four periods, we show, using the estimated parameters *β*_*m*_ and *β*_*h*_, the following figures: (1) fraction of cases of dengue multiplied by the term *prop*, that is, *prop* × *cases*(*t*_*j*_) (labelled by O, red), and model based fraction *i* (labelled by E, black); (2) the fraction of infectious mosquitoes *m*_3_/*m*; (3) the partial effective reproduction numbers $R_{ef}^{h}$ (labelled by h, black) and $R_{ef}^{m}$ (labelled by m, red); and (4) the effective reproduction number *R*_*ef*_.

**Fig 7:**
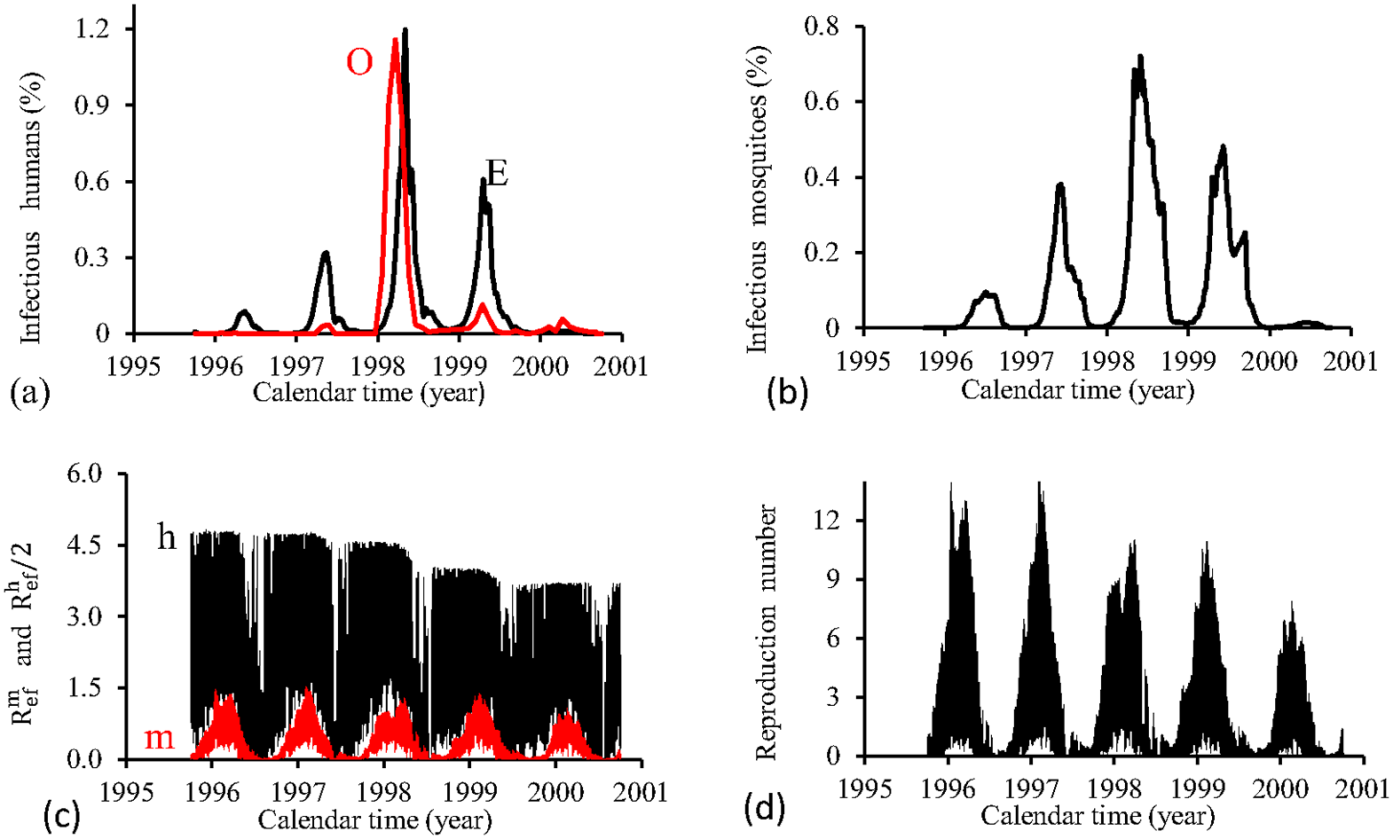
Estimation of Model FD in Period 1 (October/1995—September/2000). (a) observed incidence (O) and adjusted *i* (E), (b) *m*_3_/*m*, (c) $R_{ef}^{h}$ (h) and $R_{ef}^{m}$ (m), and (d) $R_{ef}=R_{ef}^{h}\times R_{ef}^{m}$.

Fig 7 shows the fittings for Period 1 (1995–2000). The third cycle (year 1998) is well adjusted (the fitted curve is slightly delayed), and for other cycles, the fitted epidemics are more severe than real epidemics. The fraction of recovered individuals reaches 22.5% at the end of this period. The maximum peak of the proportion of infectious mosquitoes occurs in year 1998, with value reaching 0.8%. The average partial effective reproduction numbers are ${\bar{R}}_{ef}^{m}=0.33$ and ${\bar{R}}_{ef}^{h}=4.24$, and for the overall number, ${\bar{R}}_{ef}=2.15$.

**Fig 8:**
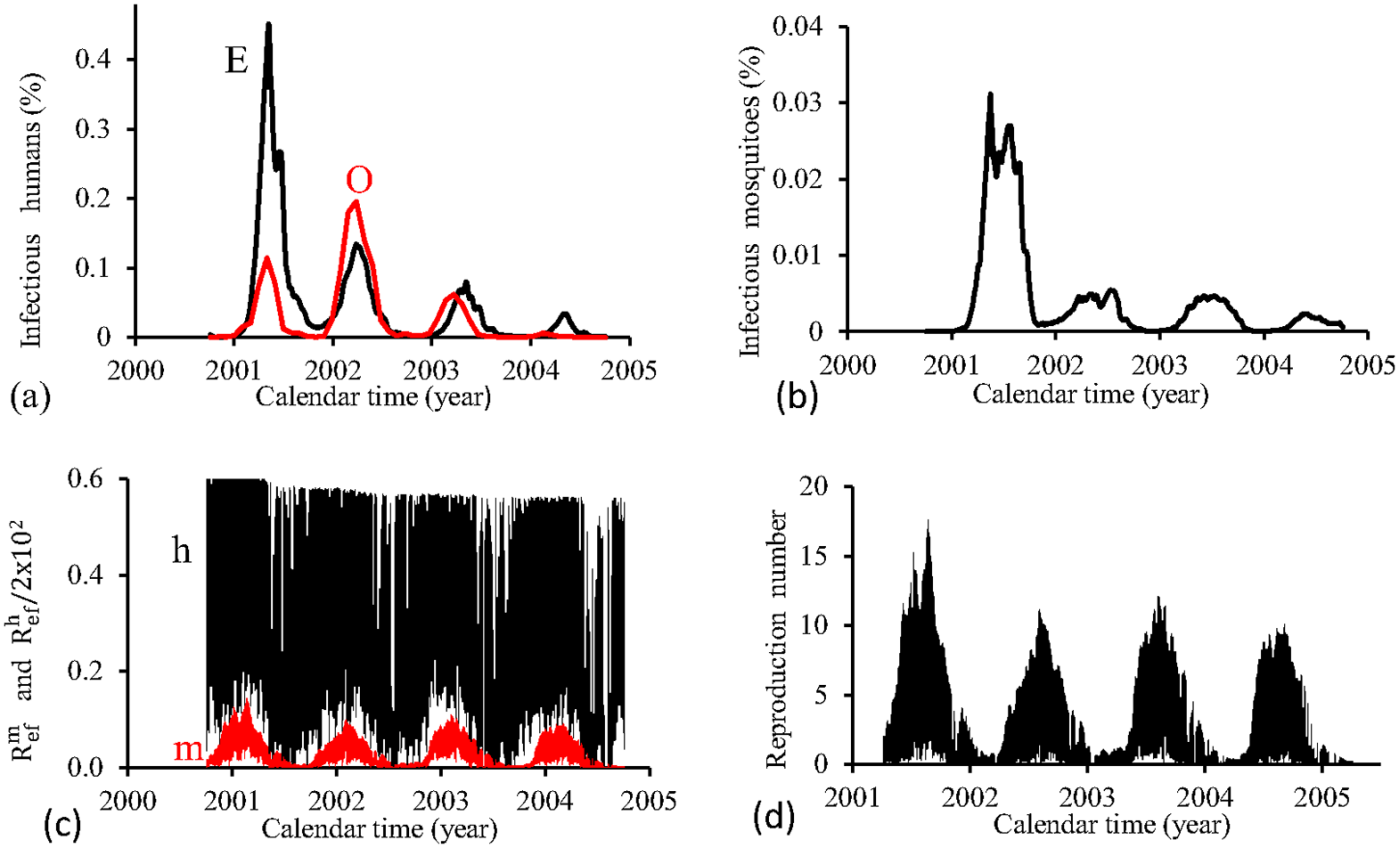
Estimation of Model FD in Period 2 (October/2000—September/2004). (a) observed incidence (O) and adjusted *i* (E), (b) *m*_3_/*m*, (c) $R_{ef}^{h}$ (h) and $R_{ef}^{m}$ (m), and (d) $R_{ef}=R_{ef}^{h}\times R_{ef}^{m}$.

Fig 8 shows the fittings for Period 2 (2000–2004). The second and third cycles (years 2002 and 2003) is reasonably well adjusted, while for other two cycles, the fitted epidemics are more severe than the real epidemics (the first one is very severe). The estimated epidemics curves follow damped oscillations. The fraction of recovered individuals reaches 8.8% at the end of this period. The maximum peak of the proportion of infectious mosquitoes occurs in year 2001, with value reaching 0.03%. The average partial effective reproduction numbers are ${\bar{R}}_{ef}^{m}=0.03$ and ${\bar{R}}_{ef}^{h}=60.1$, and for the overall number, ${\bar{R}}_{ef}=2.44$.

**Fig 9:**
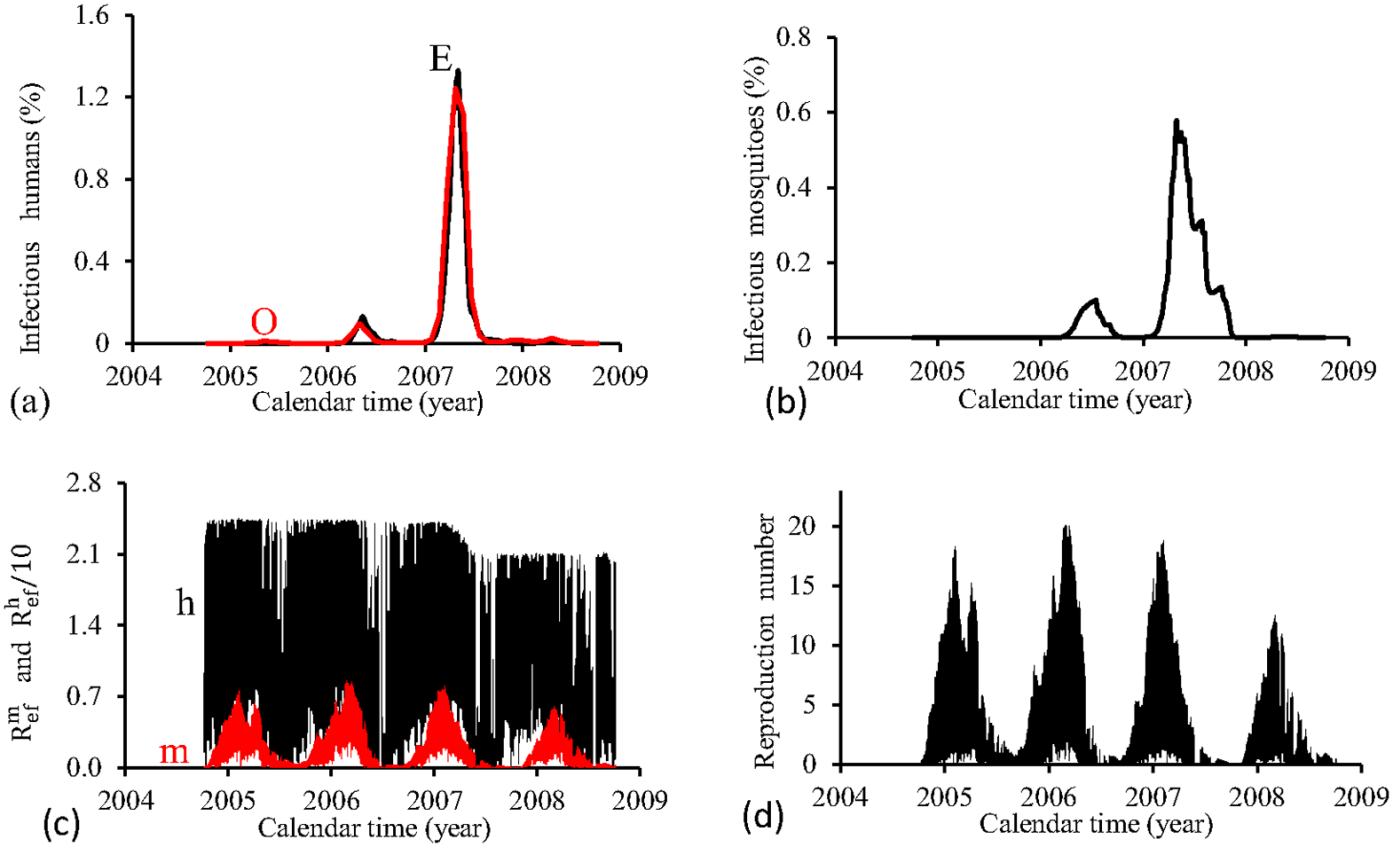
Estimation of Model FD in Period 3 (October/2004—September/2008). (a) observed incidence (O) and adjusted *i* (E), (b) *m*_3_/*m*, (c) $R_{ef}^{h}$ (h) and $R_{ef}^{m}$ (m), and (d) $R_{ef}=R_{ef}^{h}\times R_{ef}^{m}$.

Fig 9 shows the fittings for Period 3 (2004–2008). All epidemics cycles are well adjusted. The fraction of recovered individuals reaches 13.7% at the end of this period. The maximum peak of the proportion of infectious mosquitoes occurs in year 2007, with value reaching 0.6%. The average partial effective reproduction numbers are ${\bar{R}}_{ef}^{m}=0.17$ and ${\bar{R}}_{ef}^{h}=11.3$, and for the overall number, ${\bar{R}}_{ef}=2.90$.

**Fig 10:**
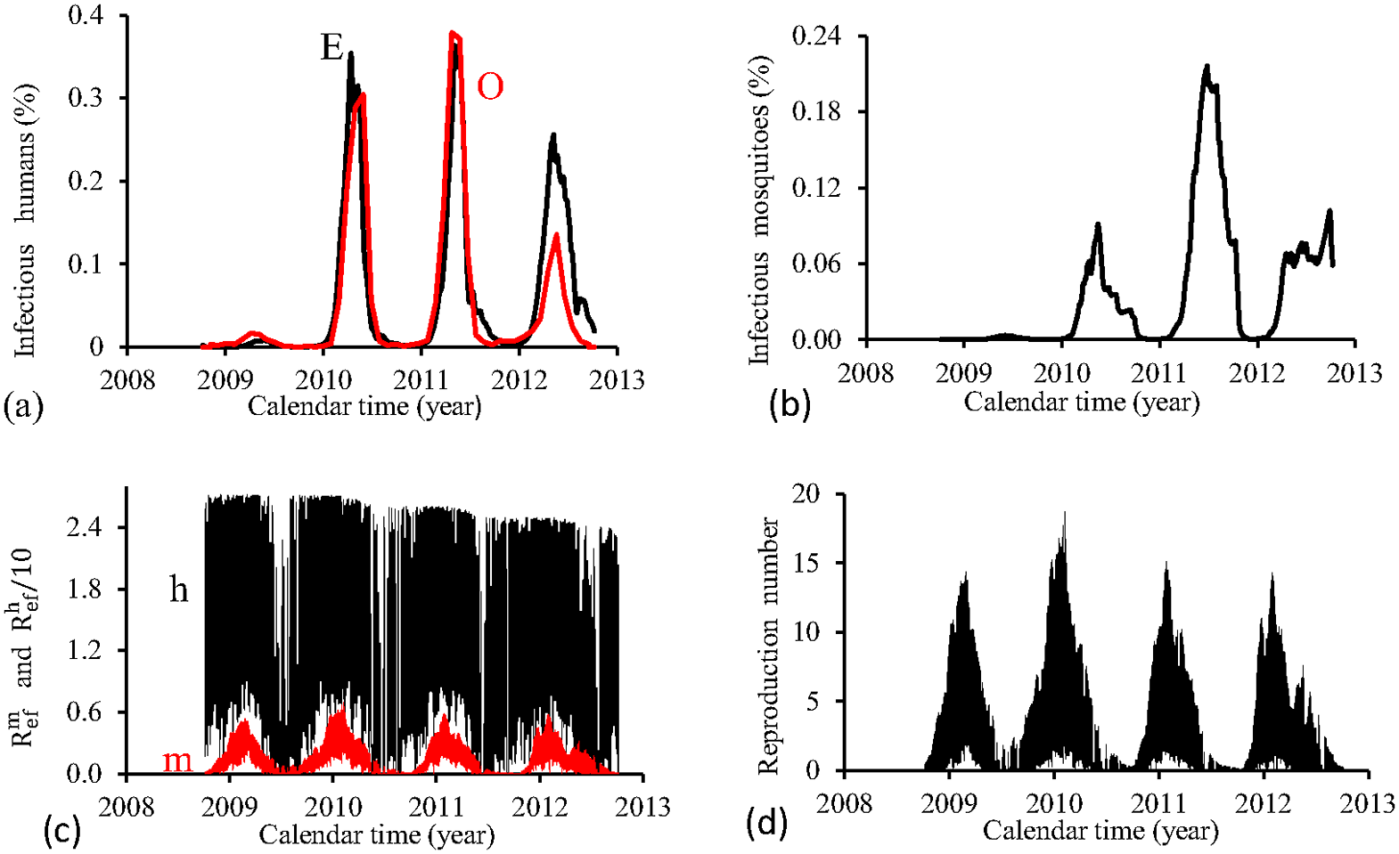
Estimation of Model FD in Period 4 (October/2008—September/2012). (a) observed incidence (O) and adjusted *i* (E), (b) *m*_3_/*m*, (c) $R_{ef}^{h}$ (h) and $R_{ef}^{m}$ (m), and (d) $R_{ef}=R_{ef}^{h}\times R_{ef}^{m}$.

Fig 10 shows the fittings for Period 4 (2008–2012). All epidemics cycles are well adjusted, except the last cycle, when the estimated epidemics is more severe than the real one. The fraction of recovered individuals reaches 11.9% at the end of this period. The maximum peak of the proportion of infectious mosquitoes occurs in year 2011, with value reaching 0.24%. The average partial effective reproduction numbers are ${\bar{R}}_{ef}^{m}=0.14$ and ${\bar{R}}_{ef}^{h}=13.2$, and for the overall number, ${\bar{R}}_{ef}=2.71$.

The FD model fitted better the third and fourth periods, while in the first and second periods, only one and two, respectively, of the epidemics peaks are well adjusted. In the second period, the order of magnitude between peaks of $R_{ef}^{h}$ and $R_{ef}^{m}$ situates at 10^2^, while for other three periods, the difference was 10. Notice that the mean partial numbers follow these order of magnitude. The fraction of recovered individuals reached around 10%, except for period 1, when reached 22%. Comparing fittings from periods 1 (the lowest ${\bar{R}}_{ef}$) and 3 (the highest ${\bar{R}}_{ef}$), we observe that: (1) a prominent epidemics in the third cycle, (2) higher peak of infectious mosquitoes and *prop*, but lower ${\bar{R}}_{ef}^{h}$, in the period 1 than period 3, and (3) period 1 presented the highest error *E*_*r*_ and initial infectious individuals *I*_*d*_.

The FD model showed a plateau in curves for $R_{ef}^{h}$, but triangular shape for $R_{ef}^{m}$ curves. Triangle shaped curve is also presented by the overall *R*_*ef*_. These findings show strong dependency of infective term among mosquito population with abiotic variations, but quite insensitive among humans. However, the strong seasonal dependency of mosquito population prevails in the overall transmission of dengue.

### Pseudo mass action law model—PMAL

The pseudo mass action law model, given by Eqs (4), (5) and (7), is considered to fit data from the City of Campinas. The transmission terms are such that *i* and *m*_3_ (density) are multiplied by *N* and *D*, respectively. In Table 7 we provide the estimated *β*_*m*_, *β*_*h*_ and *prop*, and other parameters calculated using these estimated values.

**Table 7:** Model PMAL: Summary of fitted parameters and epidemiological values for four periods, with *r*_*f*_ = *r*(*t*_2_) and *I*_*d*_ = *i*(*t*_1_)*N*(*t*_1_).

Param.	Period 1	Period 2	Period 3	Period 4
*β*_*m*_	3.082 × 10^−9^	4.130 × 10^−9^	2.965 × 10^−9^	3.907 × 10^−9^
*β*_*h*_	9.17 × 10^−8^	8.530 × 10^−8^	1.364 × 10^−7^	6.934 × 10^−8^
*prop*	739.99	549.845	102.199	224.347
*Er*	1.85 × 10^−2^	9.596 × 10^−3^	3.898 × 10^−3^	5.428 × 10^−3^
*λ*_1_	2	2	2	2
*λ*_2_	1 × 10^−5^	1 × 10^−5^	1 × 10^−5^	1 × 10^−5^
*r*_*f*_	2.383 × 10^−1^	2.686 × 10^−1^	1.411 × 10^−1^	1.715 × 10^−1^
${\bar{R}}_{ef}^{m}$	1.241 × 10^−1^	1.597 × 10^−1^	9.379 × 10^−2^	1.557 × 10^−1^
${\bar{R}}_{ef}^{h}$	11.715	11.590	20.129	10.929
${\bar{R}}_{ef}$	2.234	2.717	2.916	2.497
*I*_*d*_	50	18	1	200

The figures corresponding to the four periods are presented separately. For the four periods, we show, using the estimated parameters *β*_*m*_ and *β*_*h*_, the following figures: (1) fraction of cases of dengue multiplied by the term *prop*, that is, *prop* × *cases*(*t*_*j*_) (labelled by O, red), and model based fraction *i* (labelled by E, black); (2) the fraction of infectious mosquitoes *m*_3_/*m*; (3) the partial effective reproduction numbers $R_{ef}^{h}$ (labelled by h, black) and $R_{ef}^{m}$ (labelled by m, red); and (4) the effective reproduction number *R*_*ef*_.

**Fig 11:**
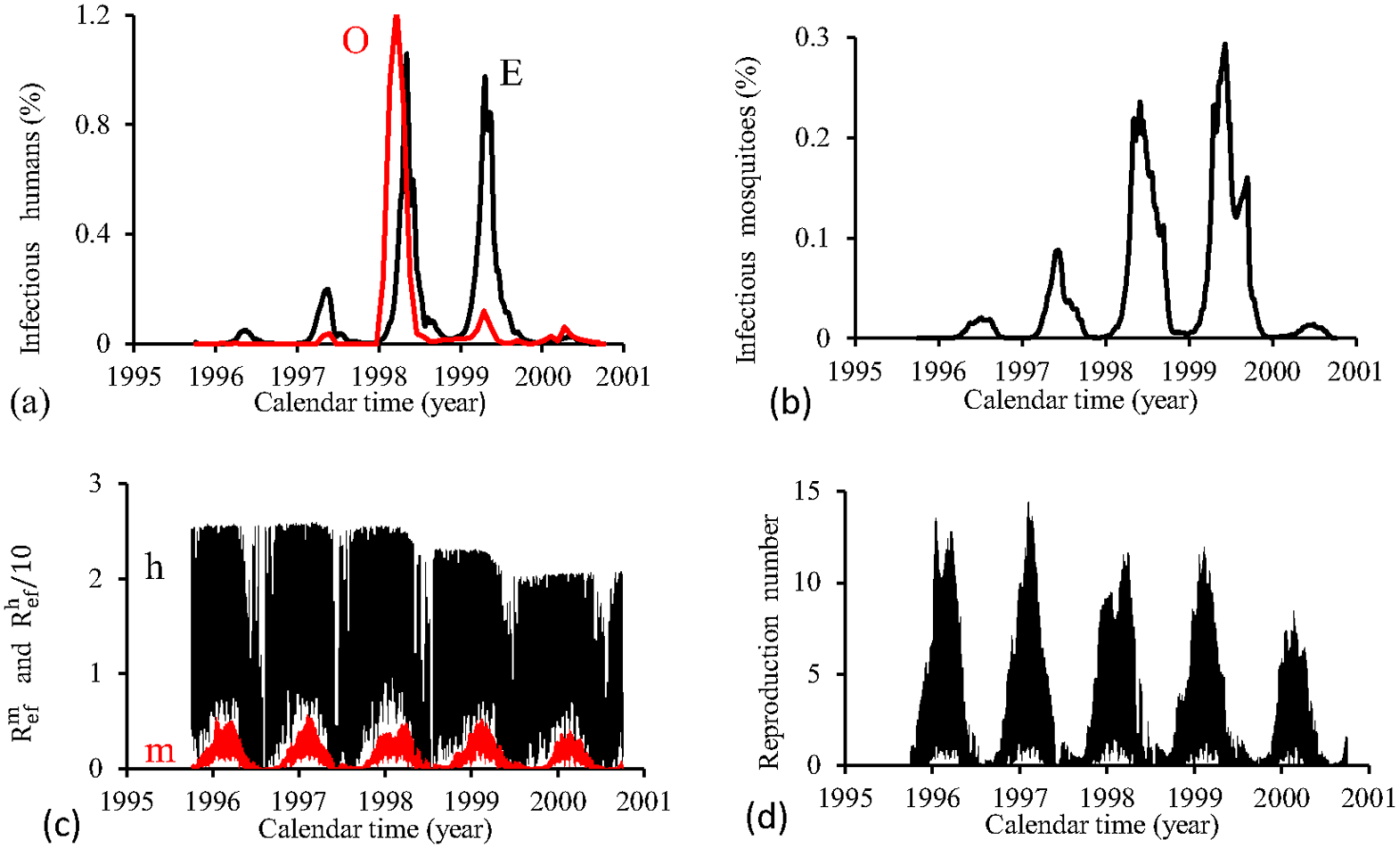
Estimation of Model PMAL in Period 1 (October/1995—September/2000). (a) observed incidence (O) and adjusted *i* (E), (b) *m*_3_/*m*, (c) $R_{ef}^{h}$ (h) and $R_{ef}^{m}$ (m), and (d) $R_{ef}=R_{ef}^{h}\times R_{ef}^{m}$.

Fig 11 shows the fittings for Period 1 (1995–2000). The fitting of incidence data is quite similar than that obtained for FD model. The fraction of recovered individuals reaches 23.8% at the end of this period. The maximum peak of the proportion of infectious mosquitoes occurs in year 1999, with value reaching 0.3% (in FD model occurred at 1998, with peak around 2.5-fold higher). The average partial effective reproduction numbers are ${\bar{R}}_{ef}^{m}=0.12$ and ${\bar{R}}_{ef}^{h}=11.7$, and for the overall number, ${\bar{R}}_{ef}=2.23$.

**Fig 12:**
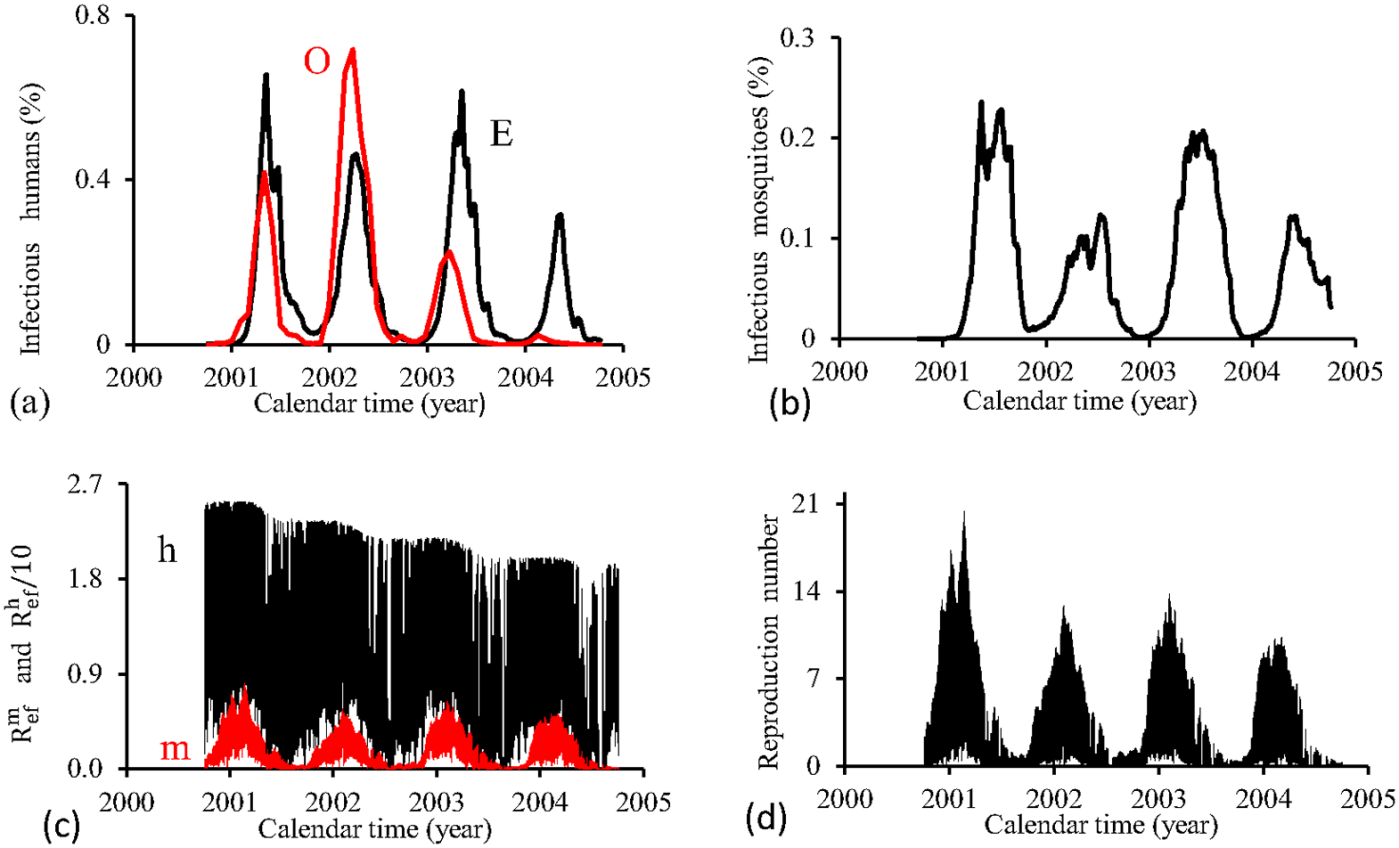
Estimation of Model PMAL in Period 2 (October/2000—September/2004). (a) observed incidence (O) and adjusted *i* (E), (b) *m*_3_/*m*, (c) $R_{ef}^{h}$ (h) and $R_{ef}^{m}$ (m), and (d) $R_{ef}=R_{ef}^{h}\times R_{ef}^{m}$.

Fig 12 shows the fittings for Period 2 (2000–2004). Instead of the damped oscillations obtained for FD model, there are irregular oscillations. The second epidemics curve separates two more severe epidemics instead of being less severe as in the observed data. The fraction of recovered individuals reaches 26.9% at the end of this period. The maximum peak of the proportion of infectious mosquitoes occurs in year 2001, with value reaching 0.25%, which is near the third peak. The average partial effective reproduction numbers are ${\bar{R}}_{ef}^{m}=0.16$ and ${\bar{R}}_{ef}^{h}=11.6$, and for the overall number, ${\bar{R}}_{ef}=2.72$.

**Fig 13:**
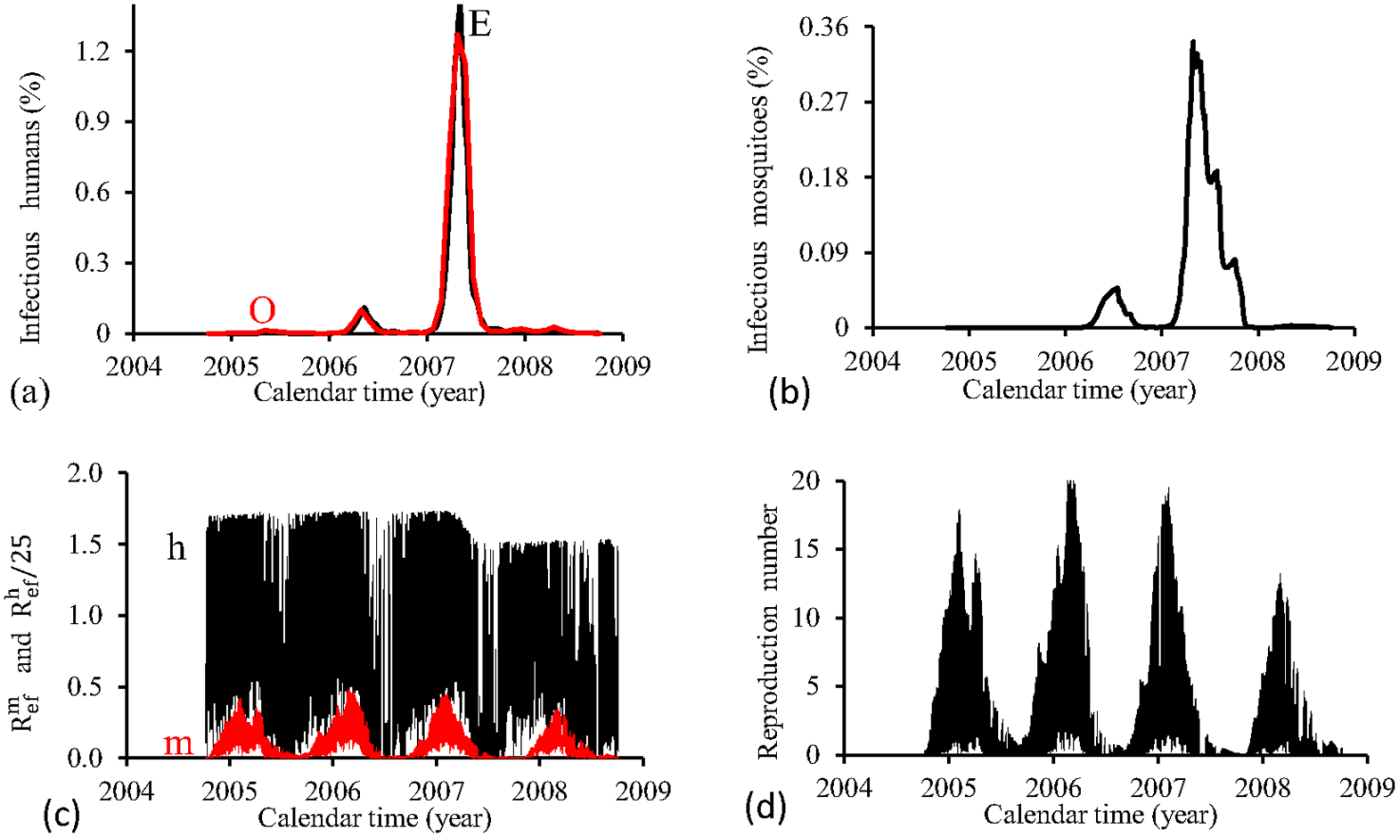
Estimation of Model PMAL in Period 3 (October/2004—September/2008). (a) observed incidence (O) and adjusted *i* (E), (b) *m*_3_/*m*, (c) $R_{ef}^{h}$ (h) and $R_{ef}^{m}$ (m), and (d) $R_{ef}=R_{ef}^{h}\times R_{ef}^{m}$.

Fig 13 shows the fittings for Period 3 (2004–2008). The fitting of incidence data is quite similar than that obtained for FD model. The fraction of recovered individuals reaches 14.1% at the end of this period. The maximum peak of the proportion of infectious mosquitoes occurs in year 2007, with value reaching 0.37%. The average partial effective reproduction numbers are ${\bar{R}}_{ef}^{m}=0.09$ and ${\bar{R}}_{ef}^{h}=20.1$, and for the overall number, ${\bar{R}}_{ef}=2.92$.

**Fig 14:**
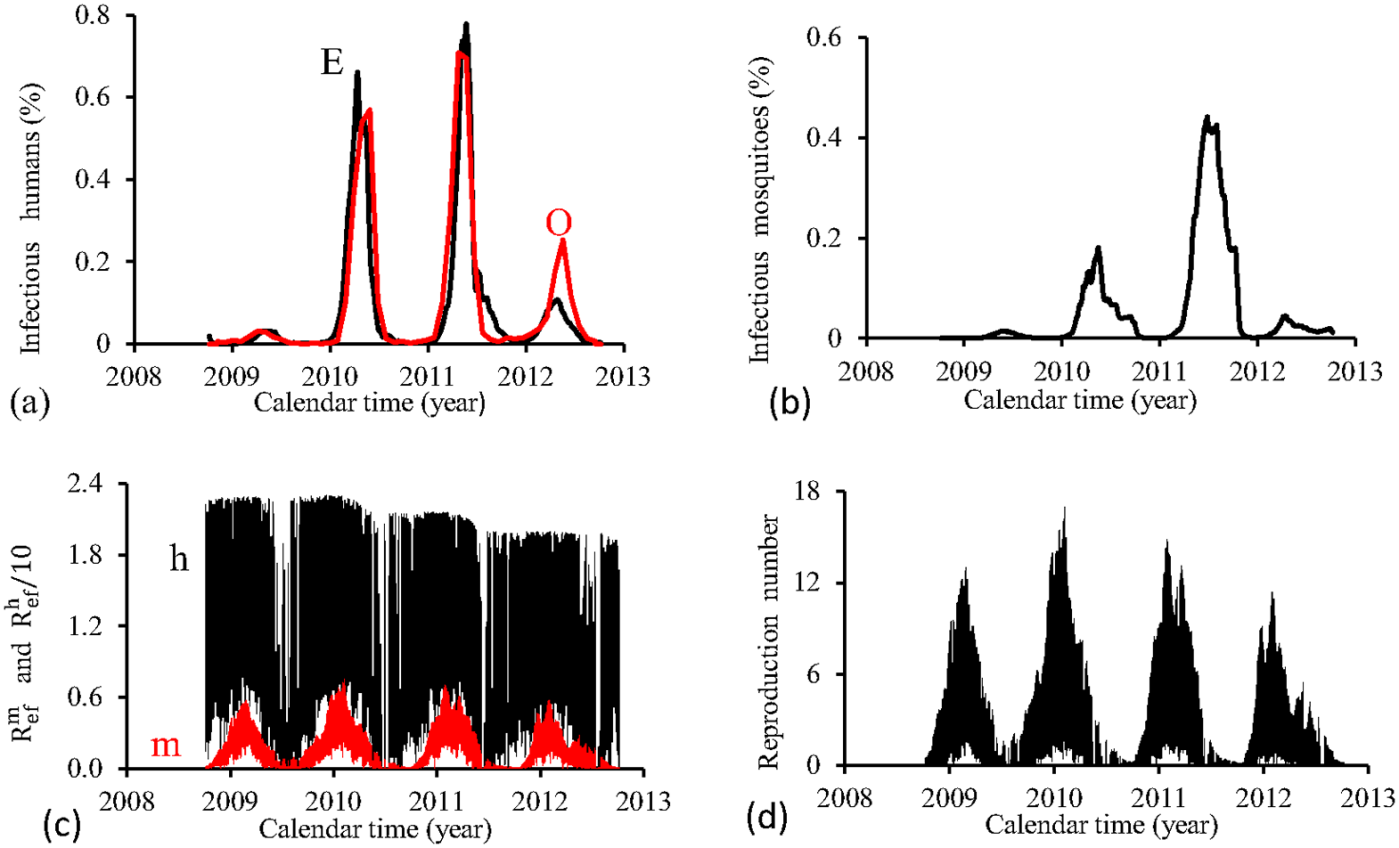
Estimation of Model PMAL in Period 4 (October/2008—September/2012). (a) observed incidence (O) and adjusted *i* (E), (b) *m*_3_/*m*, (c) $R_{ef}^{h}$ (h) and $R_{ef}^{m}$ (m), and (d) $R_{ef}=R_{ef}^{h}\times R_{ef}^{m}$.

Fig 14 shows the fittings for Period 4 (2008–2012). The first three epidemics cycles are well adjusted, except the last cycle, which is mild (in opposition to FD model). The fraction of recovered individuals reaches 17.2% at the end of this period. The maximum peak of the proportion of infectious mosquitoes occurs in year 2011, with value reaching 0.47%. The average partial effective reproduction numbers are ${\bar{R}}_{ef}^{m}=0.16$ and ${\bar{R}}_{ef}^{h}=10.9$, and for the overall number, ${\bar{R}}_{ef}=2.50$.

The PMAL model fitted better the third and fourth periods, while in the first and second periods, only one and two, respectively, of the epidemics peaks are well adjusted. In all periods, the order of magnitude between peaks of $R_{ef}^{h}$ and $R_{ef}^{m}$ situates at 10^2^. The fraction of recovered individuals reached around 25% (periods 1 and 2) and 15% (periods 3 and 4). Comparing fittings form periods 1 (the lowest ${\bar{R}}_{ef}$) and 3 (the highest ${\bar{R}}_{ef}$), the results are similar than that observed in FD model.

Both PMAL and FD models presented quite the same pattern of fittings for all periods. Also, both PMAL and FD models showed same characteristics with respect to effective reproduction numbers.

### True mass action law model—TMAL

The true mass action law model, given by Eqs (4), (5) and (8), is considered to fit data from the City of Campinas. The transmission terms *i* and *m*_3_/*m* (fraction) appear alone. In Table 8 we provide the estimated *β*_*m*_, *β*_*h*_ and *prop*, and other parameters calculated using these estimated values.

**Table 8:** Model TMAL: Summary of fitted parameters and epidemiological values for four periods, with *r*_*f*_ = *r*(*t*_2_) and *I*_*d*_ = *i*(*t*_1_)*N*(*t*_1_).

Param.	Period 1	Period 2	Period 3	Period 4
*β*_*m*_	1.7 × 10^−2^	8.145 × 10^−4^	2.333 × 10^−2^	4.733 × 10^−3^
*β*_*h*_	2.3 × 10^−2^	5.979 × 10^−1^	2.12 × 10^−2^	1.214 × 10^−1^
*prop*	862.38	450	294.44	446.24
*Er*	2.052 × 10^−2^	2.037 × 10^−2^	2.185 × 10^−3^	2.965 × 10^−2^
*λ*_1_	2	2	2	2
*λ*_2_	1 × 10^−5^	1 × 10^−5^	1 × 10^−5^	1 × 10^−5^
*r*_*f*_	5.125 × 10^−1^	5.49 × 10^−1^	6.931 × 10^−1^	8.425 × 10^−1^
${\bar{R}}_{ef}^{m}$	4.372 × 10^−1^	2.223 × 10^−2^	5.89 × 10^−1^	1.281 × 10^−1^
${\bar{R}}_{ef}^{h}$	2.642	65.59	2.418	11.642
${\bar{R}}_{ef}$	1.69	2.07	2.12	2.12
*I*_*d*_	50	55	5	15

The figures corresponding to the four periods are presented separately. For the four periods, we show, using the estimated parameters *β*_*m*_ and *β*_*h*_, the following figures: (1) fraction of cases of dengue multiplied by the term *prop*, that is, *prop* × *cases*(*t*_*j*_) (labelled by O, red), and model based fraction *i* (labelled by E, black); (2) the fraction of infectious mosquitoes *m*_3_/*m*; (3) the partial effective reproduction numbers $R_{ef}^{h}$ (labelled by h, black) and $R_{ef}^{m}$ (labelled by m, red); and (4) the effective reproduction number *R*_*ef*_.

**Fig 15:**
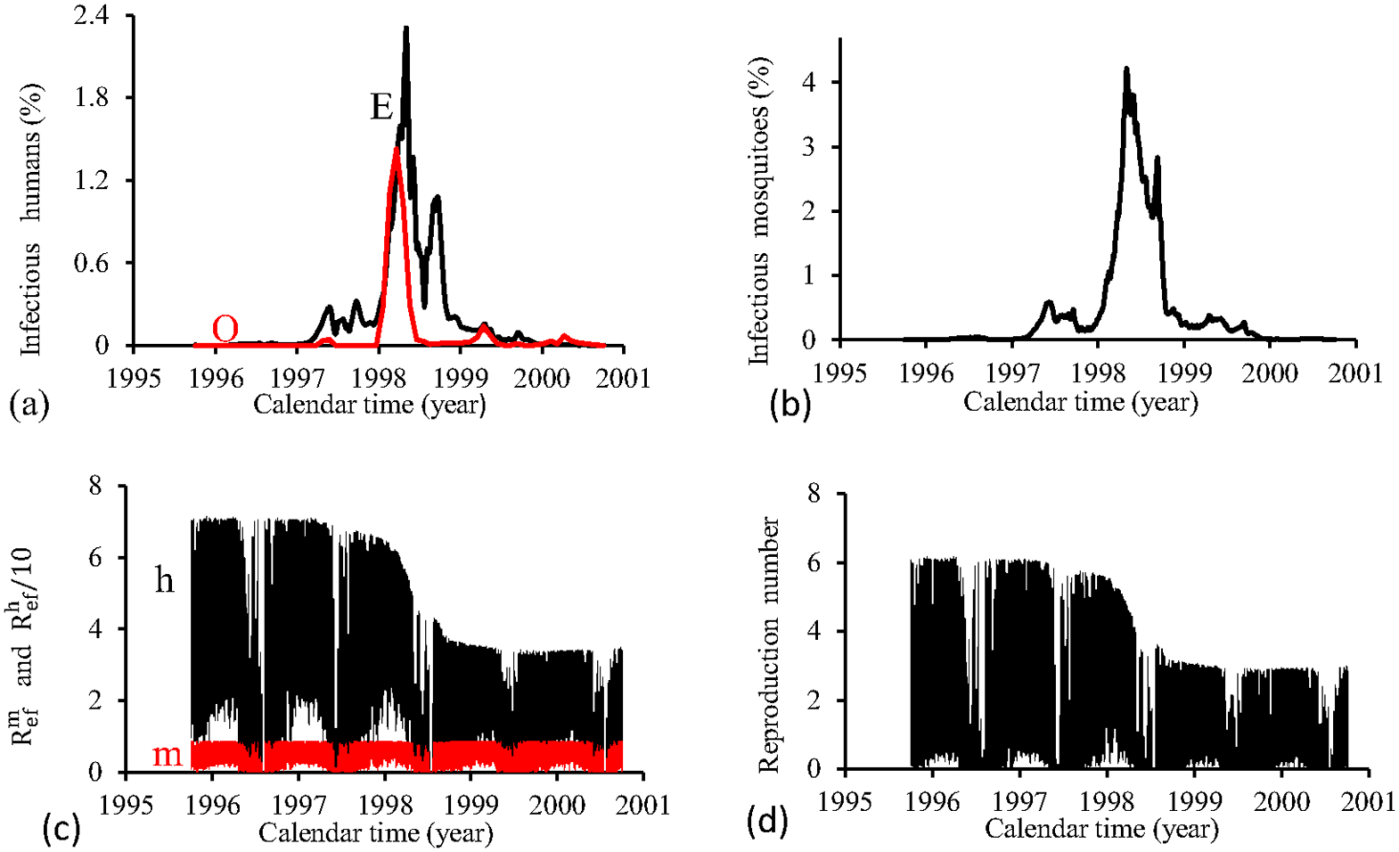
Estimation of Model TMAL in Period 1 (October/1995—September/2000). (a) observed incidence (O) and adjusted *i* (E), (b) *m*_3_/*m*, (c) $R_{ef}^{h}$ (h) and $R_{ef}^{m}$ (m), and (d) $R_{ef}=R_{ef}^{h}\times R_{ef}^{m}$.

Fig 15 shows the fittings for Period 1 (1995–2000). The fitting is poor, with the most severe epidemics overlapping the next epidemics. All estimated epidemics cycles are severe. The fraction of recovered individuals reaches 51.3% at the end of this period. The maximum peak of the proportion of infectious mosquitoes occurs in year 1998, with value reaching 4.2%. The average partial effective reproduction numbers are ${\bar{R}}_{ef}^{m}=0.4$ and ${\bar{R}}_{ef}^{h}=2.64$, and for the overall number, ${\bar{R}}_{ef}=1.69$.

**Fig 16:**
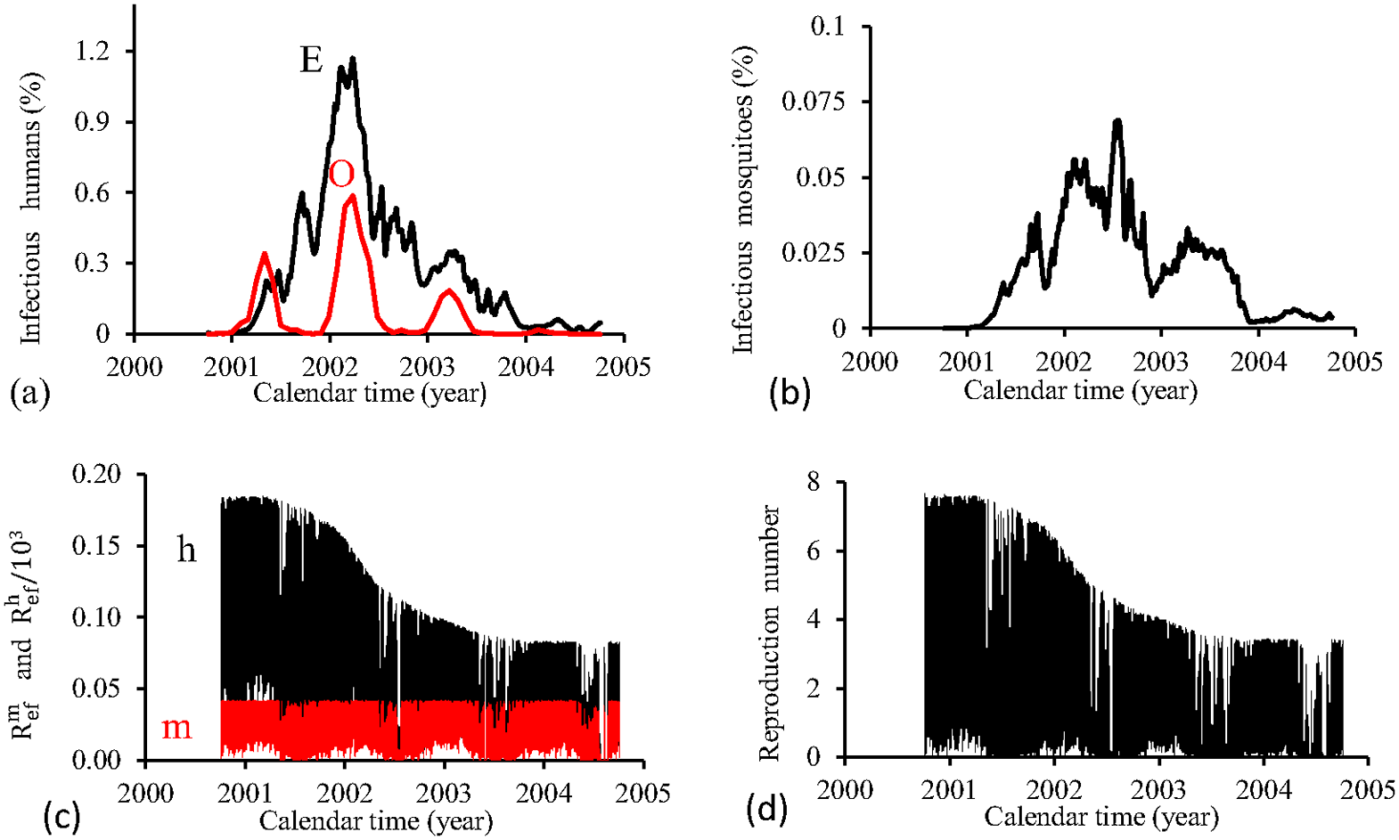
Estimation of Model TMAL in Period 2 (October/2000—September/2004). (a) observed incidence (O) and adjusted *i* (E), (b) *m*_3_/*m*, (c) $R_{ef}^{h}$ (h) and $R_{ef}^{m}$ (m), and (d) $R_{ef}=R_{ef}^{h}\times R_{ef}^{m}$.

Fig 16 shows the fittings for Period 2 (2000–2004). The fitting is very poor, with the most severe epidemics overlapping the next epidemics. All estimated epidemics cycles are severe. The fraction of recovered individuals reaches 54.9% at the end of this period. The maximum peak of the proportion of infectious mosquitoes occurs in year 2002, with value reaching 0.08%. The average partial effective reproduction numbers are ${\bar{R}}_{ef}^{m}=0.02$ and ${\bar{R}}_{ef}^{h}=65.6$, and for the overall number, ${\bar{R}}_{ef}=2.07$.

**Fig 17:**
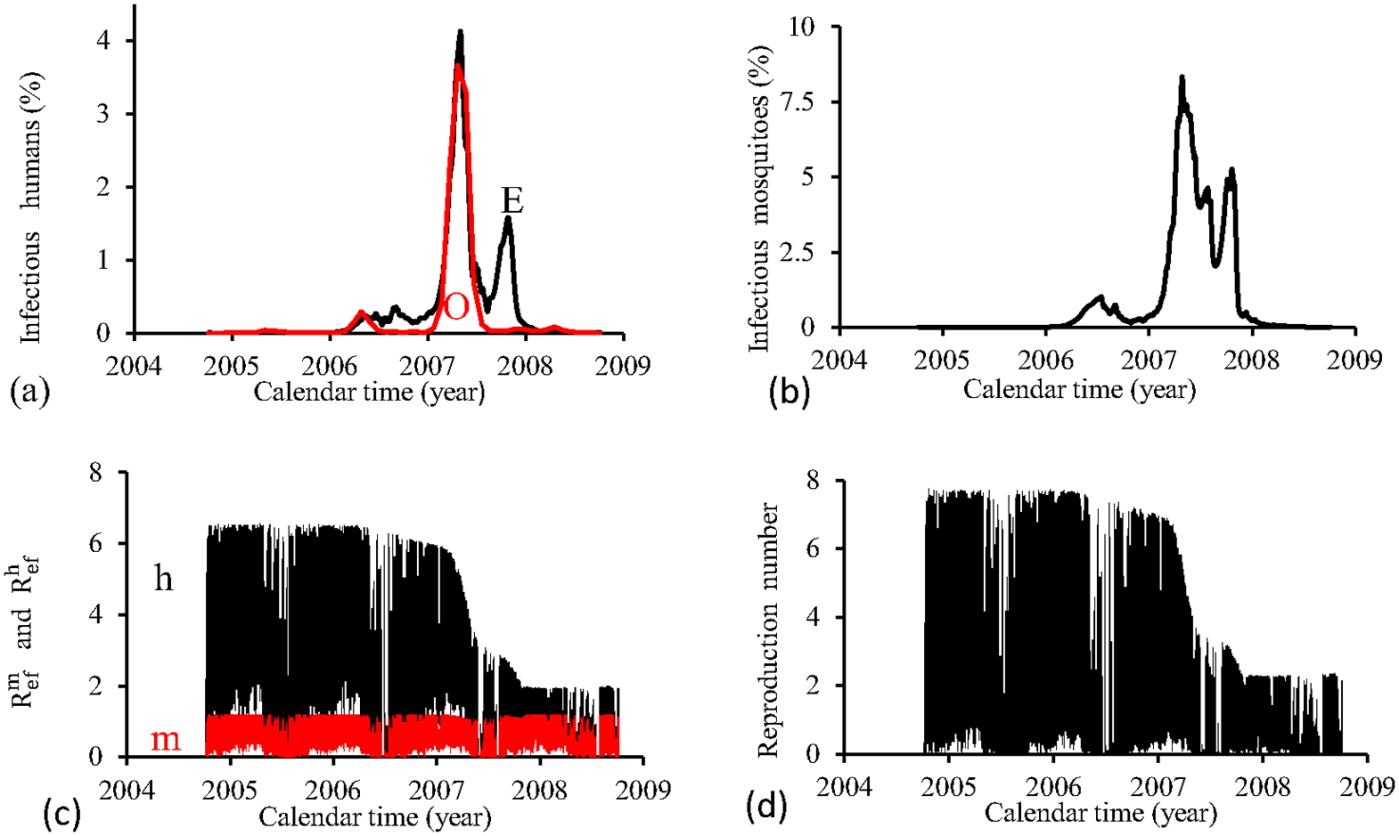
Estimation of Model TMAL in Period 3 (October/2004—September/2008). (a) observed incidence (O) and adjusted *i* (E), (b) *m*_3_/*m*, (c) $R_{ef}^{h}$ (h) and $R_{ef}^{m}$ (m), and (d) $R_{ef}=R_{ef}^{h}\times R_{ef}^{m}$.

Fig 17 shows the fittings for Period 3 (2004–2008). The fitting is reasonable, with the most severe epidemics overlapping the next epidemics. Estimated epidemics cycles are severe. The fraction of recovered individuals reaches 69.3% at the end of this period. The maximum peak of the proportion of infectious mosquitoes occurs in year 2007, with value reaching 8%. The average partial effective reproduction numbers are ${\bar{R}}_{ef}^{m}=0.59$ and ${\bar{R}}_{ef}^{h}=2.42$, and for the overall number, ${\bar{R}}_{ef}=2.12$.

**Fig 18:**
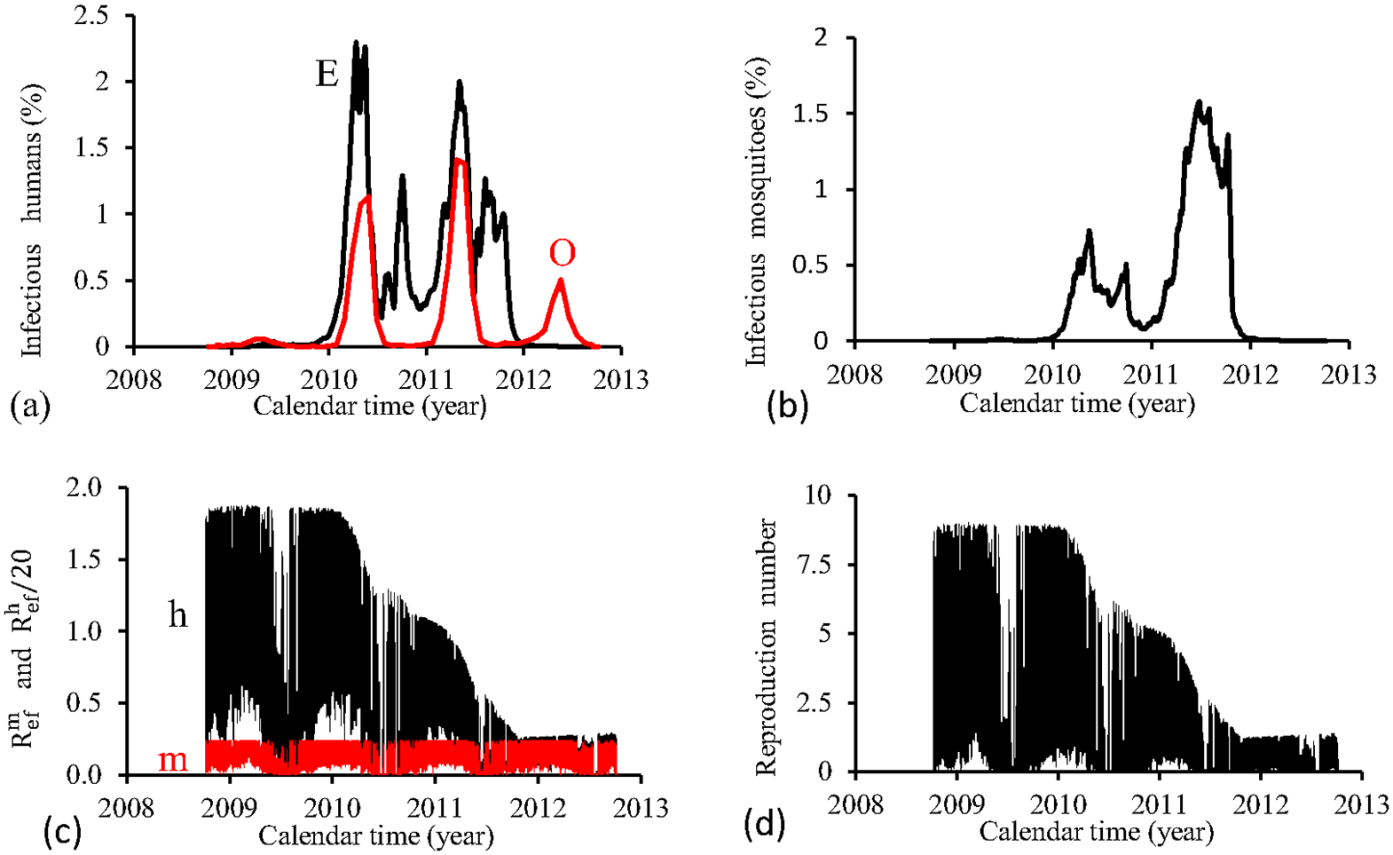
Estimation of Model TMAL in Period 4 (October/2008—September/2012). (a) observed incidence (O) and adjusted *i* (E), (b) *m*_3_/*m*, (c) $R_{ef}^{h}$ (h) and $R_{ef}^{m}$ (m), and (d) $R_{ef}=R_{ef}^{h}\times R_{ef}^{m}$.

Fig 18 shows the fittings for Period 4 (2008–2012). Two epidemics cycles are reasonably localized, while the fourth cycle is absent. All epidemics are overlapped. There are epidemics in the winter of the years 2010 and 2011. The fraction of recovered individuals reaches 84.3% at the end of this period. The maximum peak of the proportion of infectious mosquitoes occurs in the year 2011 reaching 1.6%. The average partial effective reproduction numbers are ${\bar{R}}_{ef}^{m}=0.13$ and ${\bar{R}}_{ef}^{h}=11.6$, and for the overall number, ${\bar{R}}_{ef}=2.12$.

The TMAL model fitted reasonably the third period. The epidemics overlap each other, eliminating the inter-epidemics observed in previous FD and PMAL models. Other feature is the increasing phase of epidemics occurs during winter (middle of the year) in some epidemics cycles. In the first and fourth periods, the quotient of the magnitude of the peaks of $R_{ef}^{h}$ and $R_{ef}^{m}$ is both of order 10^2^, while for second and third periods, the order of magnitudes were 10^3^ and 10, respectively. Notice that the mean of the partial numbers (${\bar{R}}_{ef}^{h}$ and ${\bar{R}}_{ef}^{m}$) follow these order of magnitudes. The fraction of recovered individuals reached between 50% and 80%. Comparing fittings form periods 1 (the lowest ${\bar{R}}_{ef}$) and 3 (the highest ${\bar{R}}_{ef}$), we observe that: (1) a prominent epidemics in the third cycle, (2) lower peak of infectious mosquitoes and lower ${\bar{R}}_{ef}^{h}$, but higher *prop*, in the period 1 than period 3, and (3) period 1 presented the highest error *E*_*r*_ and initial infectious individuals *I*_*d*_.

The TMAL model showed a plateau in both curves for $R_{ef}^{h}$ and $R_{ef}^{m}$ curves. This shape is also presented by the overall *R*_*ef*_. These findings show quite insensitive dengue transmission among humans and mosquitoes with respect to abiotic variations. However, there are measurable decreasing in the partial effective number in humans $R_{ef}^{h}$ and the overall effective number *R*_*ef*_ after epidemics. This decreasing is much more pronounced than in the previous two models.

### Special incidence rate model—SIR

The special incidence rate model, given by Eqs (4), (5) and (9), is considered to fit data from the City of Campinas. The transmission terms are such that *i* and *m*_3_/*m* (fraction) are divided by *Dm* and *N*, respectively. In Table 9, we provide the estimated *β*_*m*_, *β*_*h*_ and *prop*, and other parameters calculated using these estimated values.

**Table 9:** Model SIR: Summary of fitted parameters and epidemiological values for four periods, with *r*_*f*_ = *r*(*t*_2_) and *I*_*d*_ = *i*(*t*_1_)*N*(*t*_1_).

Param.	Period 1	Period 2	Period 3	Period 4
*β*_*m*_	1.370 × 10^3^	2.52 × 10^3^	1.235 × 10^4^	1.505 × 10^4^
*β*_*h*_	2.16 × 10^5^	1.971 × 10^5^	1.555 × 10^4^	5.694 × 10^4^
*prop*	519.849	400	249	524.97
*Er*	2.230 × 10^−2^	5.732 × 10^−2^	5.963 × 10^−2^	5.957 × 10^−2^
*λ*_1_	2	2	2	2
*λ*_2_	1 × 10^−5^	1 × 10^−5^	1 × 10^−5^	1 × 10^−5^
*r*_*f*_	3.962 × 10^−1^	7.853 × 10^−1^	2.943 × 10^−1^	4.472 × 10^−1^
${\bar{R}}_{ef}^{m}$	3.810 × 10^−2^	7.018 × 10^−2^	3.028 × 10^−1^	3.71 × 10^−1^
${\bar{R}}_{ef}^{h}$	52.613	29.346	9.61	7.4
${\bar{R}}_{ef}$	2.734	2.795	4.21	3.737
*I*_*d*_	250	35	500	500

The figures corresponding to the four periods are presented separately. For the four periods, we show, using the estimated parameters *β*_*m*_ and *β*_*h*_, the following figures: (1) fraction of cases of dengue multiplied by the term *prop*, that is, *prop* × *cases*(*t*_*j*_) (labelled by O, red), and model based fraction *i* (labelled by E, black); (2) the fraction of infectious mosquitoes *m*_3_/*m*; (3) the partial effective reproduction numbers $R_{ef}^{h}$ (labelled by h, black) and $R_{ef}^{m}$ (labelled by m, red); and (4) the effective reproduction number *R*_*ef*_.

**Fig 19:**
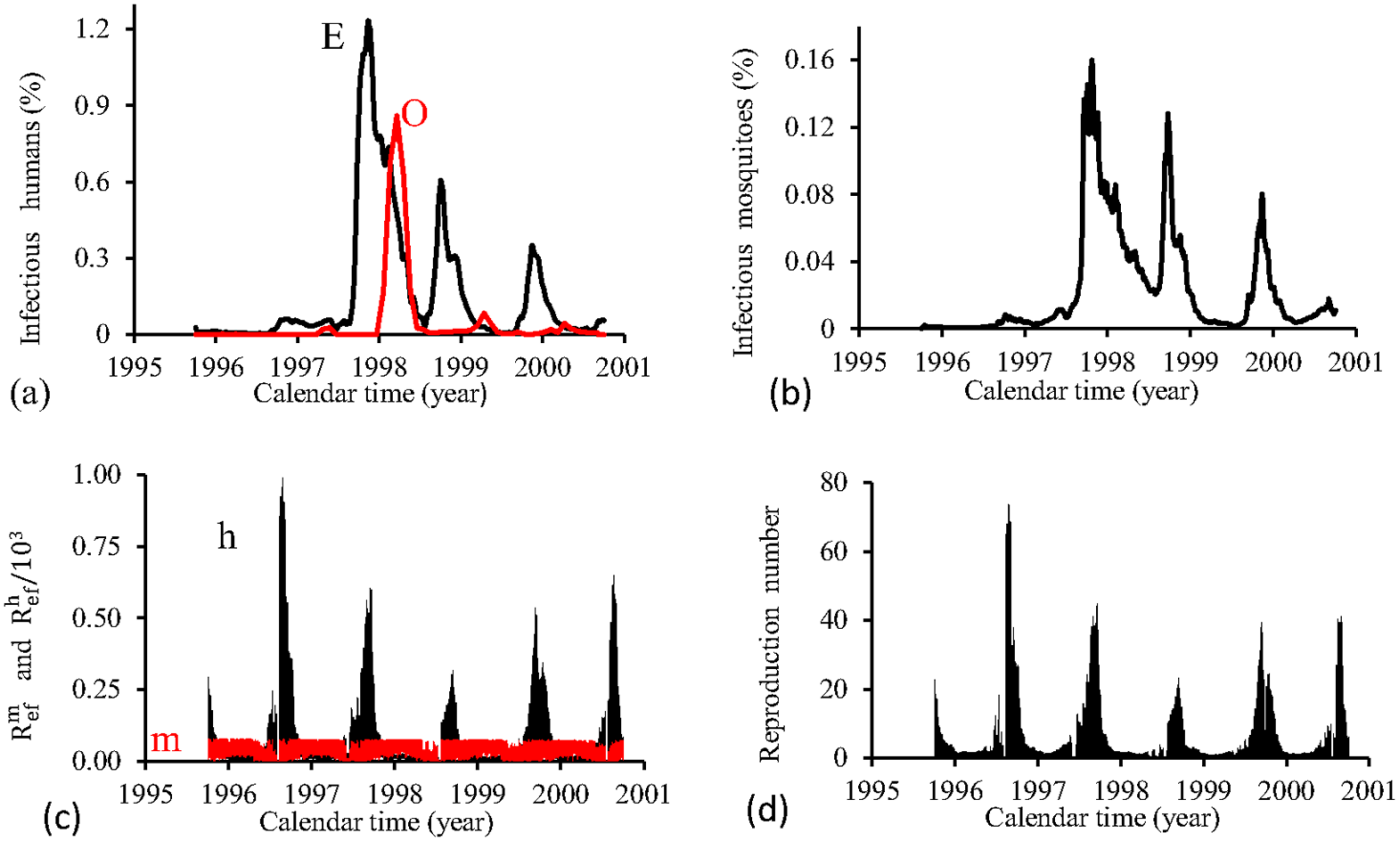
Estimation of Model SIR in Period 1 (October/1995—September/2000). (a) observed incidence (O) and adjusted *i* (E), (b) *m*_3_/*m*, (c) $R_{ef}^{h}$ (h) and $R_{ef}^{m}$ (m), and (d) $R_{ef}=R_{ef}^{h}\times R_{ef}^{m}$.

Fig 19 shows the fittings for Period 1 (1995–2000). The fitting is poor, with the most severe epidemics overleaping the next epidemics. All estimated epidemics cycles are severe. The fraction of recovered individuals reaches 39.6% at the end of this period. The maximum peak of the proportion of infectious mosquitoes occurs between years 1997 and 1998, with value reaching 0.16%. The average partial effective reproduction numbers are ${\bar{R}}_{ef}^{m}=0.04$ and ${\bar{R}}_{ef}^{h}=52.6$, and for the overall number, ${\bar{R}}_{ef}=2.73$.

**Fig 20:**
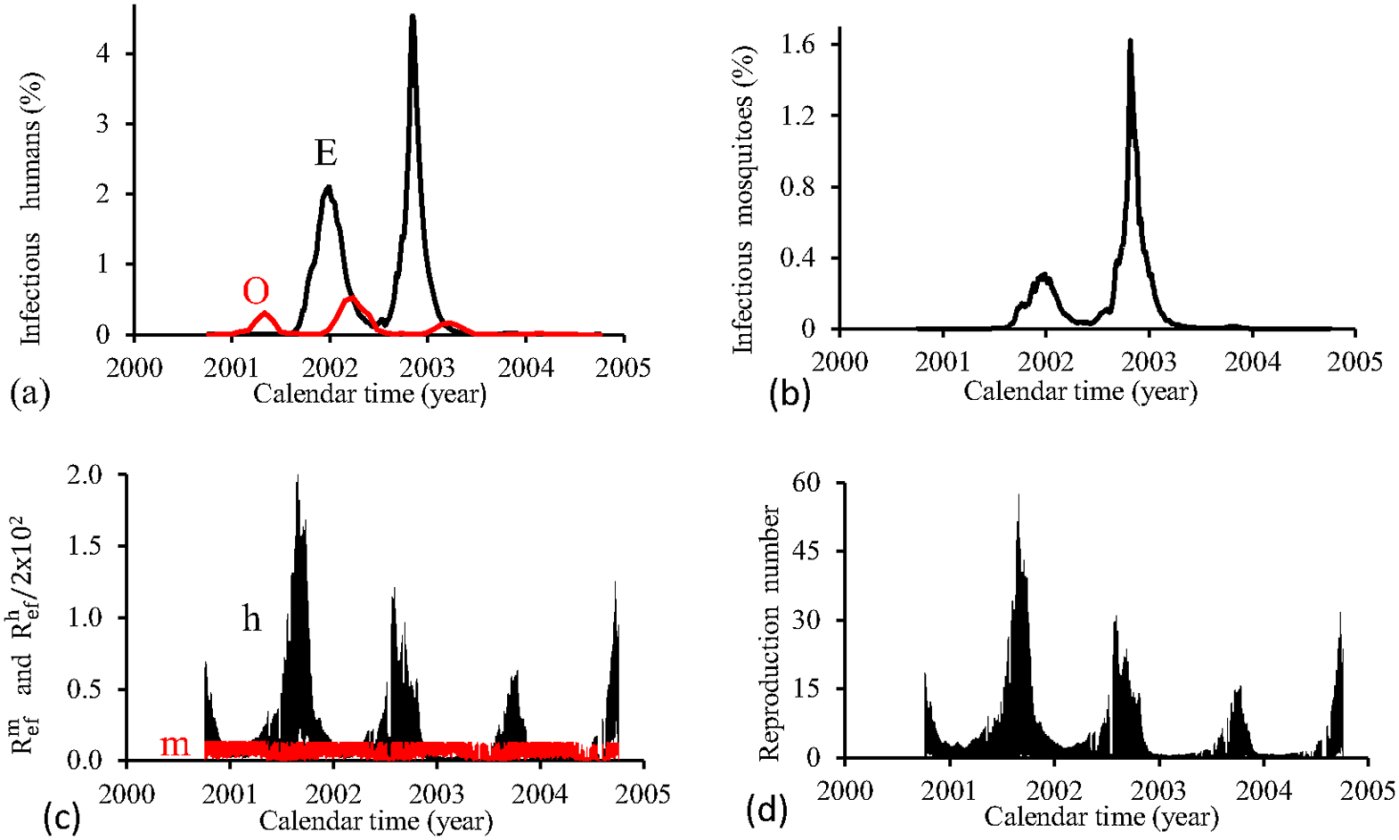
Estimation of Model SIR in Period 2 (October/2000—September/2004). (a) observed incidence (O) and adjusted *i* (E), (b) *m*_3_/*m*, (c) $R_{ef}^{h}$ (h) and $R_{ef}^{m}$ (m), and (d) $R_{ef}=R_{ef}^{h}\times R_{ef}^{m}$.

Fig 20 shows the fittings for Period 2 (2000–2004). The fitted second and third cycles (years 2002 and 2003) show much more severe epidemics than the real ones. The first and fourth peaks are absent. Again, the fitting is poor. The fraction of recovered individuals reaches 78.5% at the end of this period. The maximum peak of the proportion of infectious mosquitoes occurs between years 2002 and 2003, with value reaching 1.6%. The average partial effective reproduction numbers are ${\bar{R}}_{ef}^{m}=0.07$ and ${\bar{R}}_{ef}^{h}=29.3$, and for the overall number, ${\bar{R}}_{ef}=2.80$.

**Fig 21:**
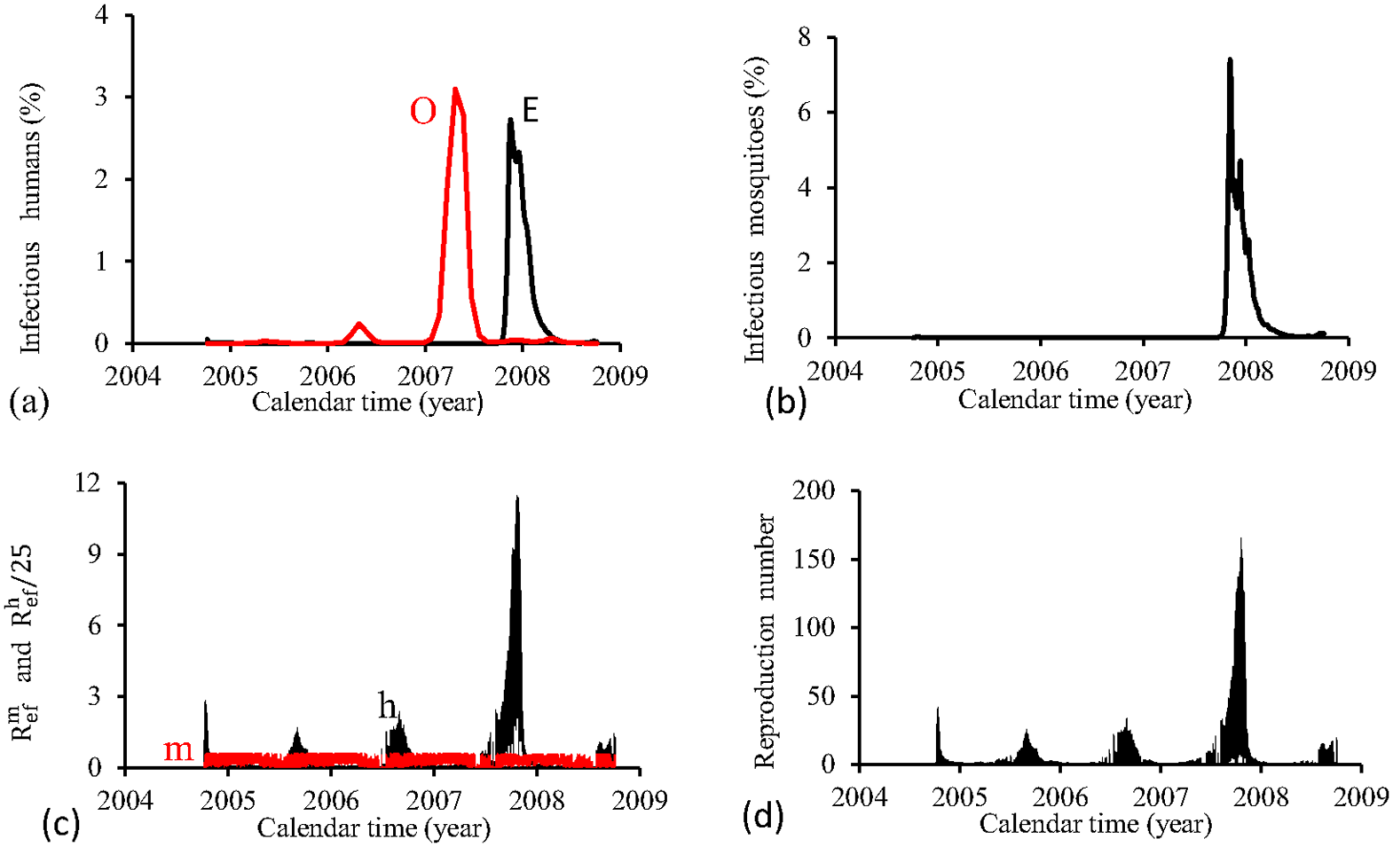
Estimation of Model SIR in Period 3 (October/2004– September/2008). (a) observed incidence (O) and adjusted *i* (E), (b) *m*_3_/*m*, (c) $R_{ef}^{h}$ (h) and $R_{ef}^{m}$ (m), and (d) $R_{ef}=R_{ef}^{h}\times R_{ef}^{m}$.

Fig 21 shows the fittings for Period 3 (2004–2008). The fitting is very poor, with a severe epidemics occurring half year later. The fraction of recovered individuals reaches 29.4% at the end of this period. The maximum peak of the proportion of infectious mosquitoes occurs between years 2007 and 2008, with value reaching 8%. The average partial effective reproduction numbers are ${\bar{R}}_{ef}^{m}=0.30$ and ${\bar{R}}_{ef}^{h}=9.61$, and for the overall number, ${\bar{R}}_{ef}=4.21$.

**Fig 22:**
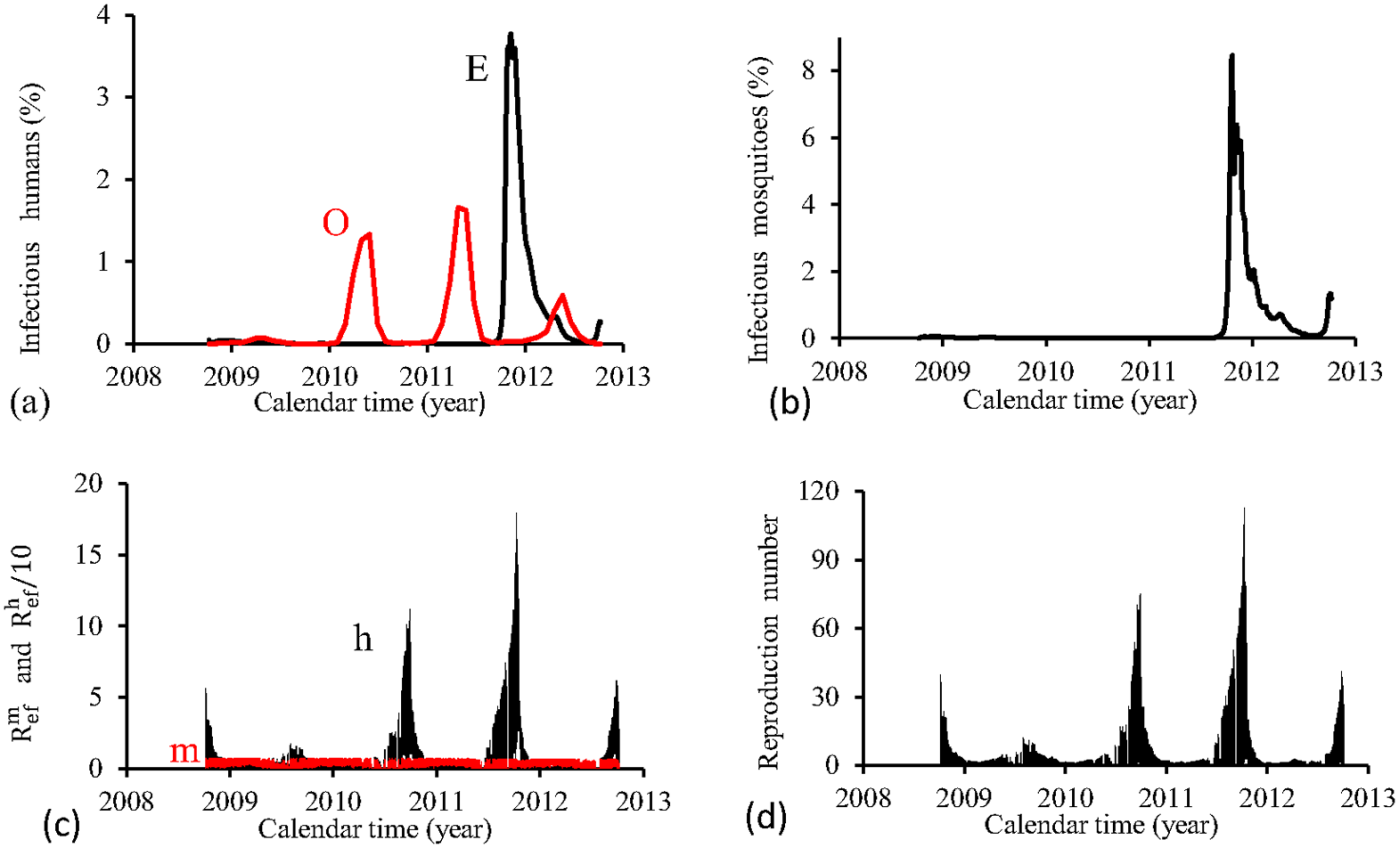
Estimation of Model SIR in Period 4 (October/2008—September/2012). (a) observed incidence (O) and adjusted *i* (E), (b) *m*_3_/*m*, (c) $R_{ef}^{h}$ (h) and $R_{ef}^{m}$ (m), and (d) $R_{ef}=R_{ef}^{h}\times R_{ef}^{m}$.

Fig 22 shows the fittings for Period 4 (2008–2012). Similar to the period 3, the fitting presents only one severe epidemics at the end of period. The fraction of recovered individuals reaches 44.7% at the end of this period. The maximum peak of the proportion of infectious mosquitoes occurs between years 2011 and 2012, with value reaching 8%. The average partial effective reproduction numbers are ${\bar{R}}_{ef}^{m}=0.40$ and ${\bar{R}}_{ef}^{h}=7.40$, and for the overall number, ${\bar{R}}_{ef}=3.74$.

The SIR model fitted very poorly in all periods. In the first and the second periods, the quotients of the magnitudes of the peaks of $R_{ef}^{h}$ and $R_{ef}^{m}$ are of order 10^4^ and 10^3^, respectively, while for other two periods, the order was 10^2^. The fraction of recovered individuals reached around 40% in first and fourth periods, with the highest 78% in the second period, and the lowest 30% in the third period. In this model, the beginning of annual epidemics occurs at the middle of the year.

Both TMAL and SIR models presented similar pattern of fittings for all periods. TMAL model presented plateau for both $R_{ef}^{h}$ and $R_{ef}^{m}$, but SIR model reverted the pattern found in FD and PMAL models: triangle shape for $R_{ef}^{h}$ and plateau for $R_{ef}^{m}$ curves. This change practically eliminated the overlapping of epidemics obtained from TMAL model, however, in all epidemics cycles, the increasing phase occurred in the winter season.

### Comparing models and corresponding fittings

In an autonomous modelling (constant size of populations and constant model parameters), the four models are equivalent. Let us label the transmission coefficients *β*_*m*_ and *β*_*h*_ with the superscript *F*, *T*, *P* and *S*, respectively for FD, TMAL, PMAL and SIR models. Then, in constant population sizes, with M* and N*, and transmission coefficients *β*_*m*_ and *β*_*h*_, if we have

$$\begin{array}{c} \left\{ \begin{array}{c} \beta_{m}=\beta_{m}^{F}=\beta_{m}^{T}=N^{*}\beta_{m}^{P}=\frac{\beta_{m}^{S}}{M^{*}}\\ \beta_{h}=\frac{M^{*}}{N^{*}}\beta_{h}^{F}=\beta_{h}^{T}=M^{*}\beta_{h}^{P}=\frac{\beta_{h}^{S}}{N^{*}}, \end{array}\right. \end{array}$$ (22)

then all models are equivalent.

Here, the non-autonomous models have parameters as well as the human and mosquito populations being time dependent. Two types of models were considered depending on the way the mosquito and human populations interaction is described. Type 1 modelling (FD and PMAL) considered the infection among susceptible humans being proportional to the density of infectious mosquitoes, that is, *m*_3_. In the Type 2 modelling (TMAL and SIR), the infection term was proportional to the fraction of infectious mosquitoes, that is, *m*_3_/*m*.

Let us discuss Type 1 and 2 modellings.

In Type 1 modelling, variables *D* and *N* appear differently in the dynamical systems: in FD model, the quotient *D*/*N* appears multiplying the density of the infectious mosquitoes *m*_3_, while in PMAL model, *m*_3_ is multiplied by only *D*, but the fraction of infectious individuals *i* comes multiplied by *N*. This difference in the equations, however, resulted in a quite similar fittings.

The total transmission coefficients estimated using FD model were 10^6^ order of magnitude in comparison with the coefficients fitted from PMAL model. In the latter model, the transmission coefficients are per-capita, but the order of *D* and *N* are 10^6^, and the total coefficients are per-capita coefficients multiplied by *D* and *N*.

FD model showed a better fittings than PMAL (*Er* was lower, except period 4, which is quite similar). Estimation with FD model resulted in lower risk of dengue, presenting lower final fraction of recovered individuals and mean reproduction number, in the latter case only period 4 resulted in higher ${\bar{R}}_{ef}$.

In FD and PMAL models, epidemics cycle approximately initiates at the beginning of the calendar year and ends in September, encompassing an epidemic period of around 9 months. There is clearly inter-epidemics periods of 3 months, with practically absent dengue cases.

In both models, when a severe epidemics (period 3) or epidemics with relatively same intensity (period 4) are present, then dengue cases are well adjusted. Periods 1 (other peaks are not negligible in comparison with period 3) and 2 (epidemics with decreasing intensity in comparison with period 4) did not fit so well. In general, both models presented good fittings.

Both models showed curves for $R_{ef}^{h}$ and $R_{ef}^{m}$ with similar pattern: curves with plateau for $R_{ef}^{h}$ and triangle shaped curves for $R_{ef}^{m}$. This is an expected finding, since the temperature and rain dependent parameters have little influence in human population (*s*), while mosquito population is strongly influenced (*m*_1_). Notice that the plateau in $R_{ef}^{h}$ is slightly decreased after epidemics, due to decrease in susceptible humans. The overall effective reproduction number *R*_*ef*_ follows the trend of $R_{ef}^{m}$. The value of *R*_*ef*_ is the potential risk of dengue epidemics, with the peak of the risk situating in March-April. The susceptible populations of humans and mosquitoes plus the entomological parameters determine the intensity of the maximum hazard.

In Type 2 modelling, variables *Dm*(= *M*) and *N* appear only in SIR model: *m*_3_/*m* is divided by *N*, but the fraction of infectious individuals *i* comes divided by *Dm*. In TMAL model, *m*_3_/*m* and *i* do not come accompanied by *M* and *N*. This difference in the equations resulted in different fittings.

In TMAL and SIR models, the incidence data were poorly fitted. When epidemics start, the annual cycles are overlapped in TMAL model, eliminating the inter-epidemics periods [22], but SIR model persistently initiated epidemics in unfavorable season. Type 2 modelling yielded fittings with more intense dengue epidemics than Type 1 modelling.

In TMAL model, the shape of the epidemics is generally similar than models of Type 1 (annual cycle begins at the beginning of the year and range of epidemics around 8 months), but there are non-zero transmission in winter seasons. The annual epidemics periods are highly overlapped, with the absence of inter-epidemic periods. However, in SIR model, epidemics cycle approximately begins in September/October of the calendar year and ends in April/May of the next year, encompassing an epidemic period of around 9 months, but practically eliminated the overlapping phenomenon.

The transmission of dengue epidemics in unfavorable seasons are due to the use of fraction of infectious mosquitoes. In unfavorable seasons, the number of infectious mosquitoes *M*_3_ (or its density *m*_3_ = *M*_3_/*D*) goes to zero. However, the fraction *m*_3_/*m* does not tend to zero, which results in non-zero risk of dengue transmission. This occurs in TMAL model. But, in SIR model, besides the fraction *m*_3_/*m*, there is another fraction *m*_1_/*m* (in the transmission term among humans). This term allowed humans to be strongly dependent on the abiotic variation with respect to dengue infection. This second fraction of mosquito population results in a more possibility of dengue transmission in unfavorable seasons. Hence, in SIR model, the dengue transmission during unfavorable seasons (inter-epidemics) is enhanced in comparison with TMAL model, but the overlapping is practically eliminated.

Notice that the triangle shape in Type 1 modelling for $R_{ef}^{m}$ was changed to a plateau in Type 2 modelling. The division *m*_1_/*m*, see Eq (13), somehow diminished the strong variation of mosquito population with temperature and rain, leading its quotient to a constant value. The plateau in Type 1 modelling for $R_{ef}^{h}$ was changed by a sharp triangle shape only in SIR model. The division *s*/*m*, see Eq (14), in somehow introduced a strong variation with temperature and rain in $R_{ef}^{h}$, and by the fact that $R_{ef}^{h}$ appears in the denominator, its effect is much enhanced, and the triangle shape is very sharp.

Both Type 1 and Type 2 modellings yielded similar mean values for the effective reproduction number, except periods 3 and 4 of SIR model. Type 1 modelling resulted in a triangle shape with relatively large basis for the risk of overall dengue (*R*_*ef*_). For this reason the peak of *R*_*ef*_ situated in the range 10–20. However, in Type 2 modelling, we obtained two shapes: a plateau in TMAL model, while a very sharp triangle shape is present in SIR model. For this reason, the peak of *R*_*ef*_ situated around 8 for TMAL model, and in the range 60–180 for SIR model. For this reason, SIR model presented the highest peaks of infection among humans and mosquitoes. In general, Type 2 modelling resulted in more severe dengue epidemics to explain the incidence data from the City of Campinas.

FD and PMAL (Type 1 modelling) reproduced abiotic fluctuation mainly in mosquito population ($R_{ef}^{m}$), while human population ($R_{ef}^{h}$) was less affected. Additionally, the risk of dengue (*R*_*ef*_) increases at the end of the year, and attains its maximum at month February. However, in Type 2 modelling, the risk of dengue is practically constant during entire year for TMAL model, but for SIR model, the highest risks of dengue situated in the middle or at the end of the year, with very narrow basis for *R*_*ef*_ curve.

Let us now discuss the fitting of the parameters *β*_*m*_, *β*_*h*_ and *prop*.

The quotient between the estimated values for *β*_*m*_ and *β*_*h*_ have magnitudes of order 6 according to the models considered. For PMAL model, where *β*_*m*_ and *β*_*h*_ are per-capita transmission coefficients, estimations of both parameters have order of 10^−9^/10^−8^. For FD and TMAL models, where *β*_*m*_ and *β*_*h*_ are total transmission coefficients, estimations of both parameters have order of 10^−3^/10^−2^. For SIR model, where *β*_*m*_ and *β*_*h*_ are bi-total transmission coefficients, estimations of both parameters have order of 10^3^/10^5^. The quotient of order of magnitude of 10^6^ results due to the fact that the human population is around 10^6^, as well as the value *D* for mosquito population. This difference is in accordance with Eq (22).

The estimated parameter *prop* and an arbitrary initial number of infectious individuals *I*_*d*_ play important role in the estimation process. The functional given by Eq (18) presents several minima, with values very close. Besides the best fittings for *β*_*m*_ and *β*_*h*_, we allowed to *prop* to vary in order to obtain the fraction of recovered individuals in the range 20–50% [23], and the peak of the fraction of infectious mosquitoes in the range 0.5–5% [24]. We believe that these ranges are biologically acceptable, than for instance, 95% or 2% of recovered individuals at the end of the epidemics, or a peak of 25% of infectious mosquitoes. In the schistosomes transmission modelling, biological acceptance was adopted to chose better fitting [25][26]. Finally, *I*_*d*_ was allowed to vary in order to adjust the initial phase of the epidemics (larger *I*_*d*_, earlier occurs the ascending phase of epidemics). This degree of freedom was introduced by the assumption that the entire population is under a new epidemics, hence the number of recovered individuals due to previous circulating serotype of dengue is set zero.

### Discussion

The incidence data recorded in the City of Campinas were split in four periods: Period 1 (1995–2000), Period 2 (2000–2004), Period 3 (2004–2008) and Period 4 (2008–2012), where each period begins at month October and ends at September. Each one of these four periods was fitted considering models with unique serotype of dengue. Type 1 modelling (FD and PMAL) fitted well all periods of incidence of dengue in the City of Campinas, differently from the fittings yielded by Type 2 modelling (TMAL and SIR).

Type 1 modelling showed a clear inter-epidemic period between consecutive annual epidemics cycles. The epidemics began at the end of the previous year, and ended at the middle of the next year. However, Type 2 modelling presented overlapping of epidemic cycles, and the initial phase of an epidemics could occur in unfavorable season.

By observing the partial effective reproduction numbers ($R_{ef}^{h}$ and $R_{ef}^{m}$), Type 1 modelling showed a strong seasonality in the risk of mosquitoes to be infected by dengue, but the risk of infection among humans was practically insensitive to seasonal variations. However, Type 2 modelling presented mosquitoes being insensitive to seasonal variations, but humans can be strongly dependent (SIR model) or not (TMAL model) on the seasonal variations with respect to dengue infection. Comparing the overall effective reproduction number (*R*_*ef*_), in Type 1 modelling there is a triangle shaped curve, however, a plateau (TMAL model) and very sharped triangle shape (SIR model) in Type 2 modelling.

We conclude that models FD and PMAL fitted better incidence data from the city of Campinas. The results yielded by these models are in better agreement with biological reasonings (periodicity of epidemics in favorable seasons, and well established inter-epidemics period during unfavorable seasons). This conclusion is valid for regions where there are strong variations in abiotic factors. When small abiotic variations occur, all models could be used indistinctly.

The prediction of next dengue epidemics is a hard task. We address this question, choosing the model FD estimated with respect to Period 1, considering the following factors: (1) temperature and precipitation, (2) initial number of infectious humans introduced in a community, and (3) the time of this introduction.

**Fig 23:**
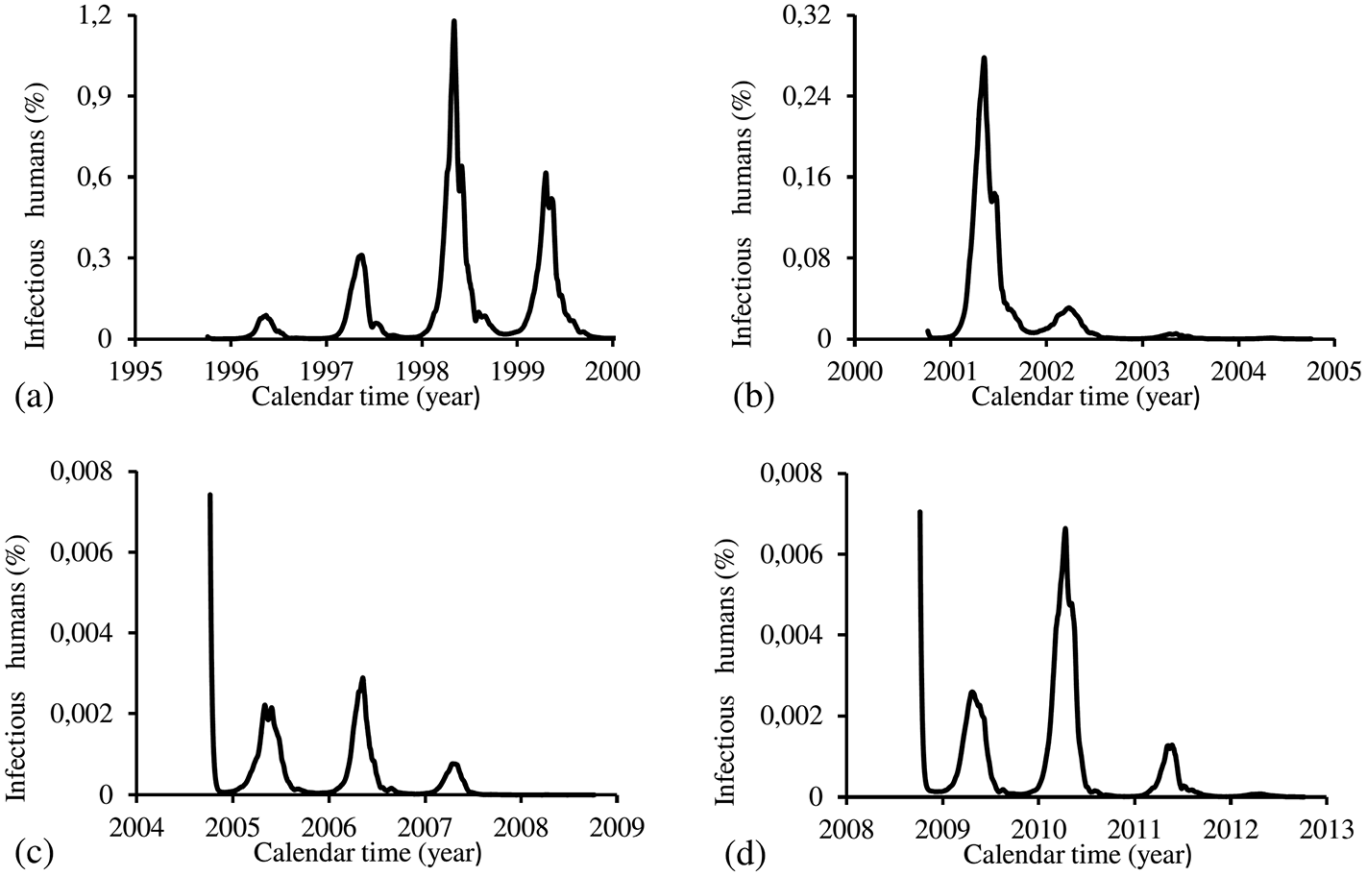
Effects of temperature and precipitation considering the fitted parameters of Model FD in Period 1 (October/1995—September/2000). (a) Fig 7(a) restricted in the interval October/1995—September/1999 (four years), (b) Period 2 (October/2000—September/2004), (c) Period 3 (October/2004—September/2008), and (d) Period 4 (October/2008—September/2012).

To evaluate the influence of temperature and precipitation on the incidence of dengue, we fix the estimated parameters *β*_*m*_ and *β*_*h*_ for Period 1 of Model FD and the initial number *I*_*d*_ of infectious humans (see first column of Table 6). Using these fixed values, we obtain the fraction of infectious individuals *i* for Periods 2, 3 and 4. The time of introduction of infectious individuals in these periods is October 1^*st*^ of the initial year of the corresponding period. Fig 23 shows the strong influence of temperature and precipitation. Notably, period 2 presents lower risk of dengue incidence than period 1 (approximately one fourth), while periods 3 and 4 are practically safe from dengue incidence. In these last periods, temperature and precipitation affected quite similarly, being the last period slightly more hazardous. Fig 23(a) shows Fig 7(a) restricted to years 1995 to 2000.

**Fig 24:**
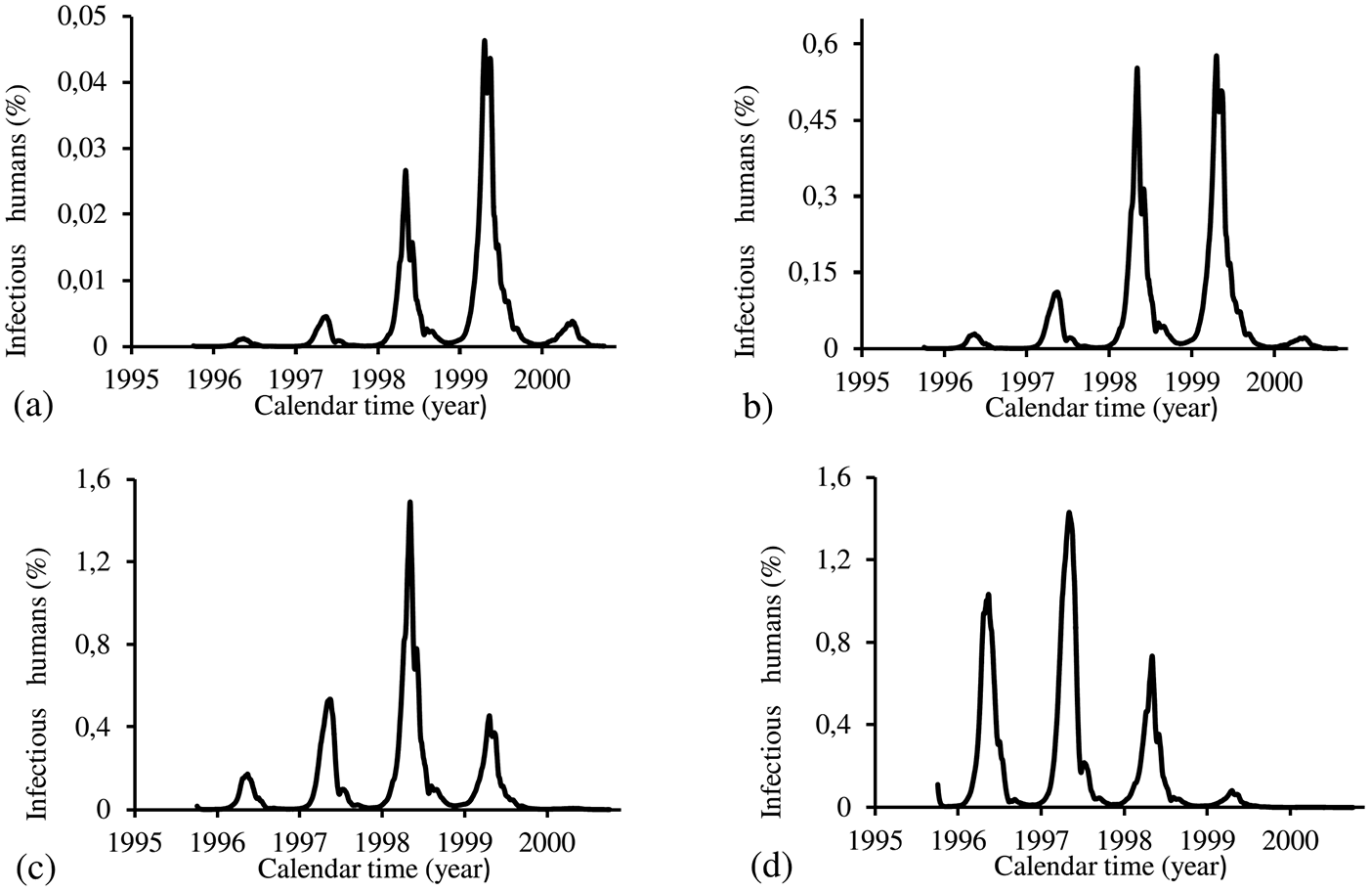
Effects of initial introduction of infectious individuals *I*_*d*_ considering the fitted parameters of Model FD in Period 1. (a) *I*_*d*_ = 1, (b) *I*_*d*_ = 25, (c) *I*_*d*_ = 150, and (d) *I*_*d*_ = 1000. (Fig 7(a) was obtained using *I*_*d*_ = 75).

Another source of uncertainty is the number of infectious individuals that trigger an epidemics. To evaluate the influence of this number, we consider only Model FD with estimations given in Period 1, by varying *I*_*d*_. Fig 24 shows the fraction of infectious individuals *i* for: *I*_*d*_ = 1 (a), *I*_*d*_ = 25 (b), *I*_*d*_ = 150 (c), and *I*_*d*_ = 1000 (d). As *I*_*d*_ increases, the risk of dengue incidence increases, anticipating the highest peak of epidemics. For small *I*_*d*_ (Fig 24(a) and 24(b)), the most severe epidemics occurs after 4 years of the introduction of dengue cases, and as *I*_*d*_ increases, the highest peak is anticipated: In Fig 24(c) and also Fig 7(a), the highest peak is anticipated in one year, while in Fig 24(d), in two years.

**Fig 25:**
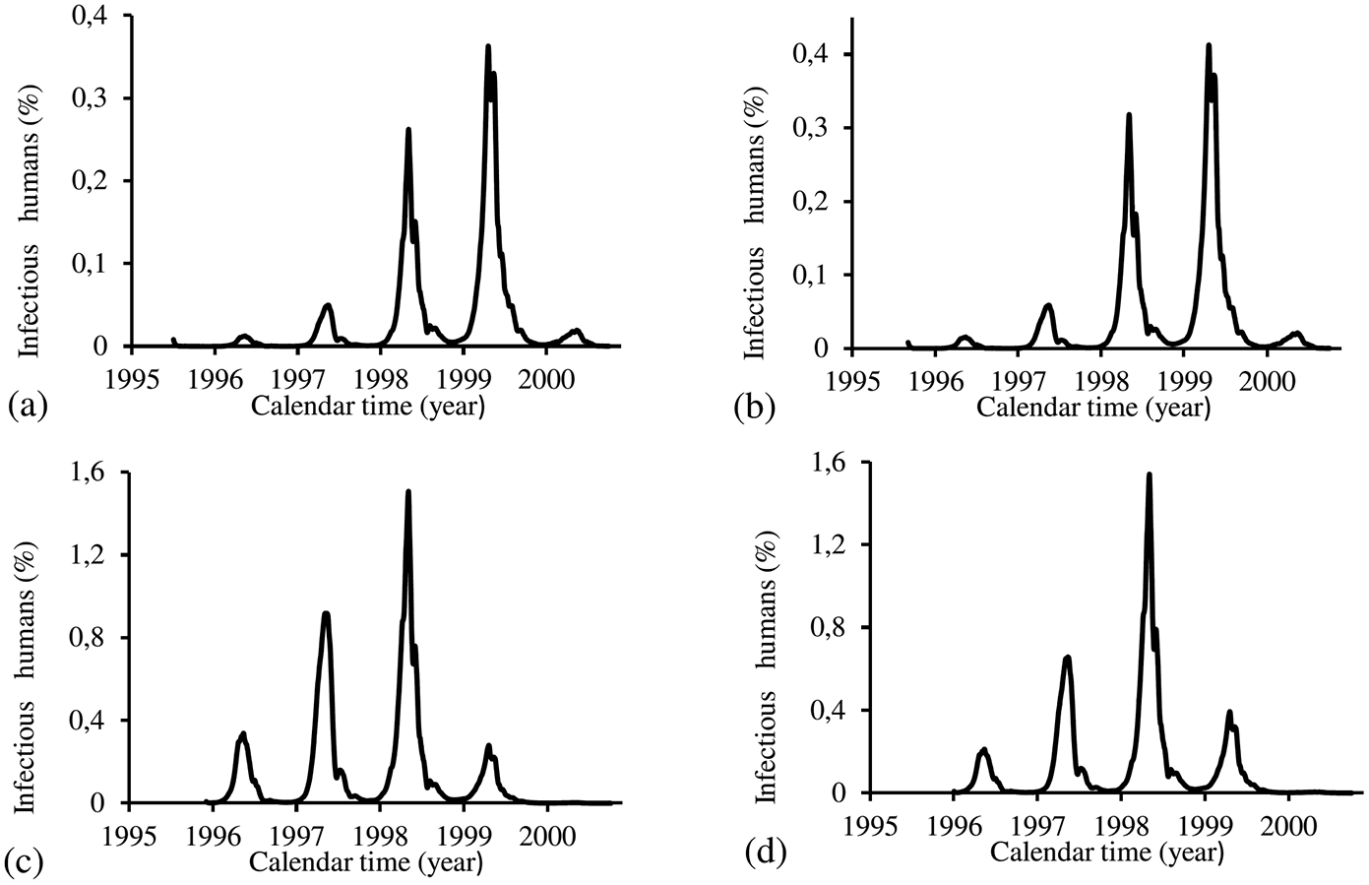
Effects of the time of introduction of infectious individuals *t*_1_ considering the fitted parameters of Model FD in Period 1. The time of introduction *t*_1_ is year 1995: (a) July 1^*st*^, (b) September 1^*st*^, (c) December 1^*st*^, and (d) December 31^*st*^. (Fig 7(a) was obtained using October 1^*st*^ 1995 as *t*_1_).

Finally, let us evaluate how the time of the introduction of infectious humans affects the incidence. Again, we consider only Model FD with estimations given in Period 1, now varying *t*_1_. Fig 25 shows the fraction of infectious individuals *i* for *t*_1_ corresponding to year 1995, in the day: July 1^*st*^ (a), September 1^*st*^ (b), December 1^*st*^ (c), and December 31^*st*^ (d). We observe a pattern similar than the one in the previous case. Notably, if dengue cases are introduced in the beginning of December (Fig 25(c)), the first epidemics is as severe as the highest peak occurred in Fig 25(a) and 25(b), cases of early introduction of dengue. By delaying the introduction of dengue cases, we observe that initial peaks are decreasing, but latter epidemics are more severe (Compare Fig 25(c) and 25(d)).

In future works, the previous models will be improved by introducing the concomitant action of two or more serotypes of dengue instead of just one serotype as in the present work, in order to fit the entire period of dengue incidence (1995–2012) recorded from the City of Campinas. A feature of the estimation procedure used in the present work was the introduction of a parameter *prop* in order to encompass the unnotified cases of dengue. However, another possibility, also to be considered in a future work, is to modify the model by introducing a compartment of mild cases of dengue, which could be considered asymptomatic [27].

In this paper, the temperature dependency of the entomological parameters were well defined from experiments conducted in laboratory. However, the parameters depending on precipitation were chosen based on simulations [14]. Sensitivity analysis of these parameters must be done to evaluate their effects on the dengue transmission (especially in extreme conditions).

## Supporting Information

S1 Text Appendix containing mathematical analysis of autonomous model and description of the estimation method. (PDF)
