# Supplementary material for: Fitting the Incidence Data from the City of Campinas, Brazil, Based on Dengue Transmission Modellings Considering Time-Dependent Entomological Parameters
Source: PLoS One. 2016 Mar 24;11(3):e0152186. doi: 10.1371/journal.pone.0152186 (PMC4807111; doi:10.1371/journal.pone.0152186)
Supplement: S1 Text — (PDF) [file pone.0152186.s001.pdf]

Fitting the incidence data from the City of Campinas, Brazil, based on dengue transmission modellings considering time-dependent entomological parameters – Supporting Information

Hyun Mo Yang,[1*] José Luiz Boldrini,[1] Artur Cesar Fassoni,[1,4]
Luiz Fernando Souza Freitas,[1] Miller Ceron Gomez,[1]
Karla Katerine Barboza de Lima,[1] Valmir Roberto Andrade[2]
and André Ricardo Ribas Freitas[3]

[1]Universidade Estadual de Campinas (UNICAMP)
[2]Superintendência de Controle de Endemias (SUCEN)
[3]Coordenadoria de Vigilância Sanitária (COVISA)
[4]Universidade Federal de Itajubá (UNIFEI)

Autonomous model – constant sizes of populations and constant parameters

For reasons describe in the main text, we assume here that all the parameters of the system of equations (5), (6), (7), (8) and (9) from the main text are constants, and let us consider the corresponding existence of steady states.

Let us analyze human and mosquito populations in interaction in the special situation when dengue virus is not circulating. In this case, the equations are

$$\left\{\begin{array}{rcl} \frac{d}{dt}l & = & qf\phi_m m\left(1-\frac{l}{C}\right)-\left(\sigma_l+\mu_l^c\right)l \\ \frac{d}{dt}p & = & \sigma_l^c l-\left(\sigma_p+\mu_p^c\right)p \\ \frac{d}{dt}m & = & \sigma_p^c p-\mu_f^s m, \end{array}\right.$$

for mosquito population, and, from equation (4), $dN/dt=\left(\phi_h-\mu_h\right)N$ for humans, which obeys $N=N_0e^{(\phi_h-\mu_h)t}$, where N_0 is the size of population at $t=0$. Human population varies,

*Corresponding author: hyunyang@ime.unicamp.br; tel/fax: + 55 19 3521-6031. Address: UNICAMP – IMECC – DMA, Praça Sérgio Buarque de Holanda, 651, CEP: 13083-859, Campinas, SP, Brazil

but mosquito population attains a steady state given by

$$\begin{cases} l^* &= C\left(1-\frac{1}{Q_c}\right) \\ p^* &= \frac{\sigma_l}{\sigma_p+\mu_p^c}C\left(1-\frac{1}{Q_c}\right) \\ m^* &= \frac{\sigma_p}{\mu_f^s}\frac{\sigma_l}{\sigma_p+\mu_p^c}C\left(1-\frac{1}{Q_c}\right), \end{cases} \tag{S1}$$

where the offspring number Q_c is given by

$$Q_c = \frac{\sigma_l}{\sigma_l+\mu_l^c}\frac{\sigma_p}{\sigma_p+\mu_p^c}\frac{qf\phi_m}{\mu_f^s}.$$

It was shown that this equilibrium is locally asymptotically stable for $Q_c > 1$; otherwise, when $Q_c < 1$, the extinction of mosquitoes, the equilibrium $(l^* = 0, p^* = 0, m^* = 0)$, is stable. Removing the control mechanisms ($\mu_l^c = \mu_l$ and $\mu_p^c = \mu_p$), the basic offspring number Q_0 is yielded as

$$Q_0 = \frac{\sigma_l}{\sigma_l+\mu_l}\frac{\sigma_p}{\sigma_p+\mu_p}\frac{qf\phi_m}{\mu_f}. \tag{S2}$$

Now consider that the size of human population is also contant, which is obtained by letting $\phi_h = \mu_h$. In this case, the constant size of human population is $N_0 = N^*$. Hence, the steady states for all sub-models can be obtained.

The trivial equilibrium P^0, or disease free equilibrium (DFE), is given by

$$P^0 = \left(l^*, p^*, m_1^* = m^*, m_2^* = 0, m_3^* = 0, s^* = 1, e^* = 0, i^* = 0\right),$$

where l^*, p^* and m^* are given by equation (S1).

The non-trivial equilibrium P^*, or endemic equilibrium, is unique given by

$$P^* = \left(l^*, p^*, m_1^*, m_2^*, m_3^*, s^*, e^*, i^*\right),$$

where the coordinates are

$$\begin{cases} m_1^* &= \frac{\sigma_p}{b_m\phi_m i^*+\mu_f^s}\frac{\sigma_l}{\sigma_p+\mu_p^c}C\left(1-\frac{1}{Q_c}\right) \\ m_2^* &= \frac{b_m\phi_m i^*}{\gamma_m+\mu_f^d}\frac{\sigma_p}{b_m\phi_m i^*+\mu_f^s}\frac{\sigma_l}{\sigma_p+\mu_p^c}C\left(1-\frac{1}{Q_c}\right) \\ m_3^* &= \frac{\gamma_m}{\mu_f^d}\frac{b_m\phi_m i^*}{\gamma_m+\mu_f^d}\frac{\sigma_p}{b_m\phi_m i^*+\mu_f^s}\frac{\sigma_l}{\sigma_p+\mu_p^c}C\left(1-\frac{1}{Q_c}\right) \\ s^* &= 1-\frac{(\sigma_h+\mu_h)(\gamma_h+\mu_h)}{\gamma_h\mu_h}i^* \\ e^* &= \frac{\sigma_h+\mu_h}{\gamma_h}i^* \\ i^* &= \frac{\gamma_h\mu_h\mu_f^d(R_c-1)}{\mu_f^d(\sigma_h+\mu_h)(\gamma_h+\mu_h)R_c+\gamma_h\mu_h b_m\phi_m}, \end{cases} \tag{S3}$$

with the reproduction number R_c being given by

$$R_c = \frac{b_h\phi_m}{\mu_f^d}\frac{\gamma_h}{\gamma_h+\mu_h}\frac{b_m\phi_m}{\sigma_h+\mu_h}\frac{\gamma_m}{\gamma_m+\mu_f^d},$$

and, removing the control mechanisms ($\mu_l^c = \mu_l$, $\mu_p^c = \mu_p$ and $\mu_f^s = \mu_f^d = \mu_f$), the basic reproduction number R_0 becomes

$$R_0 = \frac{b_h \phi_m}{\mu_f} \frac{\gamma_h}{\gamma_h + \mu_h} \frac{b_m \phi_m}{\sigma_h + \mu_h} \frac{\gamma_m}{\gamma_m + \mu_f}. \tag{S4}$$

The parameters b_m and b_h stand for generic transmission parameters, which must be substituted according to four assumptions related to incidence of dengue. The values l^*, p^* and $m^* = m_1^* + m_2^* + m_3^*$ are given by equation (S1).

The stability analysis, by applying the Routh-Hurwitz criteria, can be found in [1] [2] [3]. Briefly, the trivial equilibrium P^0 is locally asymptotically stable for $R_c < 1$, otherwise, for $R_c > 1$, the endemic equilibrium P^0 is locally asymptotically stable. At $R_c = 1$, there is a forward bifurcation, from P^0 to P^*. The coordinates of the equilibrium point P^* is valid when $\mu_f^s = \mu_f^d$.

Biological interpretation of the basic reproduction number R_0 follows. Notice that the term $b_h \phi_m / \mu_f$ is the average number of humans infected by one infectious mosquito during her entire lifespan; and the term $\gamma_h / (\gamma_h + \mu_h)$ is the probability of these infected persons surviving the exposed class and entering to the infectious class. Hence, the product of these two terms is the average number of infectious humans produced by one infectious mosquito introduced in a community free of dengue. The term $b_m \phi_m / (\sigma_h + \mu_h)$ is the average number of mosquitoes infected by one infectious human during his/her infectious period; and the term $\gamma_m / (\gamma_m + \mu_f)$ is the probability of these infected mosquitoes surviving the exposed class and entering to the infectious class. Thus, the product of these last two terms is the average number of infectious mosquitoes produced by one infectious human introduced in a community free of dengue. Therefore, R_0 gives the average number of secondary infectious humans (or mosquitoes) produced by one primary infectious human (or mosquito) introduced in completely susceptible populations of humans and mosquitoes.

The combination of s^* and m_1^*, given by equation (S3), and m^*, equation (S1), results in

$$s^* \frac{m_1^*}{m^*} \equiv \chi_c = \frac{1}{R_c}, \tag{S5}$$

that is, in the endemic steady state, the product of the fractions of susceptible humans and mosquitoes (χ_c) is equal to the inverse of the reproduction number (if controls are removed, then $\chi_0 = 1/R_0$). In directly transmitted infections, this type of relation is well established [4] [5].

Let the above solutions be separated according to the transmission hypotheses considered in the main text, that is, to each one of the models.

For model FD, we have $b_m = \beta_m M^*/N^*$ and $b_h = \beta_h$. The basic reproduction number is

$$R_0 = \frac{\beta_h \phi_m}{\mu_f} \frac{\gamma_h}{\gamma_h + \mu_h} \frac{\beta_m \phi_m \frac{M^*}{N^*}}{\sigma_h + \mu_h} \frac{\gamma_m}{\gamma_m + \mu_f},$$

and the partial reproduction numbers can be defined by

$$(\text{FD}) : \begin{cases} R_0^h &= \frac{\beta_h \phi_m}{\mu_f} \frac{\gamma_h}{\gamma_h + \mu_h} \\ R_0^m &= \frac{\beta_m \phi_m}{\sigma_h + \mu_h} \frac{\gamma_m}{\gamma_m + \mu_f} \frac{M^*}{N^*}. \end{cases} \tag{S6}$$

Dimensions of partial numbers are: $\left[R_0^h\right] = [M]^{-1}[N]$ and $\left[R_0^h\right] = [M][N]^{-1}$.

For model PMAL, we have $b_m = \beta_m M^*$ and $b_h = \beta_h N^*$. The basic reproduction number is

$$R_0 = \frac{\beta_h \phi_m N^*}{\mu_f} \frac{\gamma_h}{\gamma_h + \mu_h} \frac{\beta_m \phi_m M^*}{\sigma_h + \mu_h} \frac{\gamma_m}{\gamma_m + \mu_f},$$

and the partial reproduction numbers are

$$(\text{PMAL}) : \begin{cases} R_0^h &= \frac{\beta_h \phi_m}{\mu_f} \frac{\gamma_h}{\gamma_h + \mu_h} N^* \\ R_0^m &= \frac{\beta_m \phi_m}{\sigma_h + \mu_h} \frac{\gamma_m}{\gamma_m + \mu_f} M^*. \end{cases} \tag{S7}$$

Dimensions of partial numbers are the same as FD model.

For model TMAL, we have $b_m = \beta_m$ and $b_h = \beta_h$. The basic reproduction number is

$$R_0 = \frac{\beta_h \phi_m}{\mu_f} \frac{\gamma_h}{\gamma_h + \mu_h} \frac{\beta_m \phi_m}{\sigma_h + \mu_h} \frac{\gamma_m}{\gamma_m + \mu_f},$$

and the partial reproduction numbers are

$$(\text{TMAL}) : \begin{cases} R_0^h &= \frac{\beta_h \phi_m}{\mu_f} \frac{\gamma_h}{\gamma_h + \mu_h} \\ R_0^m &= \frac{\beta_m \phi_m}{\sigma_h + \mu_h} \frac{\gamma_m}{\gamma_m + \mu_f}. \end{cases} \tag{S8}$$

Partial numbers R_0^h and R_0^h are dimensionless with respect to dimension of populations.

For model SIR, we have $b_m = \beta_m / N^*$ and $b_h = \beta_h / M^*$. The basic reproduction number is

$$R_0 = \frac{\beta_h \phi_m \frac{1}{M^*}}{\mu_f} \frac{\gamma_h}{\gamma_h + \mu_h} \frac{\beta_m \phi_m \frac{1}{N^*}}{\sigma_h + \mu_h} \frac{\gamma_m}{\gamma_m + \mu_f},$$

and the partial reproduction numbers are

$$(\text{SIR}) : \begin{cases} R_0^h &= \frac{\beta_h \phi_m}{\mu_f} \frac{\gamma_h}{\gamma_h + \mu_h} \frac{1}{M^*} \\ R_0^m &= \frac{\beta_m \phi_m}{\sigma_h + \mu_h} \frac{\gamma_m}{\gamma_m + \mu_f} \frac{1}{N^*}. \end{cases} \tag{S9}$$

Dimensions of partial numbers are the same as FD model.

From the partial reproduction numbers in humans (R_0^h) and mosquitoes (R_0^m) defined for above sub-models, the basic reproduction number R_0 can be defined as

$$R_0 = R_0^h \times R_0^m, \tag{S10}$$

which is dimensionless with respect to dimension of populations.

Estimation method

The parameters β_m, β_h and *prop* are obtained by using an algorithm programmed in Matlab [6] and consisted of a suitable iteration scheme using the command $fminsearch(fun, X_0, options)$. This command uses the Nelder-Mead simplex algorithm to find a minimum point X_{min} of a

given functional $fun(X)$, using a given X_0 as the initial point for the Nelder-Mead iterations, and *options = optimset('TolX',tolx,'TolY',toly)* is the set of suitably chosen stopping tolerances for $fminsearch(fun, X_0, options)$. Here, *tolx* is the attributed value for the Matlab internal parameter *'TolX'*, which sets the tolerance for the difference of two subsequent approximations of the minimum point, and, also, *toly* is the attributed value for the Matlab internal parameter *'TolY'*, which sets the tolerance for changes in the value of functional $fun(X)$ during iterations.

Our scheme uses X as the set of parameters $(\beta_m, \beta_h, prop)$ and $X_0 = (\beta_m^0, \beta_h^0, prop^0)$ as the initial point with $\beta_m^0 \in [a, b]$, $\beta_h^0 \in [c, d]$ and $prop^0 \in [A, B]$. The functional $fun(X)$ to be minimized is given by equation (18) in the main text. The numerical simulations of dynamical systems are done by using the Matlab command *ode15s*.

However, the straightforward idea of just calling once the Matlab command *fminsearch* with the previously defined functional $Er(X)$, initial point X_0 and options *options* to obtain an approximation of the minimal point does not work well. In fact, in this procedure the iterations almost do not change the initial point X_0 because the functional $Er(X)$ is very badly behaved, presenting many and many small size oscillations that trap the generated approximations very near any taken initial point, and, also, almost flat regions steep and deep valleys. Fig S1 illustrates graph of $Er(X)$ as a function of β_m and β_h for four models.

Thus, it is necessary to use another strategy for searching the minimum point. To deal with the previously described difficulties, we appeal to a rather simple surrogate strategy related to convex relaxations. This is based on the idea of approximating the required minimum point by a sequence of minimum points for locally better behaved functionals $S_n(X)$, $n \geq 1$, chosen to approach $Er(X)$ as $n \to +\infty$, and obtained by adding to $Er(X)$ a suitable negative convex functions $s_n(X)$.

More precisely, since we already know that the parameters $X = (\beta_m, \beta_h, prop)$ must be in the set $\Lambda = [a, b] \times [c, d] \times [A, B]$, for each $n = 1, 2, \cdots$, we take

$$S_n(X) = Er(X) + s_n(X), \quad X \in \Lambda, \tag{S11}$$

where as before $Er(X)$ is the functional (15) in the main text, and $s_n(X)$ is the following convex function defined as

$$s_n(X) = c_n^{(1)}(\beta_m - a)(\beta_m - b) + c_n^{(2)}(\beta_h - c)(\beta_h - d) + d_n(prop - A)(prop - B), \tag{S12}$$

with $c_n^{(1)} \geq 0$, $c_n^{(2)} \geq 0$ and $d_n \geq 0$, which is nonpositive in Λ.

The idea behind this approach is that, depending on the values of the coefficients $c_n^{(1)}$, $c_n^{(2)}$ and d_n, the addition of this convex function "kills" small oscillations of the original functional $Er(X)$, and, at the same time, makes a local convex bending of any possible flatten directions in the subregion in the parameter space we are looking for the minimum point. Moreover, as c_n and d_n decrease to zero, $S_n(X) \leq Er(X)$, and $S_n(X)$ approaches monotonically to $Er(X)$. This improves the performance of the searching for the minimum.

Therefore, our strategy will be the following: starting with a given $X_0 \in \Lambda$ in a region of the parameter space where the minimum point is located, we start by minimizing the modified functional $S_1(X)$ by using a call of the Matlab command *fminsearch* (and, consequently, associated calls of the Matlab command *ode15s*); this gives a minimum point X_1 for $S_1(X)$ in Λ.

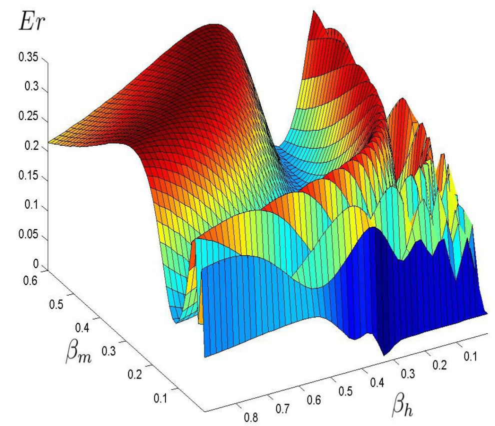

(a) FD

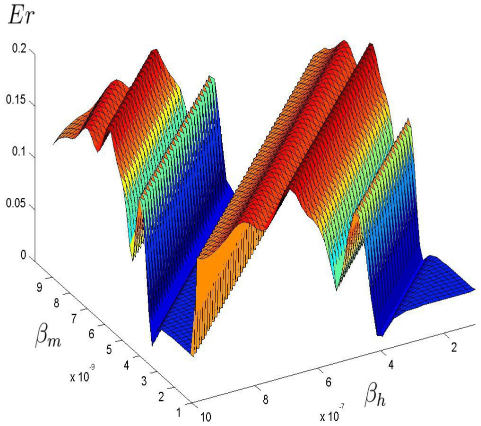

(b) PMAL

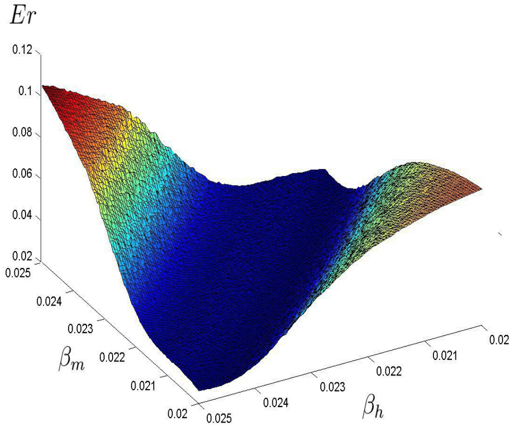

(c) TMAL

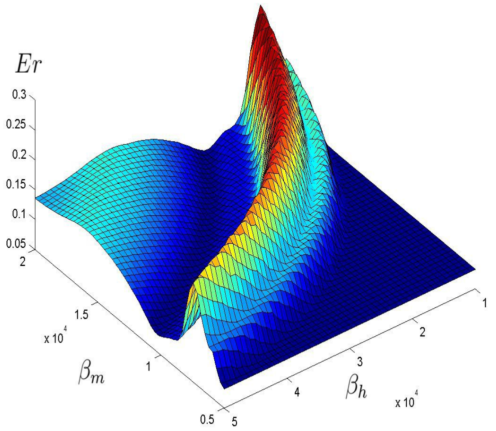

(d) SIR

Figure S1: Illustrative graphs of $Er(X)$: (a) FD ($prop = 100.0$), (b) PMAL ($prop = 102.2$, (c) TMAL ($prop = 294.4$), and (d) SIR ($prop = 268.3$) models.

Next, by taking $c_2^{(1)}$, $c_2^{(2)}$ and d_2 respectively slightly smaller than $c_1^{(1)}$, $c_1^{(2)}$ and d_1, and starting with a X_1, we minimize $S_2(X)$ with a further call of the Matlab command *fminsearch*; this gives a minimum point X_2 for $S_2(X)$ in Λ. From successively decreasing $c_n^{(1)}$, $c_n^{(2)}$ and d_n, calling *fminsearch* at each iteration, we obtain a sequence $\{X_n\}$ converging to the required minimum. In practice we have to bound the maximum number of such cycles, and the stopping criteria must include the relative smallness of $s_n(X)$ with respect to $Er(X)$ at each approximation of a minimum point.

These ideas are described in detail in the following, and, as usual, "=" means attribution.

1. Define the Matlab functions associated to the dynamical model being considered – They

depend on the parameters $X = (\beta_m, \beta_h, prop)$.

2. Define a Matlab function $Er(X)$ associated to the functional in (15) – This requires a call of the Matlab command *ode15s*.

3. Define a cycle counter variable: $ncycle = 1$, and set the maximum number of cycles to be $nmax \geq 1$.

4. Set the tolerances for the stopping criteria for the cycle iterations: $\epsilon > 0$, $\epsilon^{(1)} > 0$, $\epsilon^{(2)} > 0$, $\epsilon_D > 0$ and $\epsilon_s > 0$.

   The first one, $\epsilon > 0$, is used for the relative differences between two consecutive approximations X_n and X_{n-1} as described. The next three, $\epsilon^{(1)} > 0$, $\epsilon^{(2)} > 0$ and $\epsilon_D > 0$, are used to respectively check the smallness of the coefficients $c_n^{(1)}$, $c_n^{(2)}$ and d_n. Finally, ϵ_s is used to check the relative smallness of $s_n(X)$ with respect to $Er(X)$ at the approximation obtained in the n-the cycle iteration, that is, whether $|s_n(X_n)| \leq \epsilon_s Er(X_n)$.

5. Take the initial point $X_0 = (\beta_m^0, \beta_h^0, prop^0)$ – Define auxiliary variables X_{before} and X_{after}, and set initially $X_{before} = X_0$.

6. Define the initial options for *fminseach* – Set the initial values for $tolx = tolx_0$ and $toly = toly_0$, and define *options= optimset('TolX',tolx,'TolFun',toly)*.

   The initial values for the tolerances $tolx_0$ and $toly_0$ are taken not too small since it is not necessary to spend much cpu time to be rather precise in the initial steps of the algorithm when the coefficients of the convex perturbation are relatively large. To increase the required precisions after each cycle iteration, define and set a reduction factor $0 < red < 1$ for $tolx$ and $toly$, and set also lower bounds for such reductions: $tolx_{min}$ and $toly_{min}$.

7. Set $c_1^{(1)}$, $c_1^{(2)}$ and d_1, and define $C^{(1)} = c_1^{(1)}$, $C^{(2)} = c_1^{(2)}$ and $D = d_1$.

   Set reduction factors $0 < \eta^{(1)}, \eta^{(2)} < 1$ respectively for the coefficients $C^{(1)}$, $C^{(2)}$ and $0 < \eta_D < 1$ for D after each cycle iteration. Such reduction factors may vary during the cycle iterations.

8. Define the functional

$$\begin{aligned} S(X) = \;& Er(X) + C^{(1)}(\beta_m - a)(\beta_m - b) + C^{(2)}(\beta_h - c)(\beta_h - d) \\ & + D(prop - A)(prop - B). \end{aligned}$$

9. Call the Matlab command $X_{after} = fminseach(S(X), X_{before}, options)$. This gives the next approximate minimum point $X_{after} \in \Lambda$.

10. Check the stopping criteria for the cycle iterations:

    If $||X_{after} - X_{before})||/||X_{after}|| < \epsilon$, and $C(1) < \epsilon^{(1)}$, $C(2) < \epsilon^{(2)}$, $D < \epsilon_D$ and $|s_n(X_{after})| \leq \epsilon_s Er(X_{after})$, take X_{after} as the (approximate) global minimum X_{min} and stop the program.

If one of the previous conditions is not satisfied, check wether $ncycle = nmax$. If it is so, send a message declaring that the number of cycles reached its maximum without attaining an adequate approximate solution and stop the program.

Otherwise, redefine

$ncycle = ncycle + 1$, $X_{before} = X_{after}$, $X_0 = X_{after}$, $C^{(1)} = \eta^{(1)}C^{(1)}$, $C^{(2)} = \eta^{(2)}C^{(2)}$ and $D = \eta_D D$, $tolx = \max\{red * tolx, tolx_{min}\}$, and $toly = \max\{red * toly, toly_{min}\}$, *options= optimset('TolX',tolx, 'TolFun',toly)*, and then return to Item 8 and repeat the steps in the sequence.

This last algorithm scheme was the basis of a Matlab program to find the parameters $X = (\beta_m, \beta_h, prop)$ for each of our dynamical models under the following circumstances.

Actual data used in the program:

- For all dynamical models the computations were done with

  Maximum number of cycle iterations: $nmax = 200$.

  Tolerances for the stopping criteria for the cycle interactions: $\epsilon = 10^{-4}$, $\epsilon^{(1)} = \epsilon^{(2)} = 10^{-2}$, $\epsilon_D = 10^{-4}$ and $\epsilon_s = 5 \times 10^{-2}$.

  Reduction factors for the coefficients of the convex perturbation of the functional: $\eta^{(1)} = \eta^{(2)} = 0.8$ and $\eta_D = 0.75$.

  Initial values for the Matlab internal parameters for fminseach were taken as $tolx = 10^{-1}$ and $toly = 5 \times 10^{-1}$.

  Reduction factor for the tolerances $redtolx = 0.9$ and $redtoly = 0.82$.

  Minimum values for the tolerances tolx and toly: $tolxmin = 10^{-3}$ and $tolymin = 10^{-4}$

  Parameters $c_1 = 10$ and $d_1 = 0.1$.

- The intervals described in (17) for each of the dynamical models and periods are given in Tables S1, S2, S3 and S4.

Table S1: **Parameters a, b, c, d, A and B for Model** FD **for four periods.**

Param.	Period 1	Period 2	Period 3	Period 4
a	1×10^{-3}	3.9×10^{-4}	1×10^{-4}	2.5×10^{-3}
b	4×10^{-2}	9×10^{-3}	6×10^{-1}	9.5×10^{-3}
c	8×10^{-3}	3×10^{-2}	2×10^{-3}	3.5×10^{-2}
d	1×10^{-1}	8.3×10^{-1}	9×10^{-1}	1.5×10^{-1}
A	90	130	90	90
B	750	480	500	150

Table S2: **Parameters a, b, c, d, A and B for Model** PMAL **for four periods.**

Param.	Period 1	Period 2	Period 3	Period 4
a	1×10^{-9}	1×10^{-9}	1×10^{-9}	1×10^{-9}
b	1×10^{-8}	1×10^{-8}	1×10^{-8}	1×10^{-8}
c	1×10^{-8}	1×10^{-8}	1×10^{-7}	1×10^{-8}
d	1×10^{-7}	1×10^{-7}	1×10^{-6}	1×10^{-7}
A	700	500	100	200
B	750	700	450	450

Table S3: **Parameters a, b, c, d, A and B for Model** TMAL **for four periods.**

Param.	Period 1	Period 2	Period 3	Period 4
a	1×10^{-2}	8×10^{-4}	2.0×10^{-2}	4.7×10^{-3}
b	2×10^{-2}	9.2×10^{-4}	2.5×10^{-2}	4.8×10^{-3}
c	1×10^{-2}	4×10^{-1}	2.0×10^{-2}	1.2×10^{-1}
d	2×10^{-2}	6.4×10^{-1}	2.5×10^{-2}	1.3×10^{-1}
A	800	440	100	420
B	850	580	300	470

Table S4: **Parameters a, b, c, d, A and B for Model** SIR **for four periods.**

Param.	Period 1	Period 2	Period 3	Period 4
a	1×10^3	1.2×10^3	5×10^3	1.1×10^4
b	1.5×10^3	4×10^3	2×10^4	1.9×10^4
c	2×10^5	1.6×10^5	1×10^4	5.5×10^4
d	2.3×10^5	2.1×10^5	5×10^4	5.9×10^4
A	500	200	150	400
B	550	550	350	600

References

[1] Yang H. Epidemiologia da Transmissão da Dengue. Trends in Applied and Computational Mathematics. 2003;4(3):387–396.

[2] Yang H, Macoris M, Galvani K, Andrighetti M, Wanderley D. Assessing the effects of temperature on dengue transmission. Epidemiol Infect. 2009;137(08):1179–1187.

[3] Yang HM. The basic reproduction number obtained from Jacobian and next generation matrices–A case study of dengue transmission modelling. Biosystems. 2014;126:52–75.

[4] Anderson RM, May RM, Anderson B. Infectious diseases of humans: dynamics and control. vol. 28. Wiley Online Library; 1992.

[5] Yang HM, Silveira ASB. The loss of immunity in directly transmitted infections modeling: Effects on the epidemiological parameters. Bull Math Biol. 1998;60(2):355–372.

[6] MATLAB 8.0 and Optimization Toolbox 8.1;. The MathWorks, Inc., Natick, Massachusetts, United States.
